# Estimating nesting habitat characteristics for the Kentish plover (*Anarhynchus alexandrinus*) with the effect of substrate and vegetation using a Bayesian network approach

**DOI:** 10.1371/journal.pone.0325750

**Published:** 2025-06-11

**Authors:** Dong-Yun Lee, Ju-Hyun Lee, Jong-Ju Son, Seung-Jun Oh, Ha-Cheol Sung

**Affiliations:** 1 Department of Biological Sciences, Chonnam National University, Gwangju, Republic of Korea; 2 School of Biological Science and Biotechnology, Chonnam National University, Gwangju, Republic of Korea; 3 Research Center of Ecomimetics, Institute of Sustainable Ecological Environment, Chonnam National University, Gwangju, Republic of Korea; King Fahd University of Petroleum & Minerals, SAUDI ARABIA

## Abstract

Coastal habitats play an important role in the nesting ecology of shorebirds; however, these habitats are increasingly threatened by human activity and ongoing habitat loss. The conservation of shorebird populations thus necessitates understanding the utilization pattern of artificial coastal habitats by these birds. Substrate particle size and vegetation cover are key environmental factors influencing the nest site selection and nest success in ground-nesting shorebirds such as plovers. This study aimed to investigate the impact of substrate particle size and vegetation cover for the Kentish plover (*Anarhynchus alexandrinus*) nesting sites within an artificial coastal environment, the Saemangeum reclaimed land. Geological criteria and 1-m^2^ quadrat photos were used to develop Bayesian network (BN) models to analyze the impact of these variables on nest site selection and nest success in 2020. The BN models predicted the impact of substrate particle size and vegetation cover on the likelihood of nest presence and nest success. The results indicated that Kentish plovers prefer sandy sites with moderate vegetation cover and achieve higher nest success in habitats with mixed soil types, including medium (0.25–0.5 mm), fine (0.125–0.25 mm), and very fine (0.063–0.125 mm) sand, along with small proportions of mud (<0.063 mm). These findings highlight the importance of evaluating the complex interactions between plover nests and substrate characteristics, including soil porosity and permeability. Vegetation cover must also be managed with attention to the trade-offs involved, such as predation risk, nest camouflage, crypsis, and thermoregulation which influence plover nesting preference and success. This study provides valuable quantitative insights and emphasizes the need for incorporating multi-layered ecological factors along with inherent uncertainties in coastal environments to restore appropriate artificial coastal habitats for shorebird conservation.

## Introduction

Coastal areas are vital breeding habitats for shorebirds; however, ongoing coastal development significantly impacts these habitats, leading to a decline in shorebird populations [1]. To ensure the conservation of shorebird populations, it is essential to consider strategies for restoring developed artificial coastal areas. Such habitats can serve as breeding habitats for shorebirds [2]. In the Asia-Pacific region, several shorebird species have been frequently observed utilizing artificial coastal areas as alternative habitats [3]. However, prior to implementing the creation or restoration of artificial coastal habitats, it is essential to examine the relationship between environmental factors and nesting site preferences of ground-nesting shorebirds. Such investigations can provide valuable information for restoration and conservation projects in coastal habitats, such as dunes and beaches [4]. Environmental factors in coastal areas influence the nest site selection and nest success of ground-nesting shorebirds in various ways [5,6].

Most plover species in the family Charadriidae are ground-nesting shorebirds, and their nesting site preferences are closely associated with environmental factors [6]. These birds prefer coastal areas with specific habitat characteristics, such as certain substrate particle size and optimal ranges of vegetation cover. The nesting preferences of each plover species influence the patterns of nest site selection and determine nest success or failure [7–10].

This study aimed to investigate the nesting habitats of the Kentish plover (*Anarhynchus alexandrinus*), a species that nest along coastal areas, such as beaches and estuaries [11,12]. Kentish plovers are known to be influenced by various environmental factors, such as habitat landscape and climatic conditions [13]. The population of this species is projected to decline at a higher rate as a consequence of both anthropogenic factors and climate change. Given these challenges, the conservation of Kentish plovers is vital, not only for the species itself but also because its habitats serve as umbrella habitats that support other shorebird species [14]. Indeed, the breeding sites of Kentish plovers often overlap with those of other shorebirds [15,16]. Furthermore, there is an ongoing effort to designate the Kentish plover as an indicator species for tidal flats within the context of coastal environments in South Korea [17]. Therefore, this study aimed to investigate the microhabitat characteristics of Kentish plovers in coastal areas of South Korea, examining their relationships with substrate composition [18–22] and vegetation cover [23]. Additionally, we examined other environmental factors, including shell cover, the presence of artificial objects, and distance from water sources to determine whether these variables have a greater effect on nest site selection and nest success than substrate and vegetation.

Standardized classification systems for substrate particles, based on geological criteria, are instrumental in elucidating the substrate characteristics of plover nesting habitats. Previous studies on plover habitats have established particle size criteria in disparate ways, with the specific criteria varying depending on their research objectives [7,9]. However, to provide more accurate information regarding particle size for the restoration and conservation of plover habitats, it is essential for researchers to collaborate with geologists, including soil scientists. In geology, the classification and categorization of grain particle size have been well-established for over a century [24,25]. These classification systems are extensively used within the geological community, including by institutions such as the United States Geological Survey (USGS). Accordingly, we recognized the necessity of incorporating geological criteria in this study and applied them in our analysis of substrate characteristics, specifically focusing on the soil and stone components of habitat of Kentish plovers.

The main study objectives were: (1) to examine the differences in the environmental factors of microhabitat between nest and non-nest sites and successful and unsuccessful nest sites of Kentish plovers; (2) to estimate the characteristics in vegetation cover and substrate particle sizes; and (3) to provide insights into optimal artificial coastal habitats for Kentish plovers using modelling approaches, thus informing habitat restoration and conservation strategies.

## Materials and methods

### Study sites

For the study, an appropriate field survey area was carefully selected. To provide insight into the breeding ecology of Kentish plovers in artificial habitats, field surveys were conducted in artificial coastal habitats. We selected locations known to be used by shorebirds, including Kentish plovers, as habitats. Furthermore, to gain insights into the diverse relationship between Kentish plovers and their surrounding substrate and vegetation, a single coastal and artificial location with diverse substrate and vegetative conditions was selected. If multiple regions had been included in the study, it would have been difficult to ascertain whether observed nesting preferences were due to regional differences or environmental factors. Hence, a single location with diverse conditions was selected.

**Fig 1:**
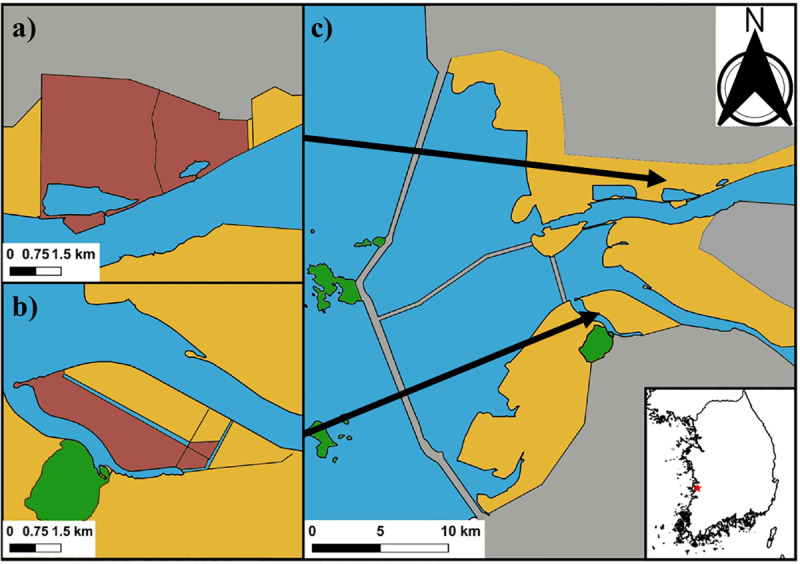
Study areas in Saemangeum, South Korea. Four colours indicated the characteristics of each area (Grey: anthropogenic areas like residential areas, agriculture areas, dykes, and bridges; Green: natural areas; Light brown: reclaimed lands by Saemangeum projects; Dark brown: the research area of this study in reclaimed lands by Saemangeum projects). We conducted field surveys on (a) Mangyeong estuary, Gunsan City, Jellabuk-do Province (35°52’N, 126°40’E) and (b) Dongjin estuary, Buan County, Jellabuk-do Province (35°48’N, 126°38’E) in the Saemangeum area (c). A total of 60 nests were detected in the Mangyeong estuary and 35 nests in the Dongjin estuary during the 2020 breeding season. The base map of South Korea indicates the location of our study areas with the red star (c). We obtained the open source base map data was obtained from the National Geographic Information Institute, South Korea (https://www.ngii.go.kr/kor/main.do). Administrative boundary shape-files were derived from the National Geographic Information Institute (NGII 2020), Republic of Korea, open under KOGL Type 1 license (which permits commercial reuse and redistribution with attribution).

This study was conducted from March to July 2020 at the following estuaries in Saemangeum (Fig 1): (1) Mangyeong estuary (1,011 ha), Gunsan-si, Jeollabuk-do (35°52’N, 126°40’E) and (2) Dongjin estuary (893 ha), Buan-gun, Jeollabuk-do (35°48’N, 126°38’E). These two areas in Saemangeum were selected due to their key habitat characteristics suitable for nesting shorebirds such as Kentish plovers, including wide artificial sandy areas.

Over ten thousand shorebirds use the Saemangeum area as a stopover site along the East Asian–Australasian Flyway during every migration season, and Kentish plovers are regularly observed during both migration and breeding seasons [26,27]. Saemangeum is located between the Seocheon and Gochang tidal flats which are a UNESCO World Natural Heritage Site. However, ongoing and progressive reclamation of these areas adversely impacts these vital habitats [1].

The study sites in Saemangeum were originally developed for agricultural use and are currently inaccessible to the general public due to national development activities [28,29]. Accordingly, research permission was granted by the Saemangeum Project Office in the Korea Rural Community Corporation, which is the authorization to manage land access in the Saemangeum (Official Document Number: College of Natural Sciences-984, January 23, 2020). There was minimal human interference in the study area due to the temporary cessation of development activities; consequently, a wide variety of shorebirds were observed.

The research sites consisted of land parcels separated by roads (paved using asphalt, concrete, gravel, and sand). These parcels exhibited disparate environmental characteristics with a range of features within each parcel such as mixed dense vegetation on one side and sandy areas on the other. Halophytic plants were widely distributed in the study areas dominated by species like *Suaeda japonica* and *Salicornia europaea*, whereas the reclaimed lands were dominated by *Spergularia marina*, *Phacelurus latifolius*, *Phragmites australis*, and *Calamagrostis epigejos*. These areas will likely experience ecological shifts, with salt marshes and exposed sandy areas transitioning into grasslands [29].

### Survey methods

To determine the nest fate of Kentish plover, we utilized two methods: egg floatation and observational evidence of nest success or failure. For each targeted nest, the estimated hatching date were determined using the flotation method with warm water (37–39 °C), a bottle, and a towel [30]. Subsequently, nests were monitored at least twice per week to assess for nest fate. Criteria for identifying nest success or failure were based on indicators reported in previous studies [22,31,32].

### Data collection methods

To document substrate composition and vegetation cover within a 1 m² area, we captured 1-m² quadrat photographs using the rear camera of a Samsung Galaxy S9 (12 MP, OIS, f/1.5–2.4 dual aperture) from a height of 1.5 m. For each nest, two types of quadrant photographs were captured: (1) a photograph centred on the nest (representing the nest site) and (2) two photographs captured at randomly selected locations approximately 10 m from the nest (representing non-nest sites). If a randomly selected site was located near another Kentish plover nest (<1 m), it was excluded and replaced with another site. We estimated the cover rate of eight environmental variables visible within each quadrat photo (Table 1). Substrate particle size was determined according to geological criteria [24,25]. Detailed vegetation species composition data were not included in this study, as vegetation cover rate plays a much more significant role [23]. To ensure consistency, a single observer was assigned to measure the cover rate of variables. Photo analysis was performed using the Scalar imaging system, version 2.88.

**Table 1:** Variables used in the nest site selection model. All variables were measured in the 1-m^2^ quadrat of nest sites and non-nest sites.

Variable	Discretizing method	Definition
Dry vegetation	Median and quartiles	Dead vegetation, such as dry twigs and grass
Live vegetation	Median and quartiles	Live vegetation when researchers took the picture of the nest
Total vegetation	Median and quartiles	All vegetation, whether dry or live
Shell	Median and quartiles	Covered with seashells of bivalves or gastropods
Object	Presence or absence	Other objects, such as vinyl and plastic
Sand	Median and quartiles	Sand particle size < 2 mm
Granule + pebble	Presence or absence	2 mm < Granule particle size < 4 mm 4 mm < Pebble particle size < 64 mm (Granules or pebbles of these sizes were less in our study. Thus, we counted the grains in these two size categories together.)
Cobble	Presence or absence	64 mm < Cobble particle size < 256 mm

**Fig 2:**
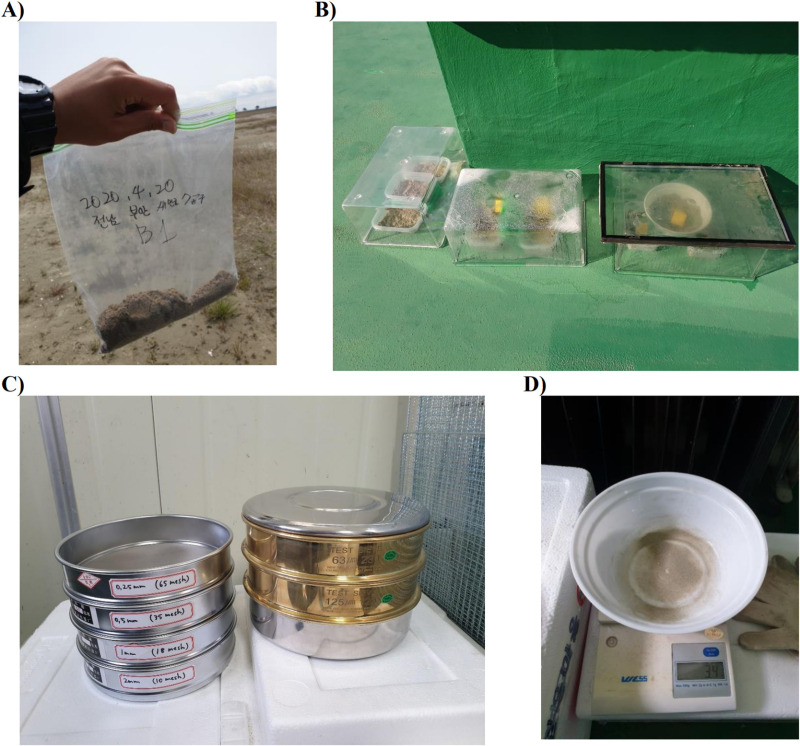
Examples of substrate particle size analysis. (a) A soil sample was collected 30 cm from a nest. (b) Soil samples were placed in an acrylic cage to dry in the sunlight for at least four hours. (c) The dried samples were sieved to classify soil particles by size. (d) The total weight of the sieve residues was compared with the weight of the original sample.

To investigate nest characteristics beyond those in the quadrat photographs, we measured the distance from each nest to two environmental factors (vegetation and water source) and collected soil samples from each nest site (Table 2). First, the distance from a nest to the nearest vegetation (m) was measured directly in the field. Additionally, the GPS location of the target nests was recorded using a satellite map (Kakao map) to calculate the distance from each nest to the nearest water source (m). Second, the soil particle size of each nest site was analyzed through granulometric analysis using a fine-scale than that used in the quadrat photo analysis (Fig 2). Soil samples were collected at 30 cm from each nest, following the method used in a previous study investigating the relationship between soil particle sizes and nest site selection in the Campo Miner (*Geositta poeciloptera*) [33]. Samples were collected at a depth of 2–3 cm, considering the average nest depth of 2.76 ± 0.57 cm reported in a previous study [34]. Before analyzing particle size analysis, soil samples were air-dried in an acrylic cage under sunlight for at least four hours, as sieving wet soil was difficult due to soil cohesion. The dry samples were sieved to classify soil particles by size, using a standard sieve set with ASTM mesh sizes: #10 (2.00 mm), #18 (1.00 mm), #35 (500 µm), #65 (250 µm), #120 (125 µm), and #230 (63 µm). The total weight of sieve residues was compared with the weight of the original sample. If there was more than a 2% discrepancy, the sample was remeasured to reduce the error rate [35]. The particle size distribution in this study was classified based on previously reported geological criteria [24,25].

**Table 2:** Variables used in the nest fate model. These variables were measured for each nest site.

Variable	Discretizing method	Definition
Distance between a nest and the nearest vegetation	Median and quartiles	Distance between a nest and the nearest vegetation (directly measured in the field)
Distance between a nest and water source	Median and quartiles	Distance between a nest and the nearest water source (measured using GPS and satellite map [Kakao map])
Nest group based on cover rate on nests in 1-m² quadrat	Clustering	Cover type within 1 m^2^ of the nest site (dry vegetation, live vegetation, shell, sand, granule + pebble, cobble, and object)
Nest group based on soil particle size	Clustering	Soil particle type in the nest site (gravel: > 2 mm, very coarse sand: 1–2 mm, coarse sand: 0.5–1 mm, medium sand: 0.25–0.5 mm, fine sand: 0.125–0.25 mm, very fine sand: 0.063–0.125 mm, and mud: 0.063 mm)

### Modelling process and statistical analysis

We developed models to predict nest presence or absence and success or failure based on environmental factors, such as substrate type (particle size) and vegetation cover. First, the substrate characteristics of nest sites were examined to identify particle sizes with the most significant influence on nest site selection and nest success in Kentish plovers. Furthermore, differences in the distribution of particle sizes between nest and non-nest sites, as well as between successful and unsuccessful nest sites were assessed. To evaluate the influence of finer-scale variation, the proportion of each particle size category was analyzed in relation to nest success. Second, we investigated the impact of vegetation cover on nest site selection and nest success of Kentish plovers. Given the complex relationships involved, we refrained from drawing direct conclusions regarding the effects of vegetation. Instead, we described the differences in vegetation cover between nest and non-nest sites, as well as between successful and unsuccessful nest sites, using a modelling approach.

We employed a Bayesian network (BN) modelling approach, which offers several advantages, including direct graphical representation, structural learning, and the integration of diverse data sources [36]. This method has been successfully applied in previous studies on the Piping plover (*Charadrius melodus*) to identify key habitat factors and inform conservation planning [37–39].

The present study used the BN model. First, the predictions of the BN model were based on conditional possibilities, reflecting the relationships between pre- and post-events. Next, the BN model structures used in ecological research were constructed using various algorithms (e.g., Grow-Shrink, Hill-Climbing, and Tabu.) and ecological information with sufficiently large sample sizes.

We used R software, version 4.3.1 [40], to perform cluster analysis and build models using the BN model. Before the BN modelling process, we discretized all variables to define ‘nodes’: (1) median and quartiles, (2) presence/absence, and (3) clustering. We preferred the median and quartiles method for each variable over other discretization methods. However, if a variable exhibited too many zero values, we discretized it based on considering presence/absence. For variables that showed minimal variation and were not linked to other variables through ‘arcs’, we used cluster analysis. We conducted K-means cluster analysis using the *factoextra* package v1.0.7 [41]. To determine the optimal number of clusters, we referred to graphs generated using the elbow and silhouette methods and then compared the average values of variables among clusters. Each clustering group within a variable was labelled using an alphabet or number.

To construct BN models, we first created a primitive model using the Hill-climbing (HC) algorithm based on the Bayesian information criterion (BIC) score, using the *bnlearn* package v4.6.1 [42]. We designated a location node as the first node and either the nest designation or nest fate nodes as the last. Considering the original causal relationships between variables, we made manual adjustments to it based on HC models (e.g., dry and live vegetation rates always influenced total vegetation rates, rather than the reverse). We described the strength values of arcs and the BIC scores of nodes. Additionally, we evaluated the sensitivity and accuracy of each model algorithm. To assess model sensitivity, we used the receiver operating characteristic (ROC) curve to estimate the area under the ROC curve (AUC) value (at least over 0.5 AUC value) [43]. To evaluate model accuracy, we calculated the expected loss of the models using 10-fold cross-validation and applied classification error as the loss function (0 = no error, 1 = complete error) [44].

We constructed and trained two models: (1) the nest site selection model (comparing nest and non-nest sites) and (2) the nest fate model (comparing successful and unsuccessful nests). First, we developed the nest site selection model for Kentish plovers using variables from the 1-m² quadrat photographs (Table 1). We incorporated the following components: (1) a node representing nest site designation, (2) a node indicating research location, (3) three nodes indicating the presence/absence of granule + pebble, cobble, and object, and (4) five nodes representing discretized continuous cover rate variables, namely sand, shell, dry vegetation, live vegetation, and total vegetation cover (dry vegetation + live vegetation), categorized using median and quartiles. We then predicted the 100% possibility models for (1) nest/non-nest site and (2) the most related substrate/vegetation variable cover (S2A Fig in S1 File). Second, we constructed the nest fate model of Kentish plovers using five variables (Table 2), comprising the following: (1) a node representing the nest fate (nest success and failure), (2) a node representing the research location, (3) two nodes representing discretized continuous variables, distance from the nest to the nearest vegetation and distance from the nest to water, categorized based on the median and quartiles of each variable, and (4) two nodes, indicating cover type and soil type, which characterize the nest environment. Cover types were grouped based on seven cover variables in the quadrat photos (dry vegetation, live vegetation, sand, granule + pebble, cobble, object, and shell; S2B Fig in S1 File). Soil types were grouped based on seven particle size variables (gravel, very coarse sand, coarse sand, medium sand, fine sand, very fine sand, and mud; S2C Fig in S1 File). We then predicted the 100% possibility models for (1) nest success/failure and (2) cover/soil type classification (S2D Fig in S1 File).

We conducted Principal Component Analysis (PCA) using the *stats* package [40] to examine the relationships between nest failure causes and environmental variables (S2E Fig in S1 File). Before PCA, we performed Spearman’s correlation tests to prevent multicollinearity among highly positively correlated variables using the *psych* package [45].

### Ethics statement

The first author completed a formal training program on animal welfare and ethical research practices (Certificate No. CNU IACUC-2020–107), issued by Chonnam National University (CNU) and the CNU Laboratory Animal Research Centre, to ensure adherence to ethical standards during the research process. No approval from a research ethics committee was required for this study, as no birds were captured, handled, or manipulated. Despite not requiring formal approval, all data were collected using non-invasive methods in strict compliance with the current laws and regulations of South Korea to minimize disturbance to wildlife. A safe distance was maintained during observations of incubating individuals using binoculars (Nikon Prostaff 8 × 42) and a scope (Nikon Prostaff 5 field scope 82-A & SEP-20–60) to prevent interference. Nesting data collection was initiated only in the absence of incubating birds on the target nest, and all data were collected within a five-minute period to minimize disturbances.

## Results

**Fig 3:**
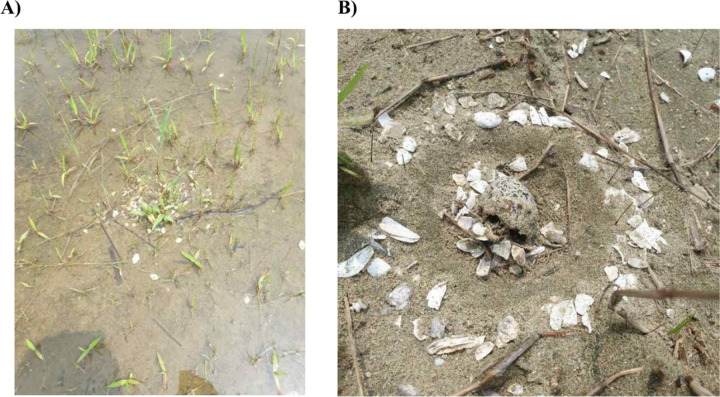
Examples of unsuccessful Kentish plover nests in Saemangeum, 2020. (a) Flooding events were the main cause of nest failure in this study. (b) Kentish plover nests were often depredated by predators.

Of the 101 Kentish plover nests found in the study area, we used a dataset of 95 nests for analysis, as six nests in Mangyeong estuary were considered outliers (three located on a rice field ridge paved with gravel and three discovered at the hatching date). A total of 60 nests were in the 21 land parcels of Mangyeong (33 successful and 27 unsuccessful nests), and 35 nests were located in the 12 land parcels and an exposed dry riverbed of Dongjin estuary (28 successful and 7 unsuccessful nests). Among the 34 failed nests, the causes of failure were flooding in 21 nests, unknown causes in 11 nests, and depredation in two nests (Fig 3).

We captured 285 quadrat photographs (95 from nest sites and 190 from non-nest sites), which were used to create the nest site selection model for Kentish plovers. To construct the nest fate model, we used a dataset that included quadrat photographs, soil particle size, and distance data. In addition, we observed that many other shorebirds including the Little tern (*Sternula albifrons*) and the Eurasian oystercatcher (*Haematopus ostralegus*), bred in the study areas. We also observed various terrestrial predators such as the free-ranging dog (*Canis familiaris*) and the common raccoon dog (*Nyctereutes procyonoides*), as well as, aerial predators such as raptors (*Falco tinnunculus*, *F. peregrinus*, *F. subbuteo*, *Circus cyaneus*, and others) and owls (*Bubo bubo*). Egg predators, such as herons (*Ardea cinerea*, *A. coromandus*, *A. alba*, and others), were also observed.

### Nest site selection model

**Fig 4:**
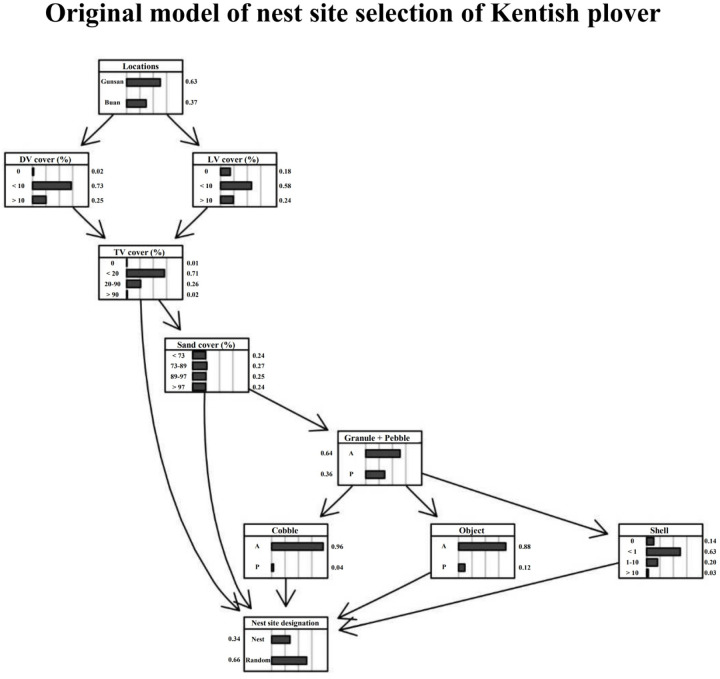
Bayesian network model for nest site selection of Kentish plovers (95 nest site and 190 non-nest site data). This model was constructed using eight environmental factors from 1-m^2^ quadrat photographs. Arrows denote the direction of conditional dependencies (arcs). Boxes indicate nodes (black bars in each node represent the probability of each category in a variable). DV = dry vegetation, TV = total vegetation, LV = live vegetation, A = absence, P = presence.

The nest site selection model for Kentish plovers demonstrated the relationships among eight environmental factors, a locality variable and a nest site designation factor (Fig 4). The ROC curve showed an AUC value of 0.65, with an expected loss rate of 0.33. The highest strength value was observed in the arc from total vegetation cover to nest site designation, whereas the lowest was from total vegetation cover to sand cover (Table 3). Among the nodes, the nest site designation node had the lowest BIC score, but the cobble node had the highest score (Table 4).

**Table 3:** Strength values of arcs in the nest site selection model. Higher values indicate greater data fitness and importance within the model.

Arc			Arc strength values
TV cover	**→**	Nest site designation	534.14
Shell cover	**→**	Nest site designation	528.30
Sand cover	**→**	Nest site designation	520.14
Cobble	**→**	Nest site designation	359.04
Object	**→**	Nest site designation	356.49
LV cover	**→**	TV cover	1.06
Granule + pebble	**→**	Object	−0.02
Locations	**→**	DV cover	−3.23
Granule + pebble	**→**	Shell cover	−6.92
Granule + pebble	**→**	Cobble	−7.88
Sand cover	**→**	Granule + pebble	−8.10
Locations	**→**	LV cover	−34.70
DV cover	**→**	TV cover	−42.82
TV cover	**→**	Sand cover	−95.20

TV, total vegetation; DV, dry vegetation; LV, live vegetation.

**Table 4:** The Bayesian information criterion (BIC) values of nodes in nest site selection model. Lower values indicated the higher necessity in a model.

Node	BIC value
Nest site designation	−870.73
Sand cover (%)	−308.23
Shell cover (%)	−284.27
LV cover (%)	−247.04
DV cover (%)	−190.84
Locations	−190.39
Granule + pebble	−180.01
TV cover (%)	−139.89
Object	−104.96
Cobble	−38.27

TV, total vegetation; DV, dry vegetation; LV, live vegetation.

This BN model revealed key distinctions between nest site and non-nest site models. First, in the nest site BN model for Kentish plovers (S1A Fig in S1 File), Kentish plovers preferred sandy areas (< 73% sand cover, 30% possibility; 73–89% sand cover, 33% possibility) with some vegetation cover (0–20% total vegetation cover, 66% possibility) rather than only sandy areas (> 97% sand cover: 10% possibility). Second, in the non-nest site BN model (S1B Fig in S1 File), the possibility of the < 20% category was the highest in the total vegetation cover node. Additionally, in the sand node, the possibilities of the 89–97% and >97% categories were higher than those of the < 73% and 73–89% categories. Other variables did not show large differences between the two models.

Next, the BN model showed the differences among cover rates. First, in the sand cover BN models for Kentish plovers, the possibility of a nest site in the nest designation node was over 40% in low sand cover models and lower in high sand cover models (S1C Fig in S1 File). Second, in the total vegetation cover BN models, the possibility of a nest site in the nest designation node was highest in the 20–90% total vegetation cover model and lowest in the < 20% total vegetation cover model (S1D Fig in S1 File).

Lastly, the model demonstrated local variations in environmental cover rates (S1E Fig in S1 File). The vegetation cover rate in Gunsan was higher than in Buan, whereas the sand cover rate was lower.

### Nest fate (nest success and failure) model

**Fig 5:**
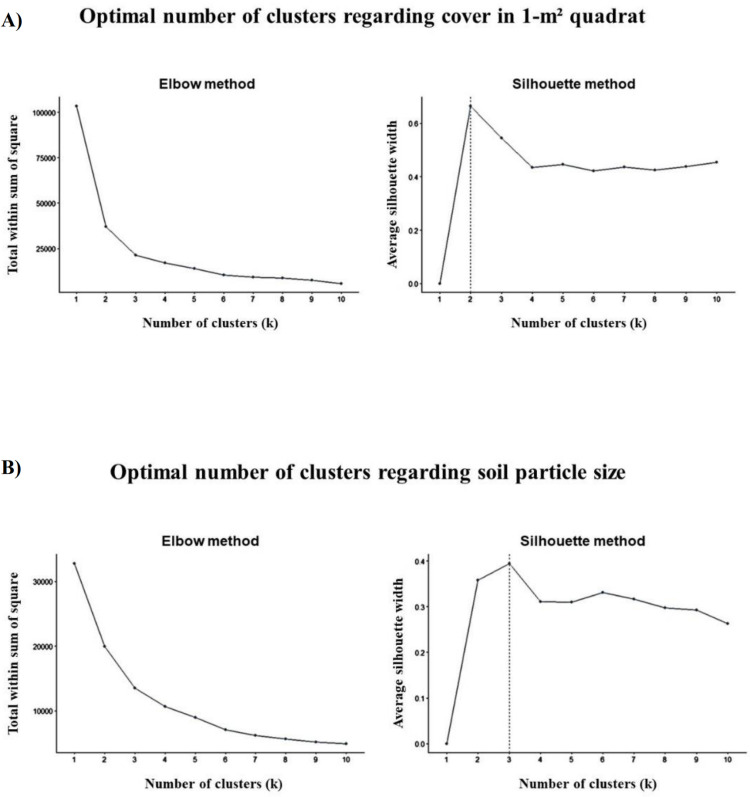
Results of the elbow and silhouette methods for cluster analysis. The optimal number of clusters could not be determined using the elbow method. Therefore, we referred to the silhouette method and set two clusters for cover rate factors (A) and three clusters for soil particle size factors (B).

**Fig 6:**
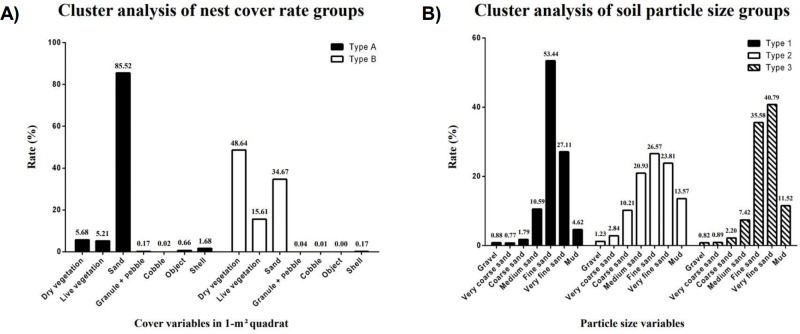
Cluster analysis of cover rate in 1-m2 quadrat and soil particle size. (A) In type A, sand cover was at the highest rate, followed by dry vegetation. In type B, dry vegetation was at the highest cover rate, followed by sand. (B) In types 1 and 2, fine sand was the main component, followed by very fine sand. In type 3, very fine sand was the main component, followed by fine sand.

For cluster analysis, we discretized two variables; cover and soil types. The optimal number of clusters was determined based on the results of the silhouette and elbow methods (Fig 5). First, the cover rates of nests in the 1-m^2^ quadrat were grouped into two categories (types A and B, Fig 6A). In the type A group, sand cover was the highest, followed by dry vegetation cover (sand, 85.52%; dry vegetation, 5.68%). In the type B group, dry vegetation cover was the major variable, followed by sand cover (dry vegetation, 48.64%; sand, 34.67%). Second, the soil particle size of the nests was grouped into three categories (types 1, 2, and 3, Fig 6B). In the type 1 group, fine sand was the major variable, with very fine sand, as the second major variable (fine sand, 53.44%; very fine sand, 27.11%; medium sand, 10.59%). The type 2 group exhibited similarities to the type 1 group, although the differences between particle type rates in the type 2 group were smaller than in the type 1 group (fine sand, 26.57%; very fine sand, 23.81%; medium sand, 20.93%). In the type 3 group, very fine sand was the major variable, followed by fine sand (very fine sand, 40.79%; fine sand, 35.58%). The mud rate was the lowest in type 1 (4.62%) but exceed 10% in type 2 (13.57%) and type 3 (11.52%).

**Fig 7:**
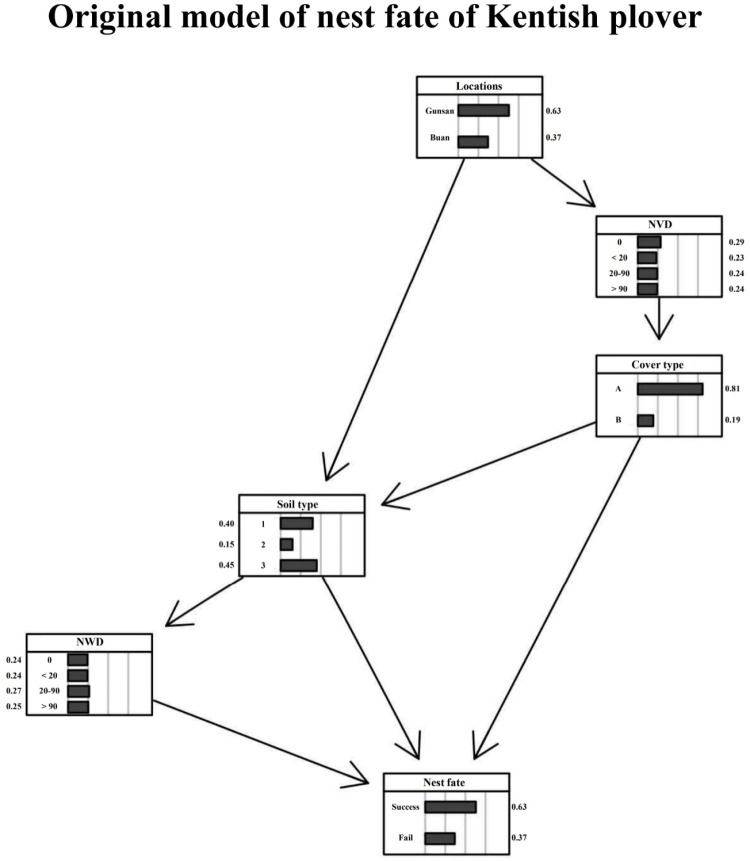
Bayesian network model of nest fate in Kentish plovers, prepared using data from 61 successful nests and 34 unsuccessful nests. Four environmental factors were used. NVD = distance between a nest and the nearest vegetation, NWD = distance between a nest and a water source.

The nest fate BN model for Kentish plovers consisted of four variables, along with a locality variable and a nest fate factor (Fig 7). This model had an AUC value of 0.70, with an expected loss rate of 0.33. The strength values were highest in the arc from the distance between a nest and a water source to nest fate and lowest from location to soil type (Table 5). Among the nodes, the lowest BIC score was for the distance between a nest and a water source, whereas the highest was for the cover type node (Table 6).

**Table 5:** Strength values of arcs in the nest fate model. Higher values indicate better data fitness and greater importance in the model.

Arc			Arc strength values
NWD	**→**	Nest fate	35.15
Soil type	**→**	Nest fate	29.41
Cover type	**→**	Nest fate	23.93
Cover type	**→**	Soil type	−0.33
Soil type	**→**	NWD	−2.19
NVD	**→**	Cover type	−2.54
Locations	**→**	NVD	−3.98
Locations	**→**	Soil type	−16.88

NWD: distance between a nest and water source.

NVD: distance between a nest and the nearest vegetation.

**Table 6:** Bayesian information criterion (BIC) values of nodes in the nest fate model. Lower values indicate higher necessity in the model.

Node	BIC value
NWD	−136.28
NVD	−134.25
Nest fate	−104.36
Soil type	−83.05
Locations	−64.80
Cover type	−45.86

NWD: distance between a nest and water source.

NVD: distance between a nest and the nearest vegetation.

The BN model demonstrated the distinctions between the two models of nest success and failure (S1F Fig in S1 File). First, in the nest success BN model of Kentish plovers, were associated with areas dominated by sand (type A) with fine and very fine sand (type 3). Second, in the nest failure BN model, type A and type 1 showed a higher possibility of failure than type B, type 2, and type 3.

Cover and soil types differed in each BN model. First, in the cover type BN models, the possibility of nest success in the nest fate node was lower in the cover type A model than in cover type B (S1G Fig in S1 File). Second, in the soil type BN models, the possibility of nest success in the nest fate node was lower in the soil type 1 model than in the soil type 2 and 3 models (S1H Fig in S1 File). Finally, NVD and soil type nodes exhibited larger differences depending on location. Soil type 2 was absent (0%) in Gunsan (S1I Fig in S1 File).

### Relationships between nest failure causes and environmental variables

**Fig 8:**
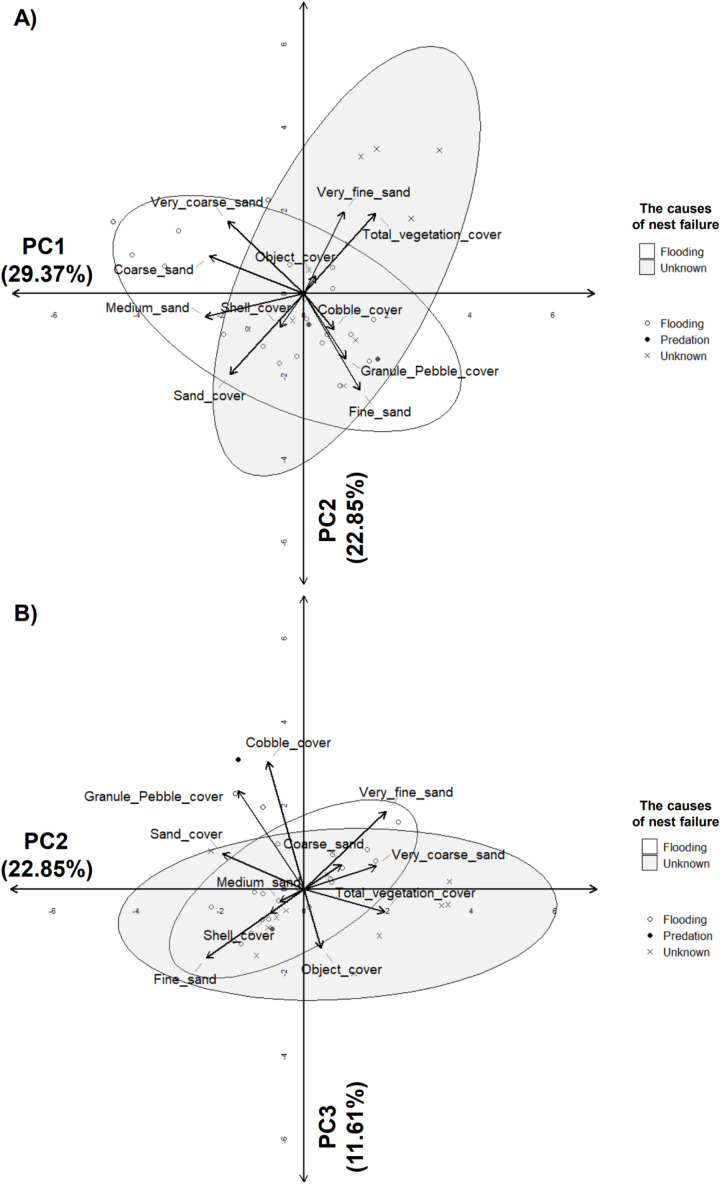
Principal Component Analysis (PCA) of nest failure causes and environmental factors. Flooding events were largely related to soil particle sizes. In particular, coarse, medium, and fine sand showed the relationships with flooding in both graphs. However, unknown failure causes were associated with shell and vegetation covers.

A total of 11 variables were used, as dry and live vegetation were highly positively correlated with total vegetation, gravel with very coarse sand, and mud with very fine sand (*r* > 0.70; *p* < 0.05). Based on PC1 to PC3 (% of total variance = 63.83%; Table 7), flooding correlated with most soil particle sizes in the PC1 and PC2 graph (Fig 8A). However, among soil particle sizes, only coarse, medium, and fine sand showed relationships with flooding in the PC2 and PC3 graph (Fig 8B).Unknown nest failure causes were associated with most cover variables including vegetation and shell cover, in both graphs (Fig 8).

**Table 7:** Eigenvalues and variance in the PCA result of nest failure causes and environmental variables.

Eigenvalues	% of variance	% of cumulate variance
3.23	29.365	29.365
2.51	22.852	52.217
1.28	11.612	63.928
1.08	9.792	73.621
1.07	9.71	83.338

## Discussion

Our study revealed that Kentish plovers prefer sandy coastal areas with moderate vegetation cover for nesting, and their nest success is directly influenced by specific particle sizes. Our BN models, in particular, highlight the importance of maintaining a balance among environmental factors at breeding sites, as this affects both nest site selection and nest success. In addition, flooding, the main cause of nest failure, was associated with specific soil particle sizes. These findings suggest the need for coastal habitat management strategies that consider the balance between substrate types and vegetation cover to ensure the breeding success of Kentish plovers.

### Effect of substrate types and particle size on nest site selection and nest success of Kentish plovers

We found that substrate types exhibited the closest and direct relationships with the nest site selection and nest success of Kentish plovers. The BN models showed sand cover had a greater influence on nest site selection than other substrates, such as shell, gravel, pebbles, and cobbles. This result aligns with findings from previous studies [18,21,46]. In particular, the models revealed that soil particle size correlates with the nest success of Kentish plovers. The possibility of nest success was higher when habitats consisted of a mixture of medium sand to mud, in addition to only fine and very fine sand. PCA analysis exhibited a link between flooding related nest failure and soil particle size. Therefore, we assume that soil particle sizes may affect nest success; further research is required to clarify the precise mechanisms by which soil particle size influences Kentish plover breeding. One possible explanation is that soil permeability and porosity may affect the nest success of Kentish plovers, as our observations indicated that flooding caused by heavy rainfall was the predominant cause of nest failure. Tidal effects were not considered in this study because the study areas in Saemangeum remain above sea level regardless of tidal fluctuations (high or low, neap or spring). In these areas, only rainfall and drainage influenced flooding events. Additionally, the presence of mud as a fine substrate could negatively impact nest survival due to low soil permeability, which increases flooding occurrences [20]. In contrast, the BN models indicated that an insufficient mud composition in nesting habitats negatively affects the success of Kentish plover nests. Thus, future studies should further investigate the relationship of soil permeability and porosity with nesting success of plovers, incorporating data regarding on soil sample volumes, which we did not obtain, along with pore space measurements [47–49].

**Fig 9:**
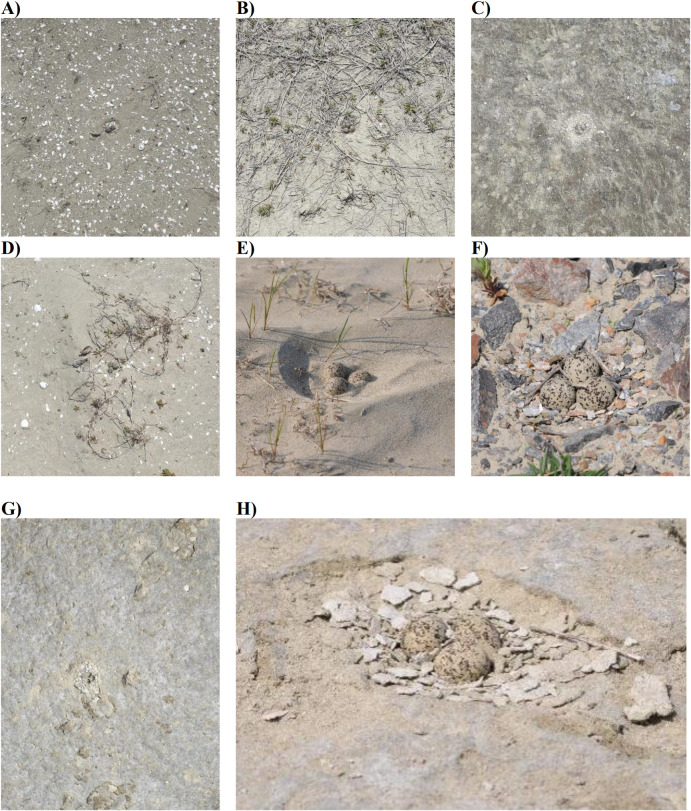
Examples of nest types of Kentish plovers. We categorized the nests into five types: (a) primarily composed of shells, (b) primarily composed of dry vegetation main, (c) composed of dry soil particles, (d) composed of mixed materials, and (e) primarily composed of sand. (f) We also identified three nests located on a rice field ridge paved with gravel. (g–h) Additionally, we observed a nest built within human footprints. A pair of Kentish plovers selected a human footprint for nesting on an exposed, arid riverbed, primarily using soil particles as nest materials.

Despite our detailed analysis of substrate characteristics, we cannot conclude that soil composition, specifically the proportion of mud and sand, is an important determining factor for Kentish plover nesting sites. This is because adult Kentish plovers used various materials for nest construction, including shells, dry vegetation, and dry soil particles (shells were used in 18 nests; dry vegetation in 33 nests; dry soil particles in 5 nests; a combination of two or more major types of material in 22 nests; and predominantly sand in 17 nests; Fig 9). Furthermore, three pairs of Kentish plovers selected nest site positions on rice field ridges paved with gravel (Fig 9F), though this data was excluded from our analysis. In other locations, Kentish plovers have been observed nesting in the shingle beaches of northern France [50], the rocky coasts of north-western Morocco [19], and shell-covered saltpans of southern Spain [16]. Thus, the influence of other substrate types, in addition to sand, on Kentish plover’s nest site selection required further investigation.

### Effect of vegetation cover on nest site selection and nest success of Kentish plovers

According to our models, Kentish plovers are more likely to select sandy habitats with moderate vegetation cover for nesting than solely sandy habitats, which was an unexpected result. Initially, we hypothesized that Kentish plovers would prefer sandy areas with no vegetation over those with some vegetation cover for nesting. When considering only nest site and success models, Kentish plovers were found to prefer habitats with low vegetation cover, and their nest success was higher in these habitats. This may be because reduced vegetation allows incubating Kentish plovers to detect approaching predators more easily [31]. However, in this study, when the possibility of vegetation was designated as ‘present’, the possibilities of nest site and success were higher compared to when vegetation was designated as ‘absent’. Additionally, the possibility of a nest site selection being selected for nesting was equal when the possibilities of vegetation were set as either ‘absent (0%)’ or ‘dense (>90%)’.

Based on our modelling results, we propose that ‘survivorship bias [51, 52]’ may explain the negative impact of vegetation on the nest site selection of plovers. To be specific, exposed nests are likely easier for terrestrial predators to detect than those situated near vegetation. For example, in this study, we discovered three nests only their hatching dates. Although we frequently visited and observed the same areas every two days, dense vegetation concealed these nests, preventing their earlier detection. Additionally, in densely vegetated locations, we often discovered Kentish plover nests only when we were about to step on them. These observations suggest that vegetation may safeguard against from predators, including humans.

Several hypotheses have been proposed to explain the positive relationship between vegetation cover and Kentish plover nests. First, vegetation can provide protection for nests and incubating plovers from predators [53]. Vegetation cover may reduce the ability of incubating adults to detect predators because of visual interference; conversely, vegetation helps conceal incubating adults from predators. This suggests a trade-off between nest crypsis and predation risk [54]. In this study, we recorded two depredated nests and 11 nests for which the cause of nest failure was unknown. If predators consumed the eggs from these 11 nests, no visible traces, such as partial egg fragments, would have remained, as some predators swallow eggs whole [46,55]. Furthermore, the PCA results indicated a relationship between nest failure of unknown cause and vegetation cover, which strongly influences predation events and nest crypsis. These findings suggest that predation was the second leading cause of nest failure, following flooding in this study. Second, vegetation cover may provide more stable thermal conditions for eggs compared to fully exposed nests [56]. In Kentish plovers, parents demonstrate an ability to sensitively regulate nest temperature, exhibiting a trade-off between the need for nest camouflage and maintaining optimal nest temperatures [57]. Therefore, vegetation cover in Kentish plover breeding sites should be considered a complex factor that contributes to predation risk, nest crypsis, and thermoregulation.

### Recommendations for artificial coastal breeding habitats and future studies

Our findings indicate that the reclaimed land can serve as a breeding habitat for Kentish plovers. The BN modelling results suggest that Kentish plovers prefer nesting sites with moderate vegetation cover, and a mix of various soil particle sizes, rather than habitats dominated by fine sand. Given that flooding was the major cause of nest failure, incorporating drainage systems in the management of artificial habitats is essential. Furthermore, as reported in previous studies and observed in this study, the nesting sites of Kentish plovers are located near those of other shorebirds [16,23], protecting artificial coastal nesting habitats for Kentish plovers can contribute to conservation efforts for other shorebirds [14]. Consequently, insights gained from previous international projects can inform future research, conservation, and restoration efforts by applying the findings from Saemangeum to other regions. For instance, global conservation and restoration projects have emphasized the importance of sand composition and vegetation management. In alignment with this, the Netherlands implemented the Zand motor [58] and Hondsbossche dunes [59] projects, and the USA implemented the Tijuana estuary restoration [60] and Delaware Bay [61] projects to conserve and restore coastal ecosystems.

In modelling artificial habitats for Kentish plovers, our predictions and recommendations are focused on flexibility rather than precise estimations, as would be expected in a generalized linear model (GLM). In particular, ‘uncertainty’ is a key concept in BN models [62]. This concept is distinguished from other models based on the Neyman-Pearson framework as it does not impose strict assumptions and does not exclude variability as errors or outliers. BN models can be a useful tool for interpreting uncertain ecological characteristics through conditional probabilities. This flexibility allows BN models to account for characteristics in dynamic and stochastic ecosystems, without prematurely excluding critical factors. For example, BN models can evaluate the effects of substrate variability and unmeasured relationships, providing well-supported explanations for habitat selection at fine scales. Unlike GLMs, BN models allowed us to identify the trade-off strategies and the complex effects of soil particle size in the nesting patterns of Kentish plovers by analyzing conditional possibility and causality among variables. Our models also provide insights into the optimal habitat characteristics of this species based on the primary preferences of individuals in Saemangeum. Rather than promoting a one-size-fits-all approach, our findings suggest habitat management strategies that balance vegetation cover and mixed soil types to establish conservation and restoration plans. However, the construction of BN models is inherently dependent on the sample size and a prior knowledge. These models can produce skewed results when working with an inadequate dataset or when the basic information is incorrect. Given the limited sample size of unsuccessful nests, we used PCA to examine the relationships between nest failure causes and environmental factors. Furthermore, in addition to constructing BN models, it is necessary to discretize continuous variables into categorical factors. Consequently, to better assess variable tendencies, we recommend employing additional modelling techniques based on the Neyman-Pearson framework, such as GLMs, in conjunction with BN models.

Despite our investigation into the relationship between substrate, vegetation, and the nesting habits of Kentish plovers, further studies are necessary to elucidate the influence of other factors that may affect nest site selection. For instance, the impact of animal and human footprints on the nesting behavior of Kentish plovers should be investigated. Previous studies have shown that successful nests were located farther from mammal footprints than unsuccessful nests, likely due to increased egg predation by terrestrial animals [22,32]. However, interestingly, in this study, a pair of Kentish plovers selected one of our footprints as their nesting site (Fig 9G and H). This suggests that human footprints may exert an anthropogenic influence on the nesting habits of Kentish plovers. Investigating this potential effect is essential before the implementation of conservation strategies for the species’ habitats. Secondly, regional differences in habitats must be studied to determine their impact on nest site selection and nest success in Kentish plovers. We found that local differences within the Saemangeum can influence soil properties, which may, in turn, be linked to the nest success of this species. Kentish plovers are widely distributed from North Africa and Europe to East Asia [63] and inhabit various coastal environments including sandy coasts [23,64], rocky coasts [19,50], saltpans [65], estuaries [46], and reclaimed lands [66]. Our BN models also showed that when location variables were excluded, the nest site selection (0.97) and nest fate (1.00) models exhibited higher AUC values. However, models incorporating location variables exhibited diminished AUC values. This indicated that, except for location variables, our previous models had likely been overfitted, resulting in the shown high AUC values [67]. The reduction in AUC values after including location variables suggests that our models became more generalized and less prone to overfitting. Thus, investigating the local differences of nesting sites is important for this species. Future studies could benefit from cross-regional collaborations comparing habitat preferences across different populations of Kentish plovers or related species globally. Lastly, over time, changes in key coastal environmental factors, such as vegetation cover and substrate composition, may alter shorebird habitats. Thus, long-term monitoring and the implementation of suitable management plans should be undertaken to ensure the consistent conservation of their habitats and breeding sites. These efforts contributed to shorebird conservation and enhance our understanding of habitat dynamics influenced by anthropogenic impacts.

## Conclusion

Our findings suggest that coastal artificial habitats, such as Saemangeum, may offer a potentially suitable environment for shorebird breeding. However, ongoing shorebird habitat modifications due to land reclamation projects have gradually reducing foraging and refuelling sites for shorebirds, ultimately threatening their populations. The extensive loss of tidal flats to reclamation projects in this region necessitates urgent measures to regulate and manage human activities in a manner that prioritizes ecological sustainability to support shorebird breeding populations. The insights gained from this study have significant conservation implications for Saemangeum, where shorebird habitats remain under persistent threat. A scientific approach is essential for mitigating habitat loss and enhancing the capacity of these artificial landscapes to support breeding populations. Our findings underscore the necessity of targeted habitat management that balances substrate composition, vegetation cover, and nest success in reclaimed areas. Moreover, the treat to coastal area extends beyond Saemangeum, as developments worldwide threaten shorebird habitats. Understanding the relationship between environmental factors such as substrate composition and vegetation is the primary step in obtaining quantitative to inform for conservation and restoration efforts in coastal environments.

## Supporting information

S1 File S1 Fig (A) Bayesian network model of nest site of Kentish plovers. We set the possibility of nest site designation at 100% of the nest site. The < 20% category exhibited the highest possibility in the TV cover node and the 73–93% category, in the sand cover node. DV = dry vegetation, TV = total vegetation, LV = live vegetation, A = absence, P = presence. (B) Bayesian network model of non-nest site of Kentish plovers. We set the possibility of nest site designation at 100% of the non-nest site. The < 20% category exhibited the highest possibility in the TV cover node and the > 97% category, in the sand cover node. DV = dry vegetation, TV = total vegetation, LV = live vegetation, A = absence, P = presence. (C) Bayesian network model of sand cover of Kentish plovers. We set the possibility of sand cover at 100% of each category. The BN models demonstrated that the possibility of a nest site in the node of nest site designation decreased as the ambient sand cover increased. (D) Bayesian network model of total vegetation cover of Kentish plovers. We set the possibility of total vegetation cover at 100% of each category. The BN models exhibited that the possibility of a nest site in the node of nest site designation was higher when the ambient total vegetation cover was present to some extent. (E) Bayesian network model of nest site selection of Kentish plovers depending on locations. We set the possibility of each location at 100%. The BN models exhibited the local differences of vegetation and sand cover. (F) Bayesian network model of nest success and fail of Kentish plovers prepared. (A) We set the possibility of nest fate at 100% of nest success. The categories exhibiting the highest possibility of each node were type A (major type: sand) in the cover type node, type 3 (major type: very fine sand) in the sand type node, and the < 2 cm category in the NVD node. The 50 m and >230 m categories showed higher possibility than did the other categories in the NWD node. (B) We set the possibility of nest fate at 100% of nest failure. The categories exhibiting the highest possibility of each node were type A (major type: sand) in the cover type node, type 1 (major type: fine sand) in the sand type node, the < 2 cm category in the NVD node, and the 140–230 m category in the NWD node. NVD = distance between a nest and the nearest vegetation, NWD = distance between a nest and water source. (G) Bayesian network model of cover type of Kentish plovers. We set the possibility of soil type at 100% of type A and type B. The BN models showed that the possibility of a successful nest being established in the node of nest fate was higher in cover type A model than in cover type B. NVD = distance between a nest and the nearest vegetation, NWD = distance between a nest and water source. (H) Bayesian network model of soil type of Kentish plovers. We set the possibility of soil type at 100% of type 1, type 2, and type 3. The BN models showed that the possibility of a successful nest being established in the node of nest fate was greater than 70% for types 2 and 3, not type 1. NVD = distance between a nest and the nearest vegetation, NWD = distance between a nest and water source. (I) Bayesian network model of nest fate of Kentish plovers in two locations. Depending on locations, NVD and soil type showed larger differences than other factors. NVD = distance between a nest and the nearest vegetation, NWD = distance between a nest and water source. **S2 Fig (A) Code of nest site selection model. (B) Code of cover type cluster analysis. (C) Code of soil type cluster analysis. (D) Code of nest fate model. (E) Code of nest failure PCA analysis**.
(ZIP)
