# Supplementary material for: Estimating nesting habitat characteristics for the Kentish plover (Anarhynchus alexandrinus) with the effect of substrate and vegetation using a Bayesian network approach
Source: PLoS One. 2025 Jun 11;20(6):e0325750. doi: 10.1371/journal.pone.0325750 (PMC12157252; doi:10.1371/journal.pone.0325750)
Supplement: S1 File — We set the possibility of nest site designation at 100% of the nest site. The < 20% category exhibited the highest possibility in the TV cover node and the 73–93% category, in the sand cover node. DV = dry vegetation, TV = total vegetation, LV = live vegetation, A = absence, P = presence. (B) Bayesian network model of non-nest site of Kentish plovers. We set the possibility of nest site designation at 100% of the non-nest site. The < 20% category exhibited the highest possibility in the TV cover node and the > 97% category, in the sand cover node. DV = dry vegetation, TV = total vegetation, LV = live vegetation, A = absence, P = presence. (C) Bayesian network model of sand cover of Kentish plovers. We set the possibility of sand cover at 100% of each category. The BN models demonstrated that the possibility of a nest site in the node of nest site designation decreased as the ambient sand cover increased. (D) Bayesian network model of total vegetation cover of Kentish plovers. We set the possibility of total vegetation cover at 100% of each category. The BN models exhibited that the possibility of a nest site in the node of nest site designation was higher when the ambient total vegetation cover was present to some extent. (E) Bayesian network model of nest site selection of Kentish plovers depending on locations. We set the possibility of each location at 100%. The BN models exhibited the local differences of vegetation and sand cover. (F) Bayesian network model of nest success and fail of Kentish plovers prepared. (A) We set the possibility of nest fate at 100% of nest success. The categories exhibiting the highest possibility of each node were type A (major type: sand) in the cover type node, type 3 (major type: very fine sand) in the sand type node, and the < 2 cm category in the NVD node. The 50 m and >230 m categories showed higher possibility than did the other categories in the NWD node. (B) We set the possibility of nest fate at 100% of nest failure. The cat [file pone.0325750.s001.zip › Supplementary_Kentish_plover_nest_modeling-1-final.docx]

**
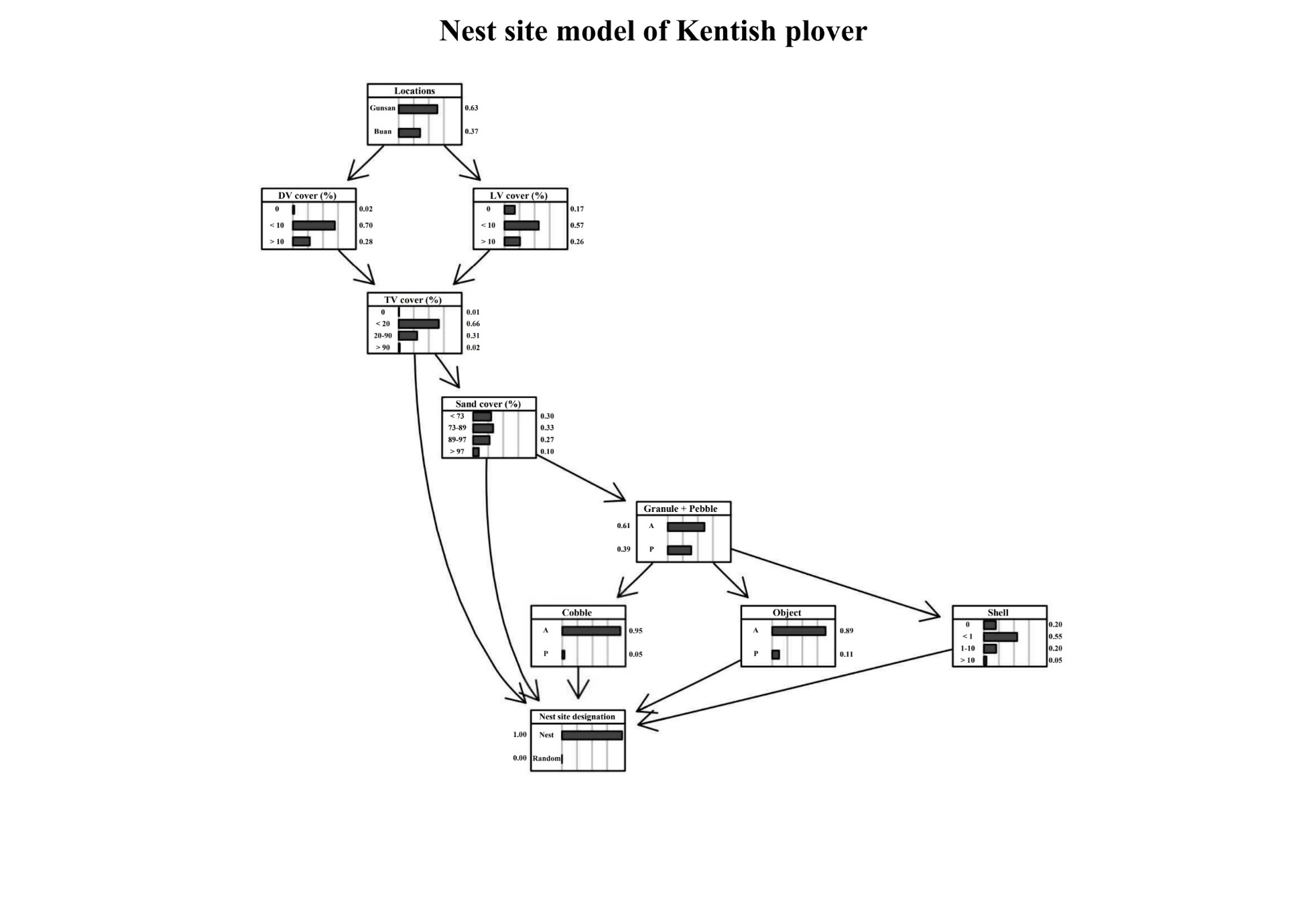
**Supplementary File S1: **Estimating nesting habitat characteristics for the Kentish Plover (*Anarhynchus alexandrinus*) with the effect of substrate and vegetation using a Bayesian network approach**

**Fig. A.** Bayesian network model of nest site of Kentish plovers. We set the possibility of nest site designation at 100% of the nest site. The <20% category exhibited the highest possibility in the TV cover node and the 73–93% category, in the sand cover node. DV = dry vegetation, TV = total vegetation, LV = live vegetation, A = absence, P = presence

**
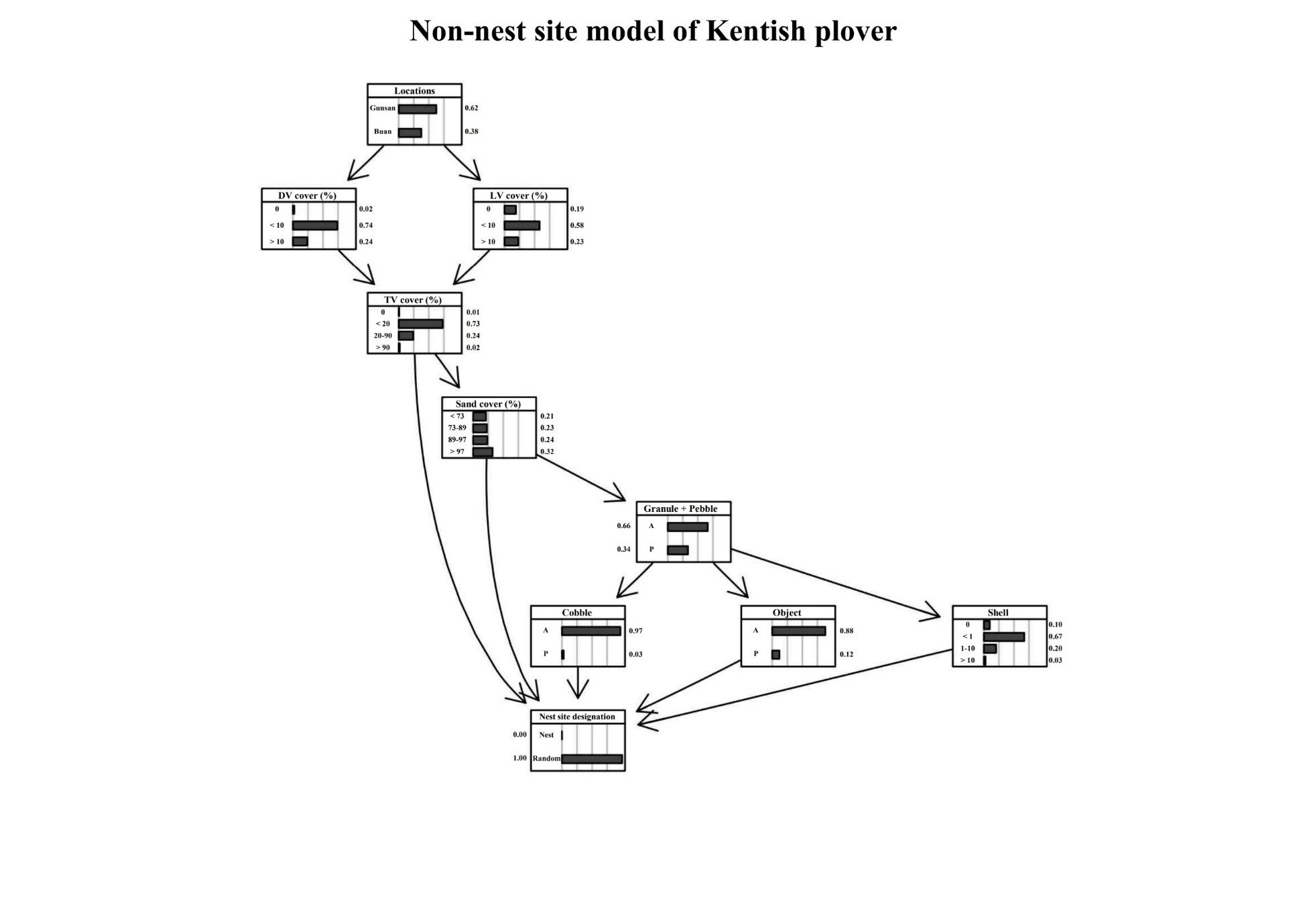

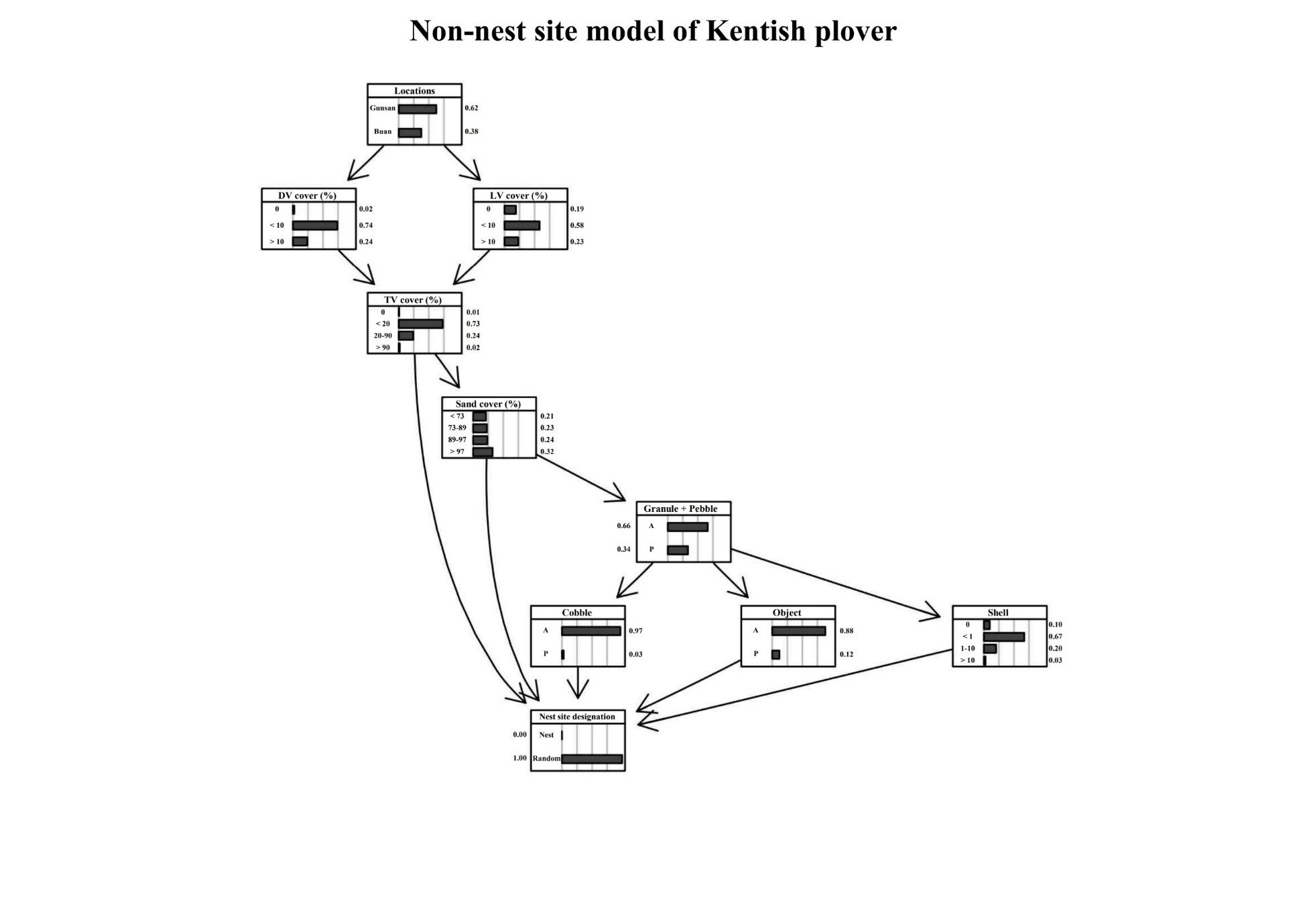
**

**Fig. B.** Bayesian network model of non-nest site of Kentish plovers. We set the possibility of nest site designation at 100% of the non-nest site. The <20% category exhibited the highest possibility in the TV cover node and the >97% category, in the sand cover node. DV = dry vegetation, TV = total vegetation, LV = live vegetation, A = absence, P = presence

| 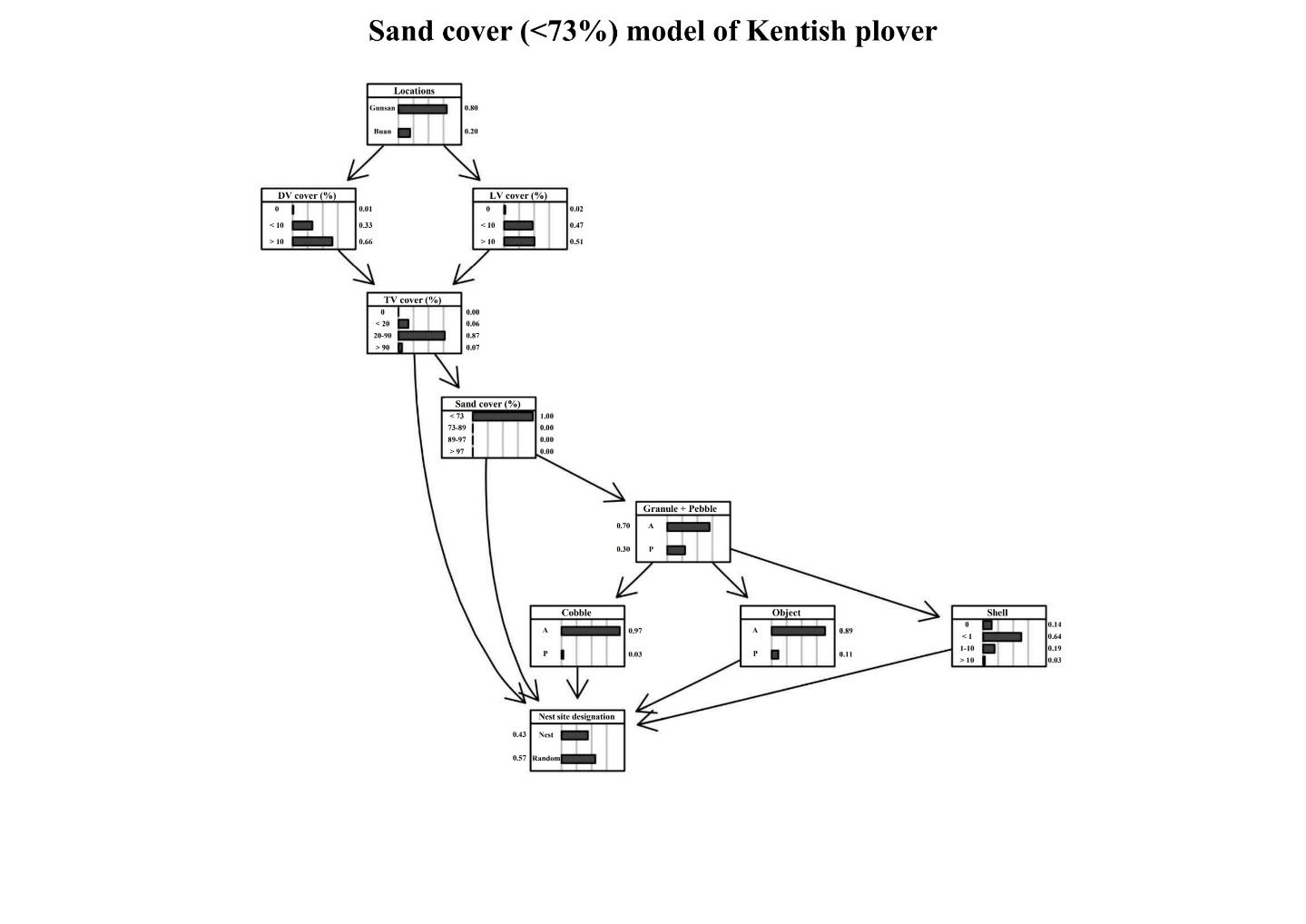 |
| --- |
| 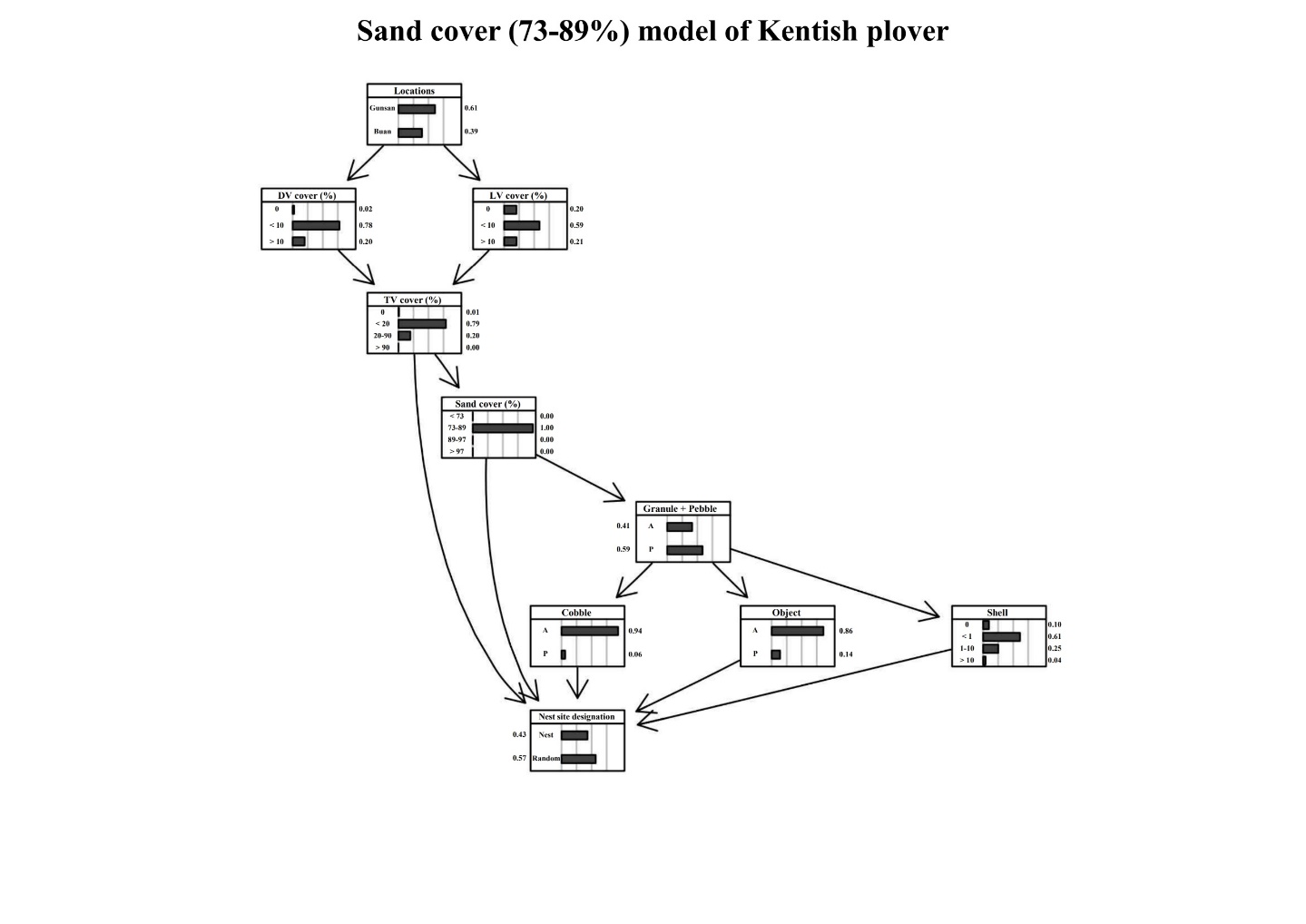 |

| 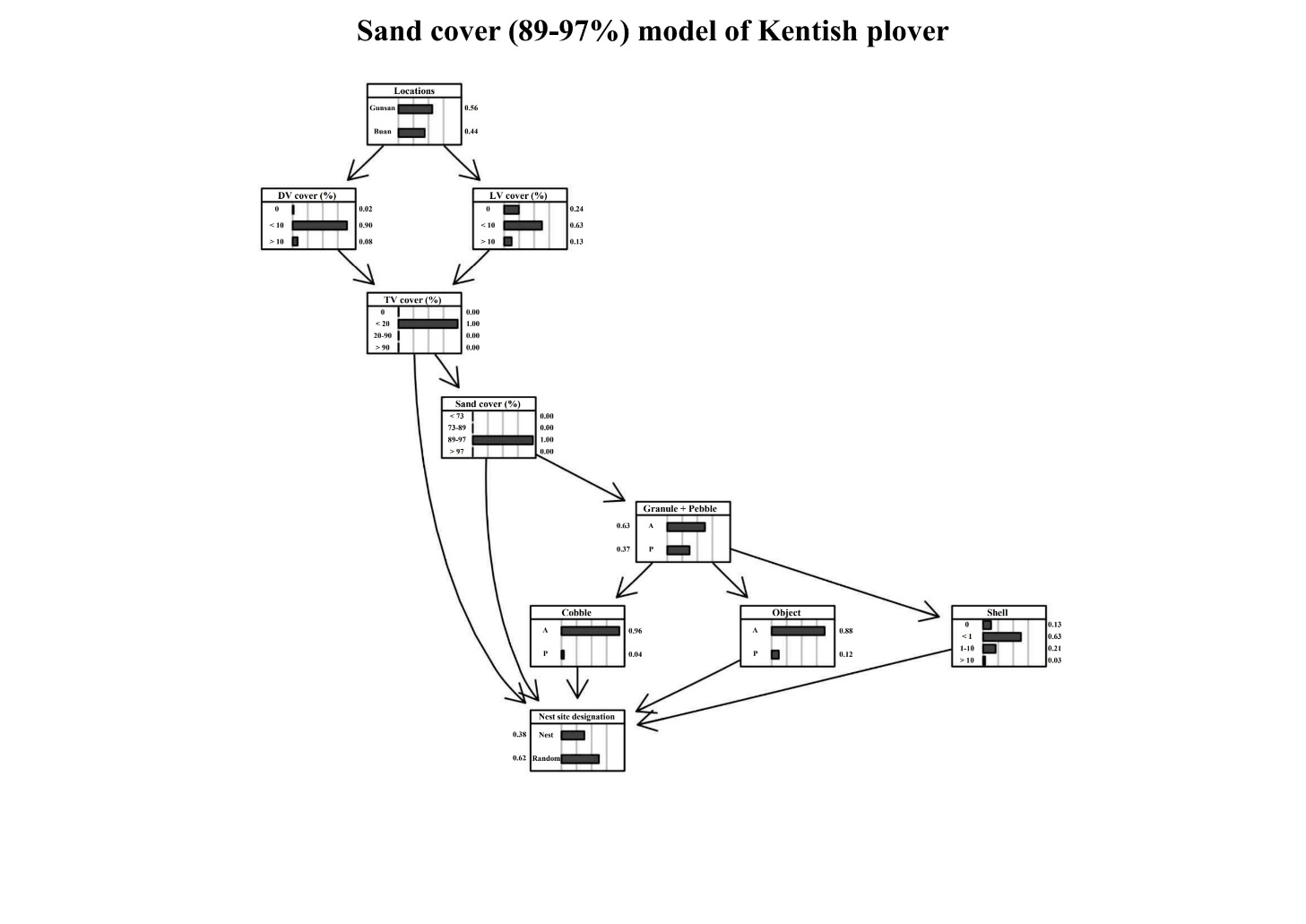 |
| --- |
| 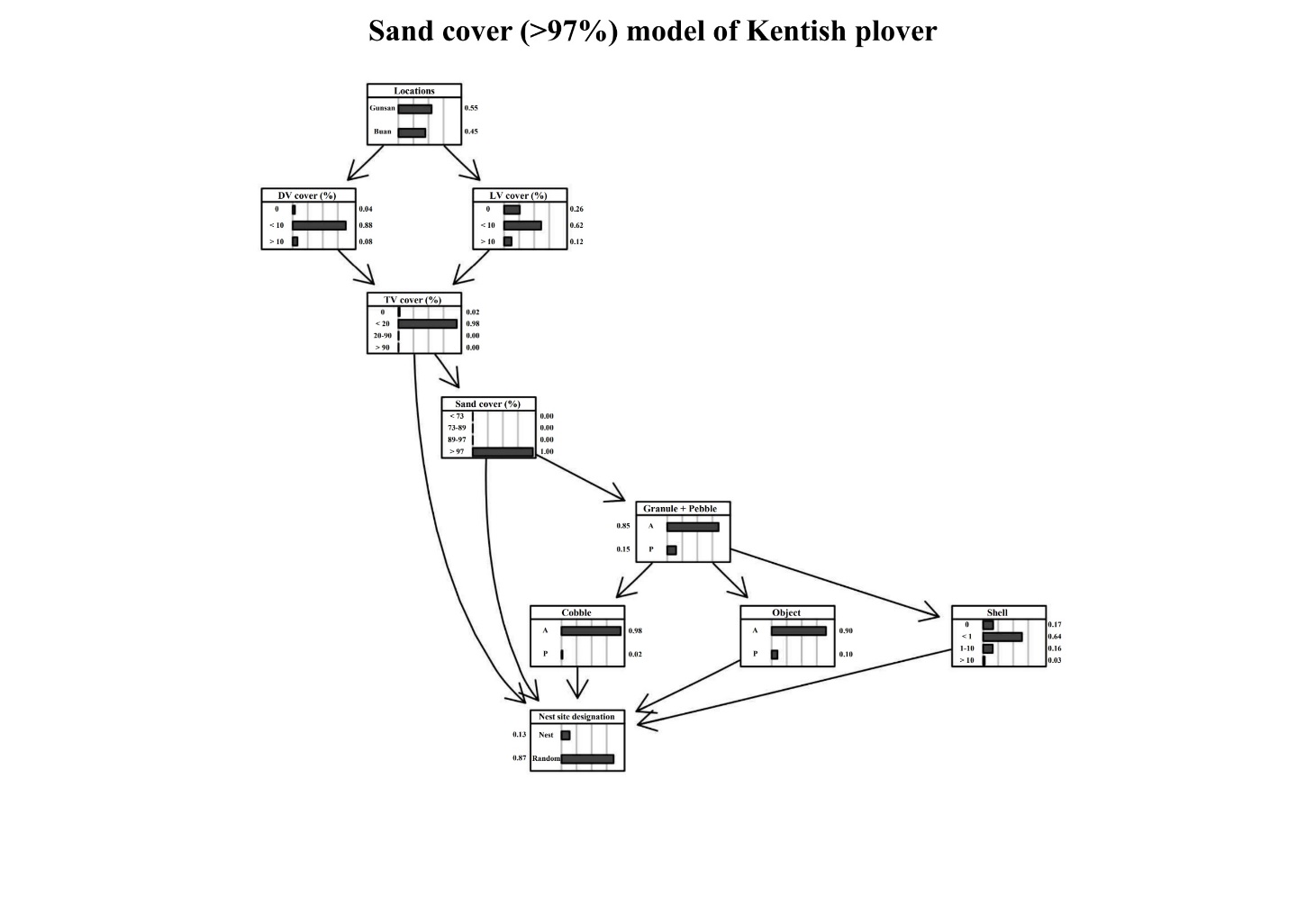 |

**Fig. C.** Bayesian network model of sand cover of Kentish plovers. We set the possibility of sand cover at 100% of each category. The BN models demonstrated that the possibility of a nest site in the node of nest site designation decreased as the ambient sand cover increased.

| 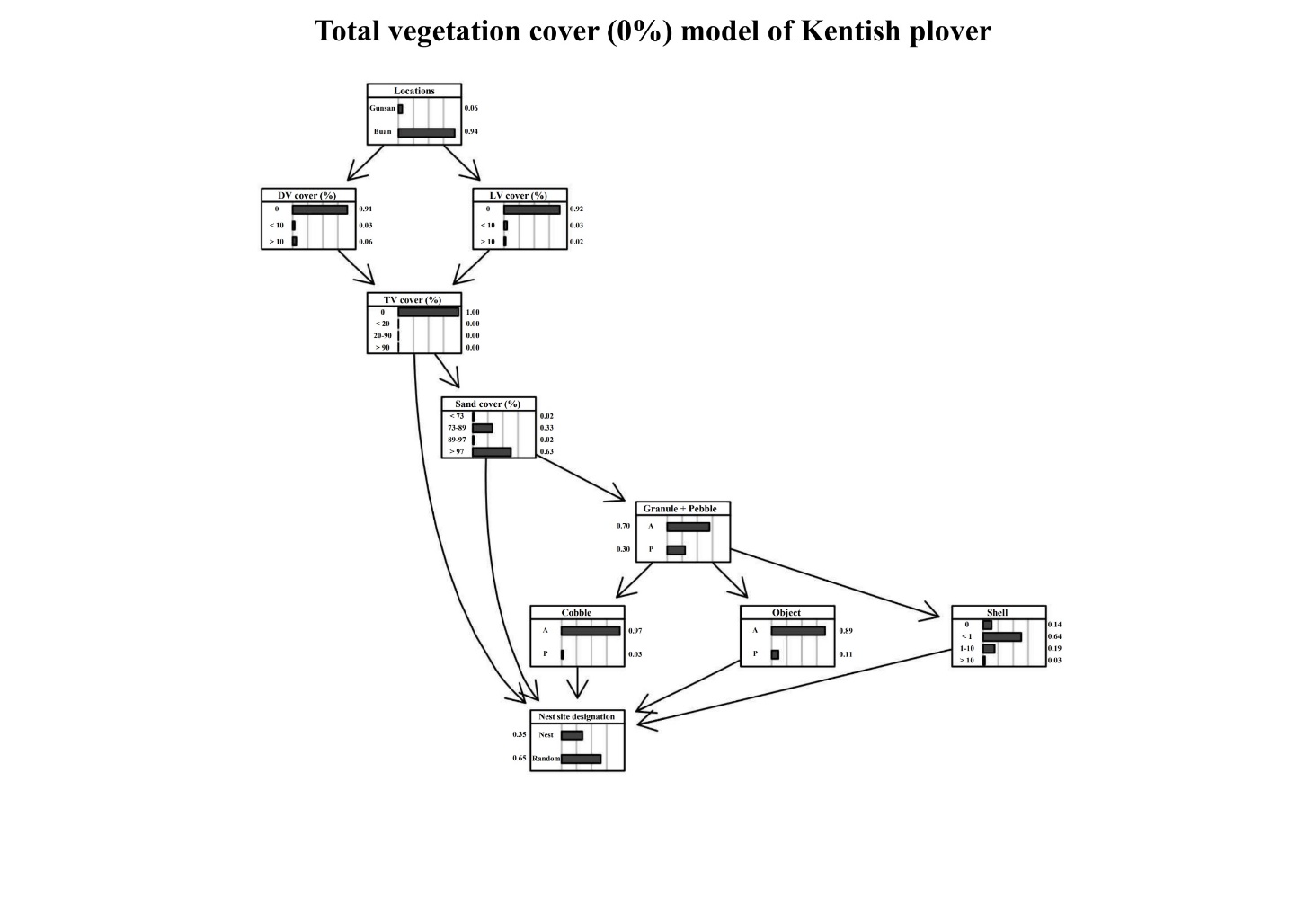 |
| --- |
| 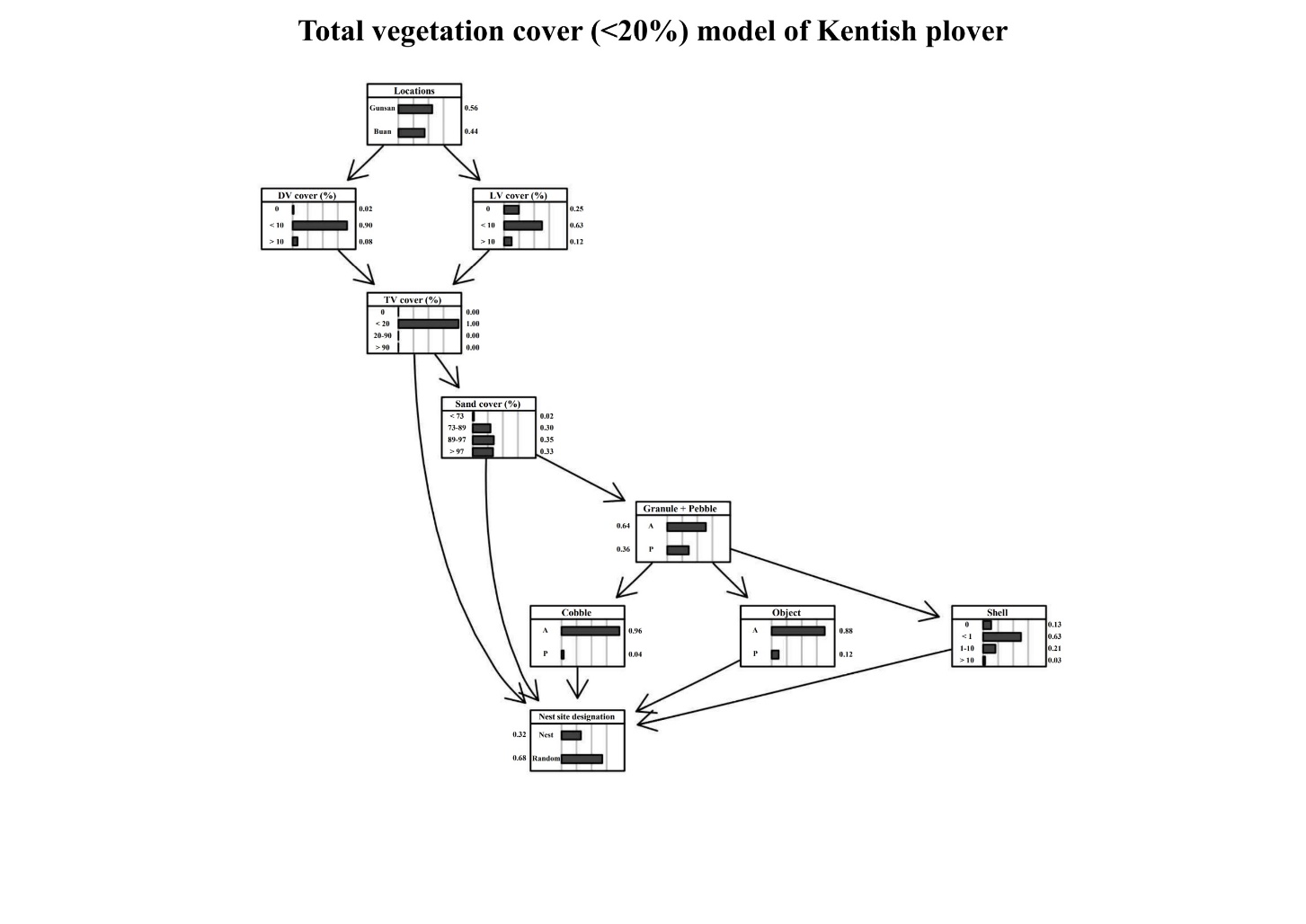 |

| 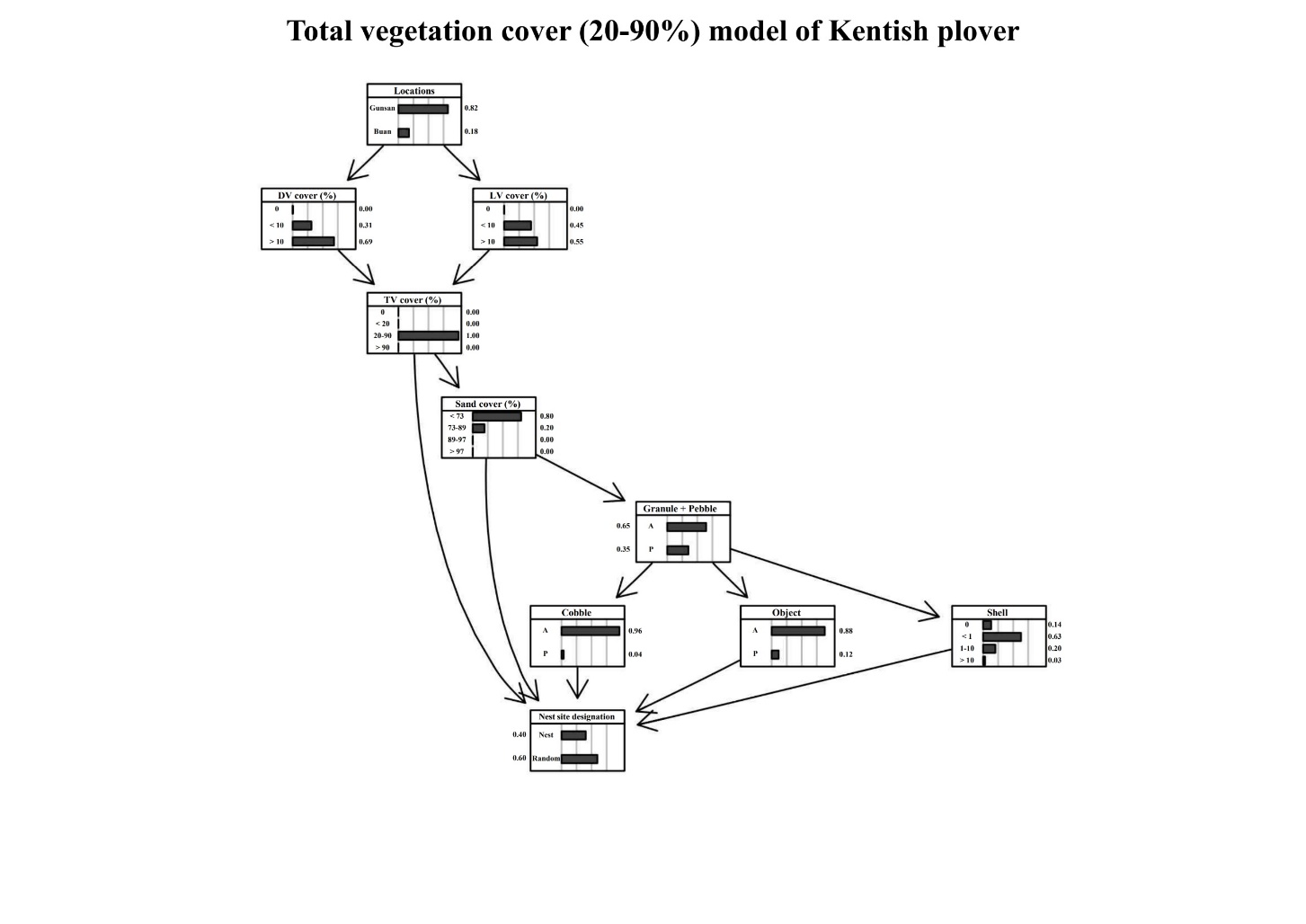 |
| --- |
| 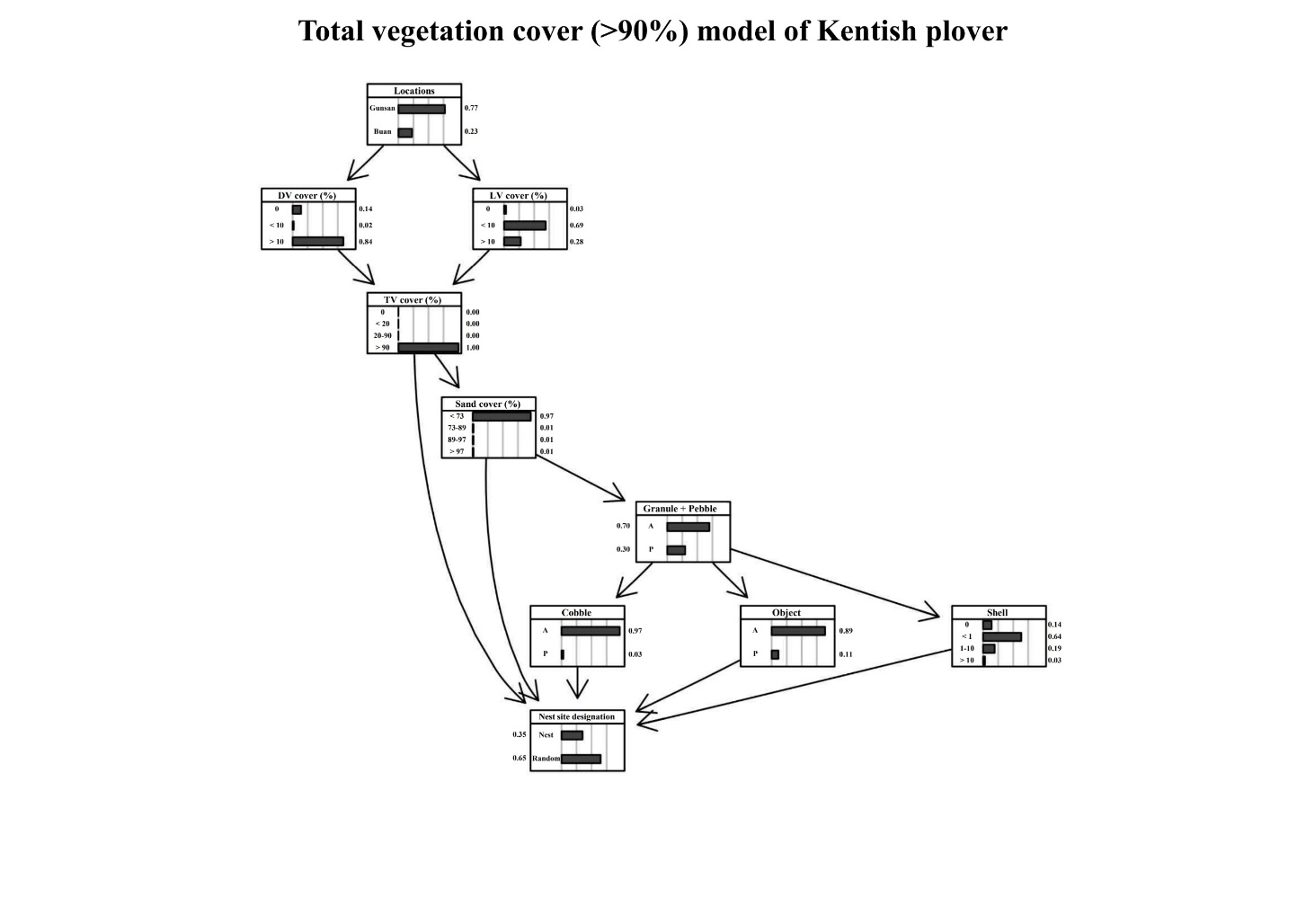 |

**Fig D.** Bayesian network model of total vegetation cover of Kentish plovers. We set the possibility of total vegetation cover at 100% of each category. The BN models exhibited that the possibility of a nest site in the node of nest site designation was higher when the ambient total vegetation cover was present to some extent.

| 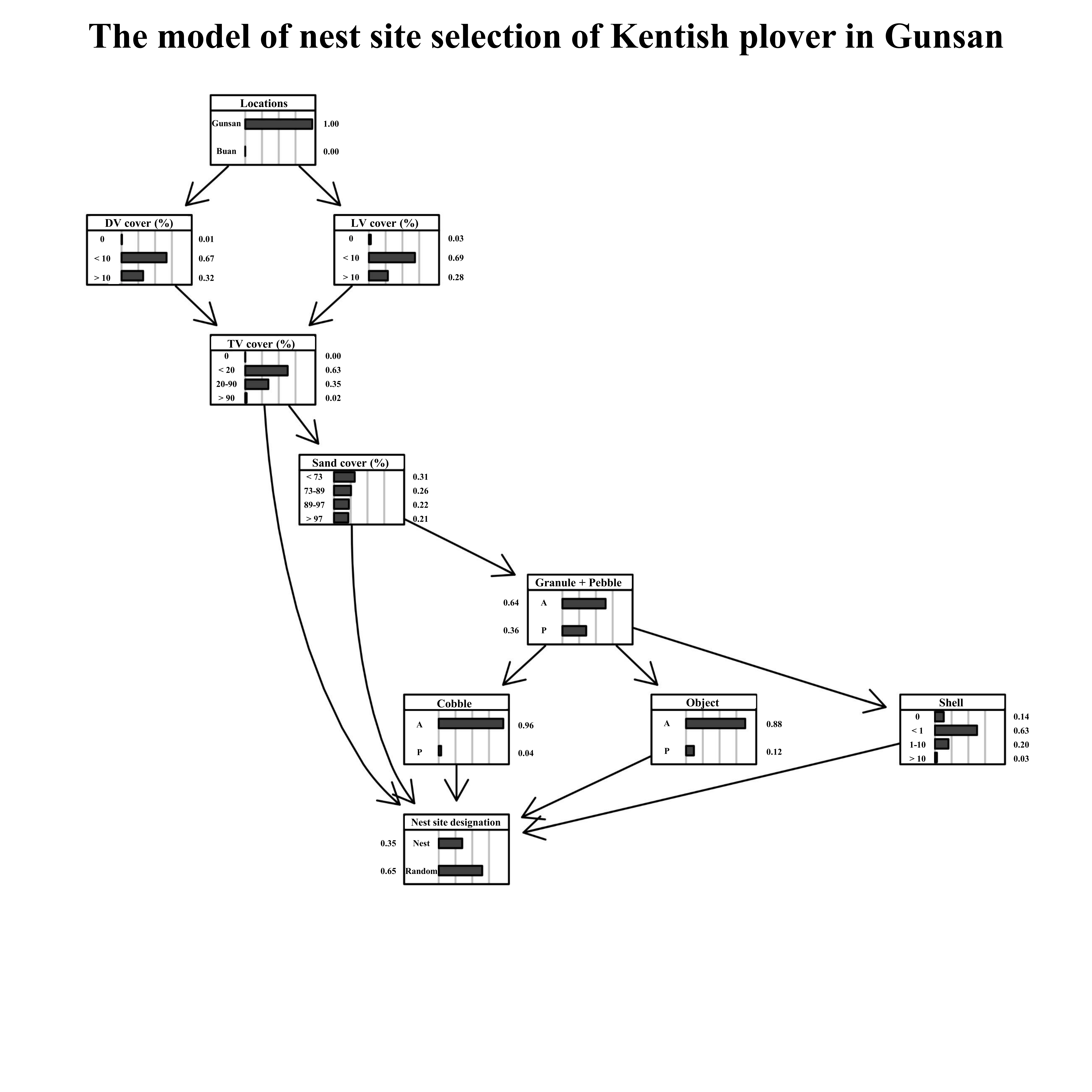 |
| --- |
| 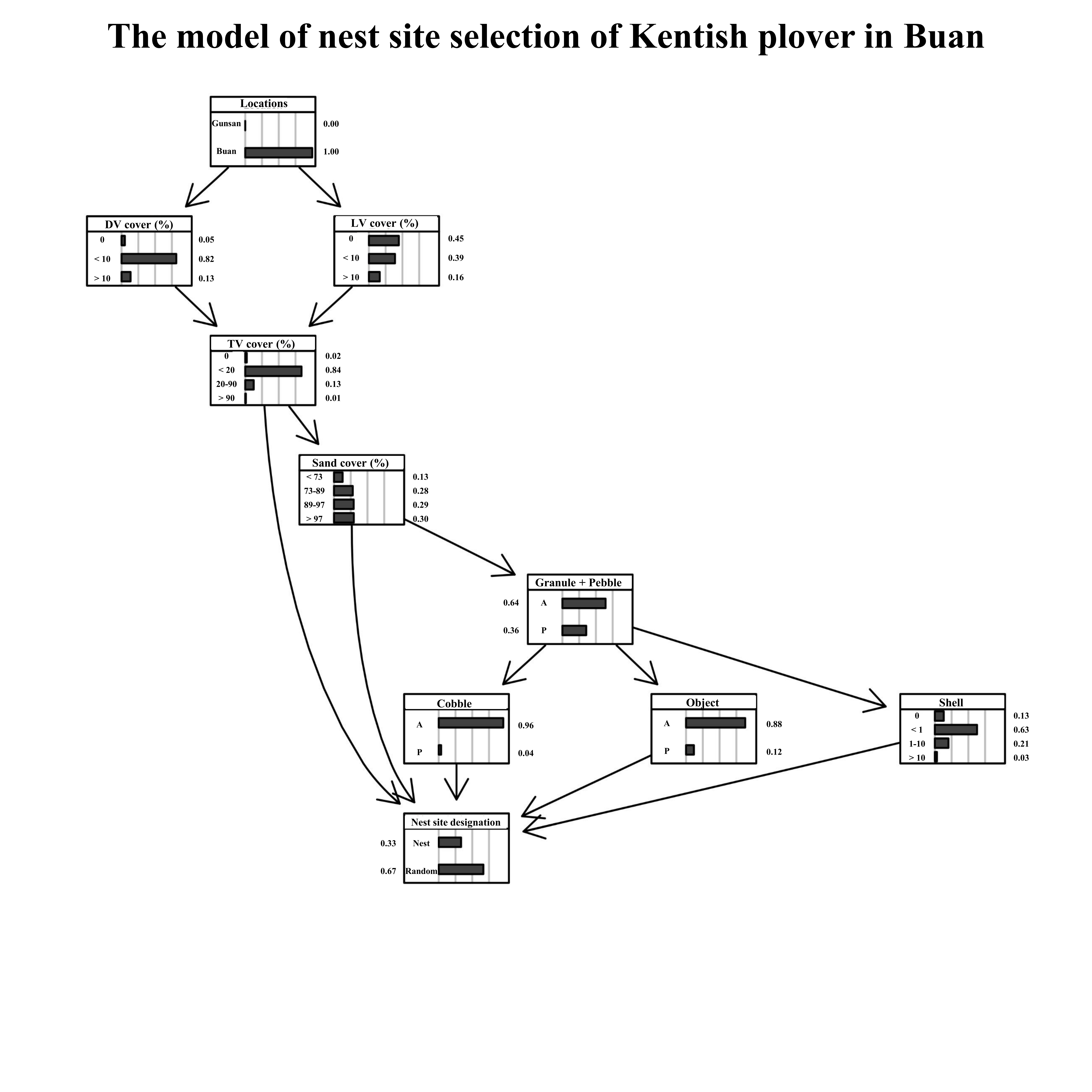 |

**Fig E.** Bayesian network model of nest site selection of Kentish plovers depending on locations. We set the possibility of each location at 100%. The BN models exhibited the local differences of vegetation and sand cover.

| **A)**  **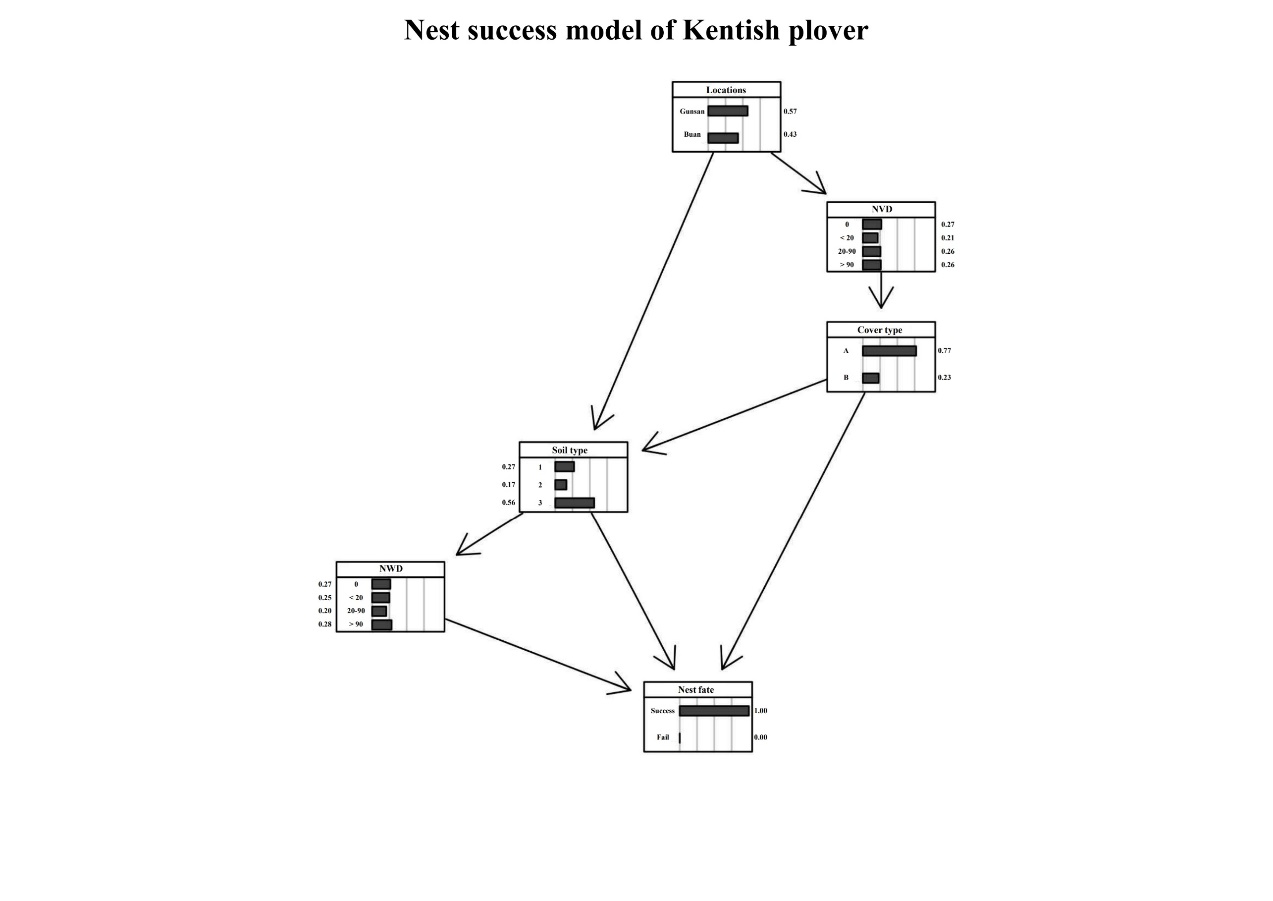** | **B)**  **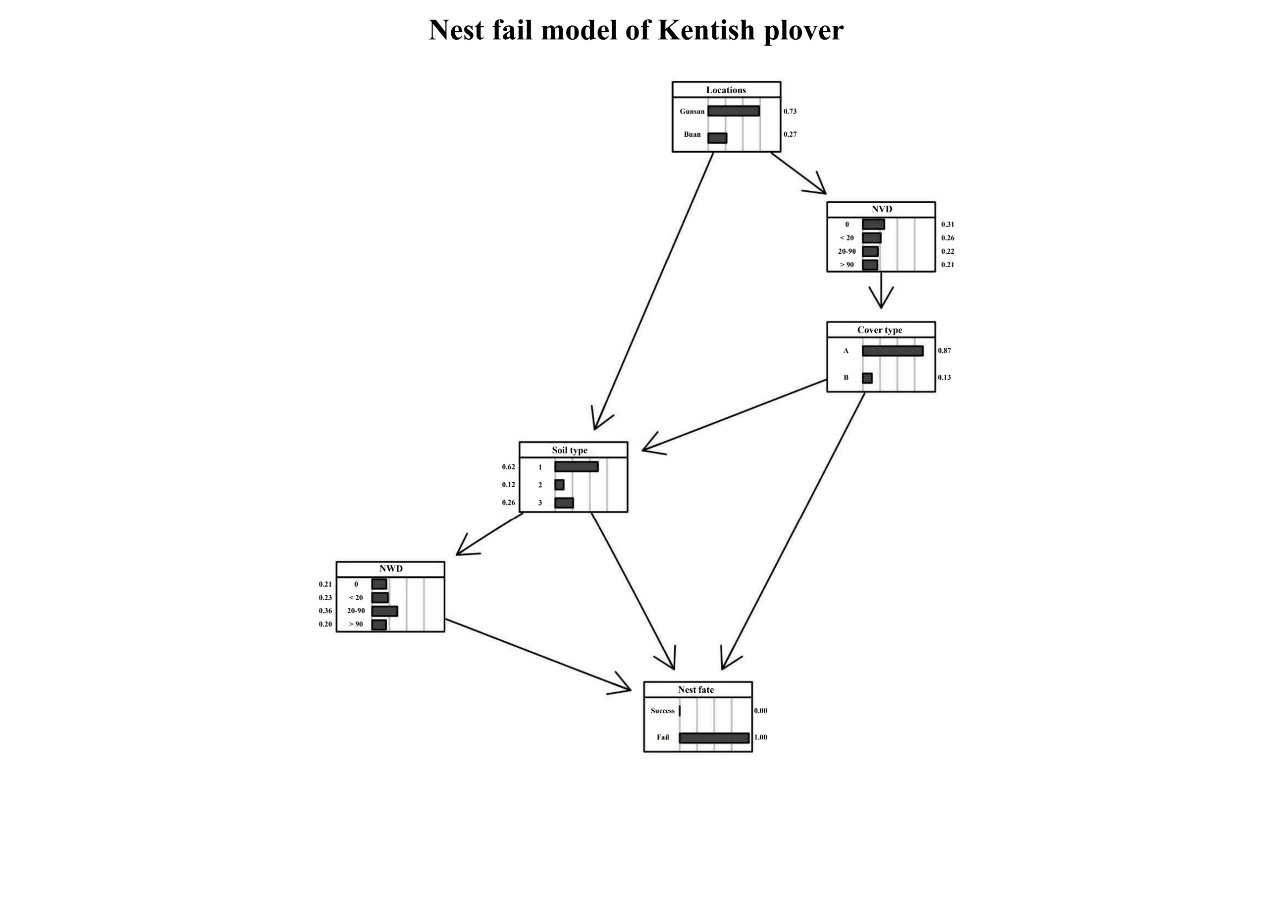** |
| --- | --- |

**Fig F.** Bayesian network model of nest success and fail of Kentish plovers prepared. (A) We set the possibility of nest fate at 100% of nest success. The categories exhibiting the highest possibility of each node were type A (major type: sand) in the cover type node, type 3 (major type: very fine sand) in the sand type node, and the <2 cm category in the NVD node. The 50 m and >230 m categories showed higher possibility than did the other categories in the NWD node. (B) We set the possibility of nest fate at 100% of nest failure. The categories exhibiting the highest possibility of each node were type A (major type: sand) in the cover type node, type 1 (major type: fine sand) in the sand type node, the <2 cm category in the NVD node, and the 140–230 m category in the NWD node. NVD = distance between a nest and the nearest vegetation, NWD = distance between a nest and water source

| **A)**  **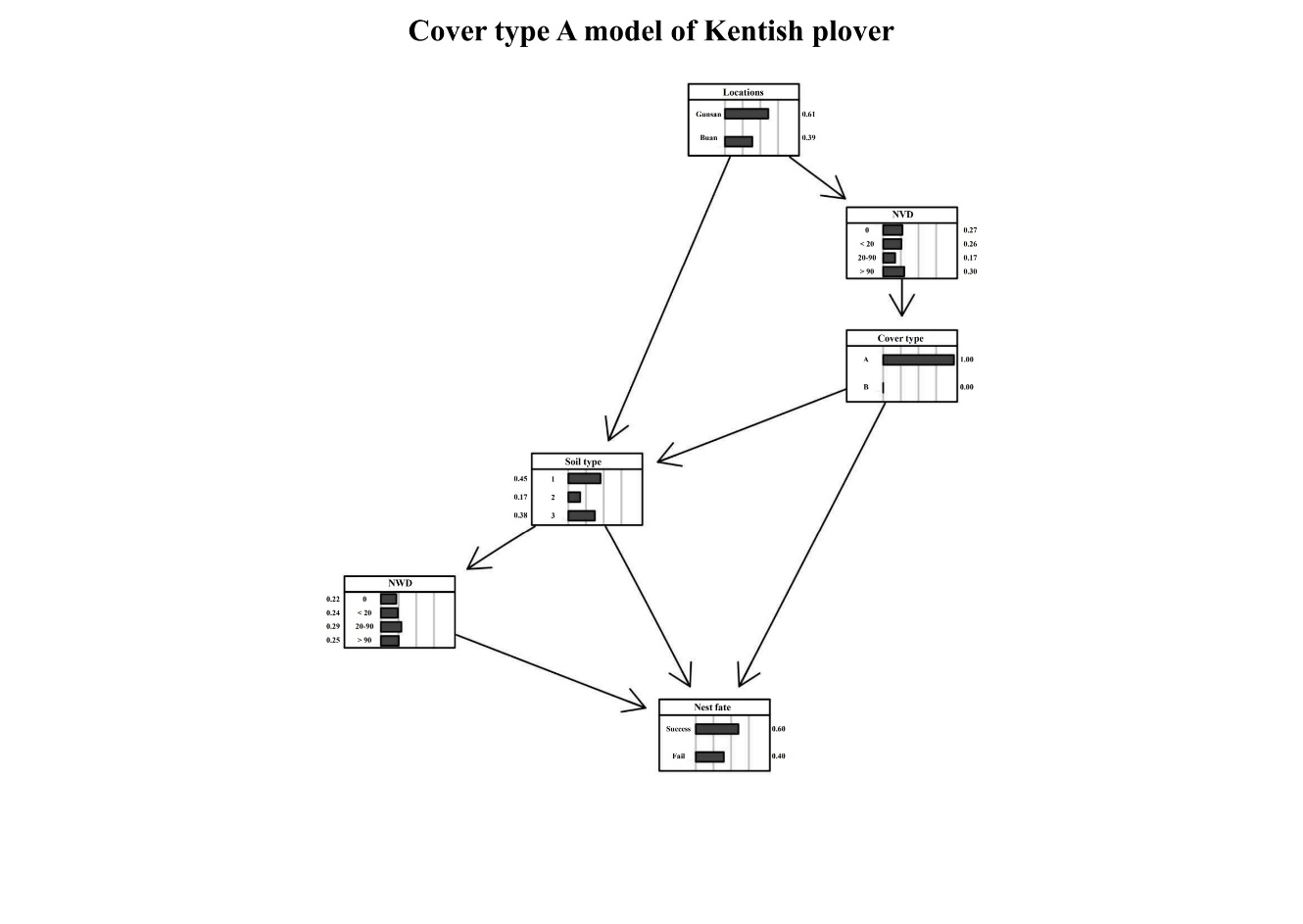** | **B)**  **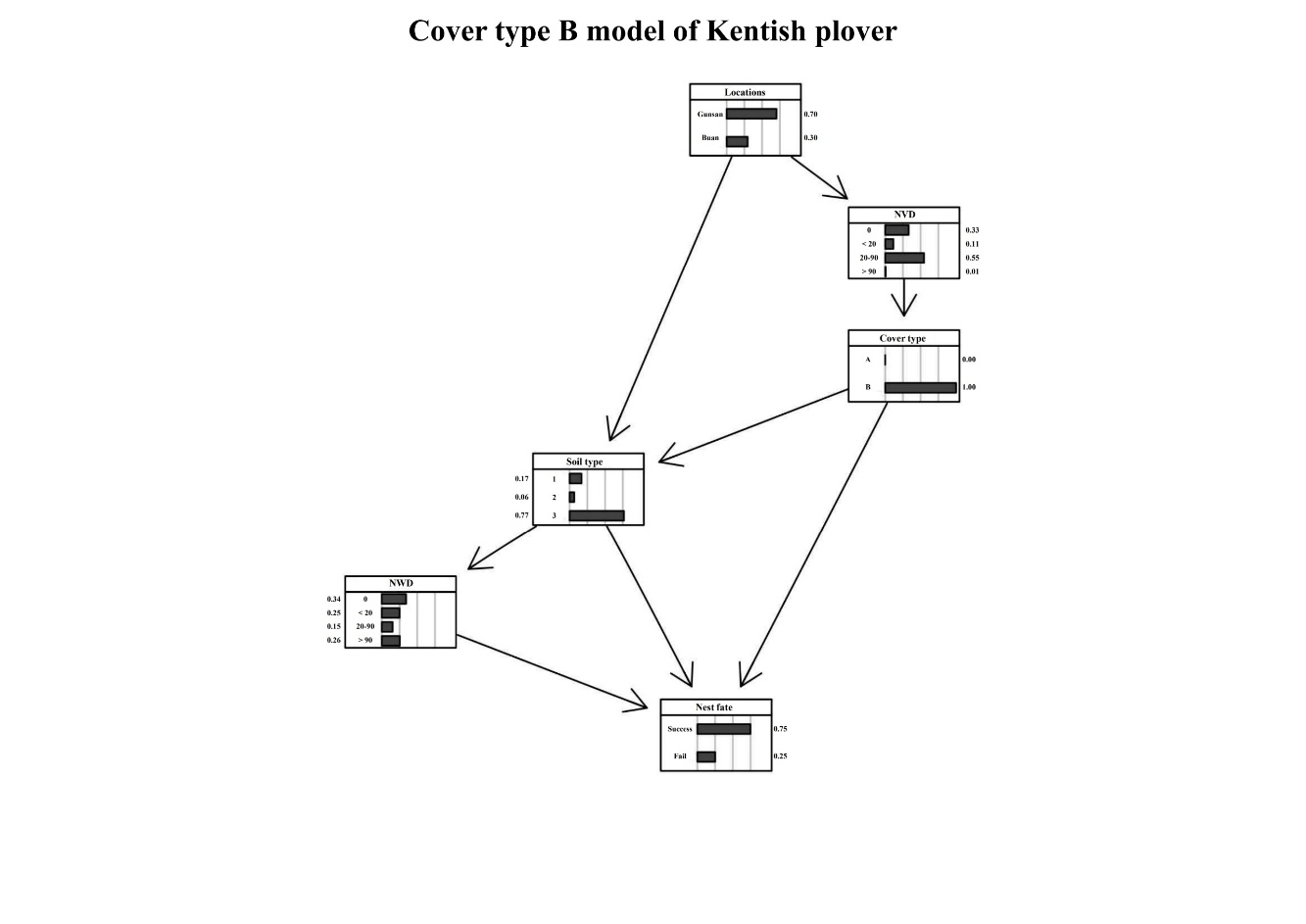** |
| --- | --- |

**Fig G.** Bayesian network model of cover type of Kentish plovers. We set the possibility of soil type at 100% of type A and type B. The BN models showed that the possibility of a successful nest being established in the node of nest fate was higher in cover type A model than in cover type B. NVD = distance between a nest and the nearest vegetation, NWD = distance between a nest and water source

| **A)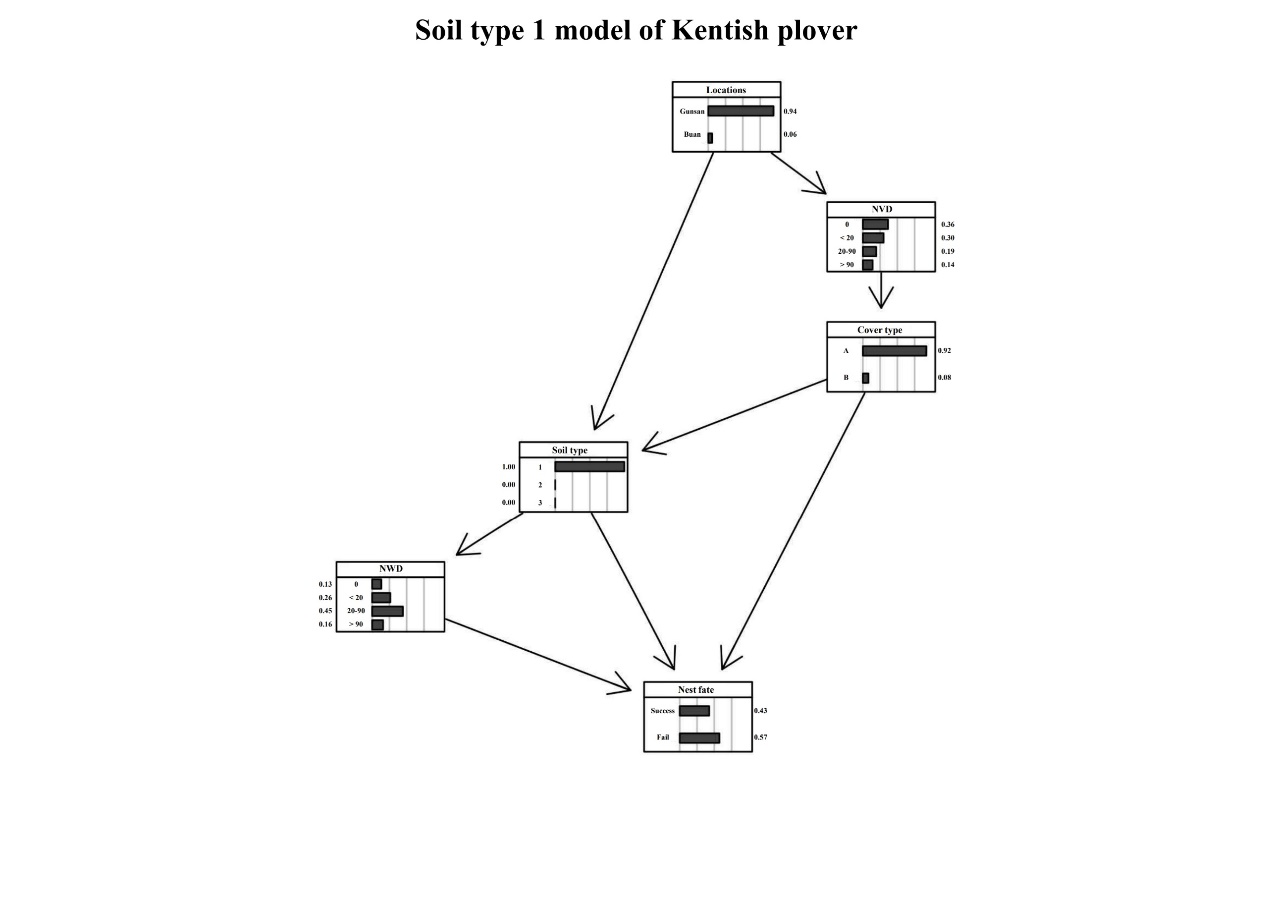** | **B)** **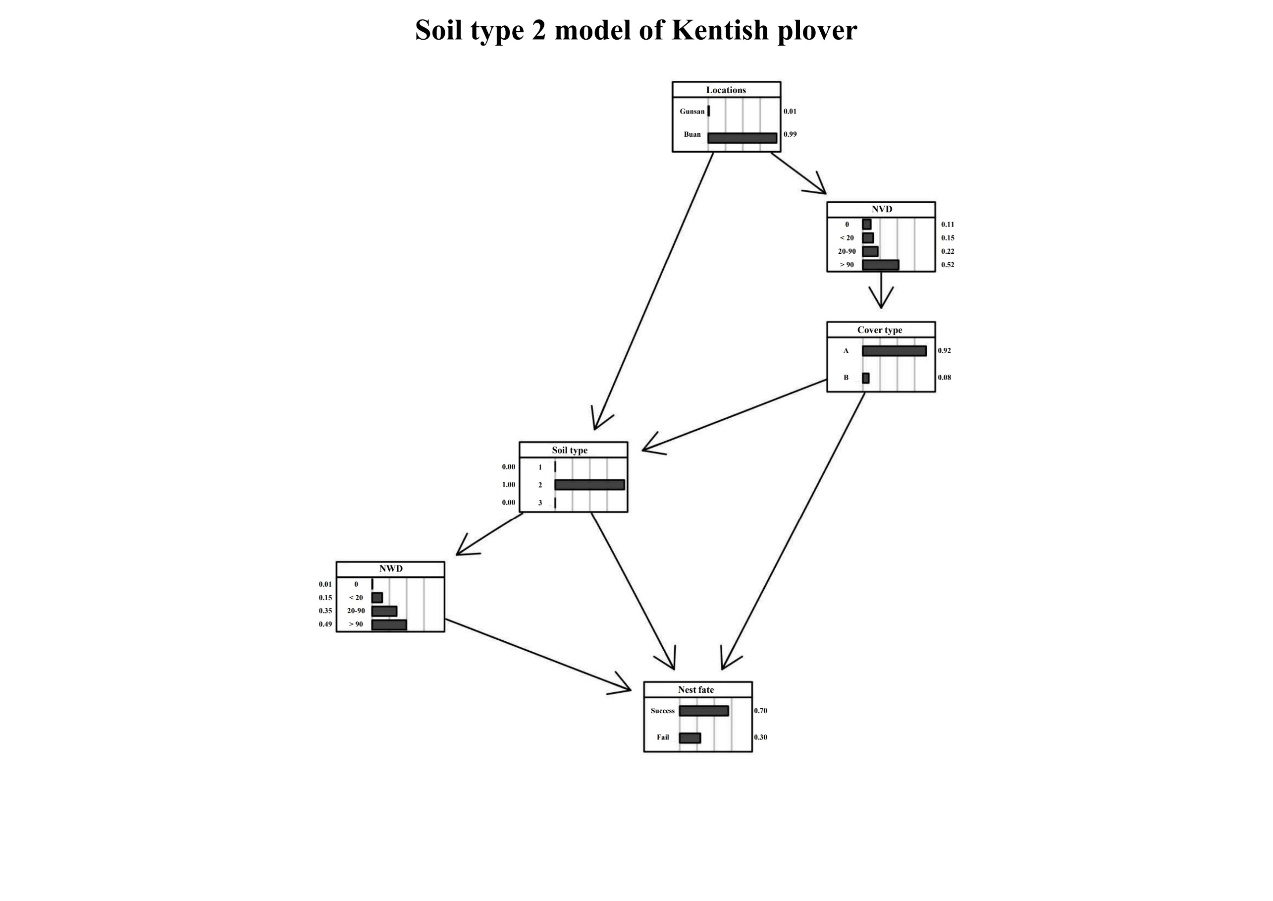** |
| --- | --- |
| **C)**  **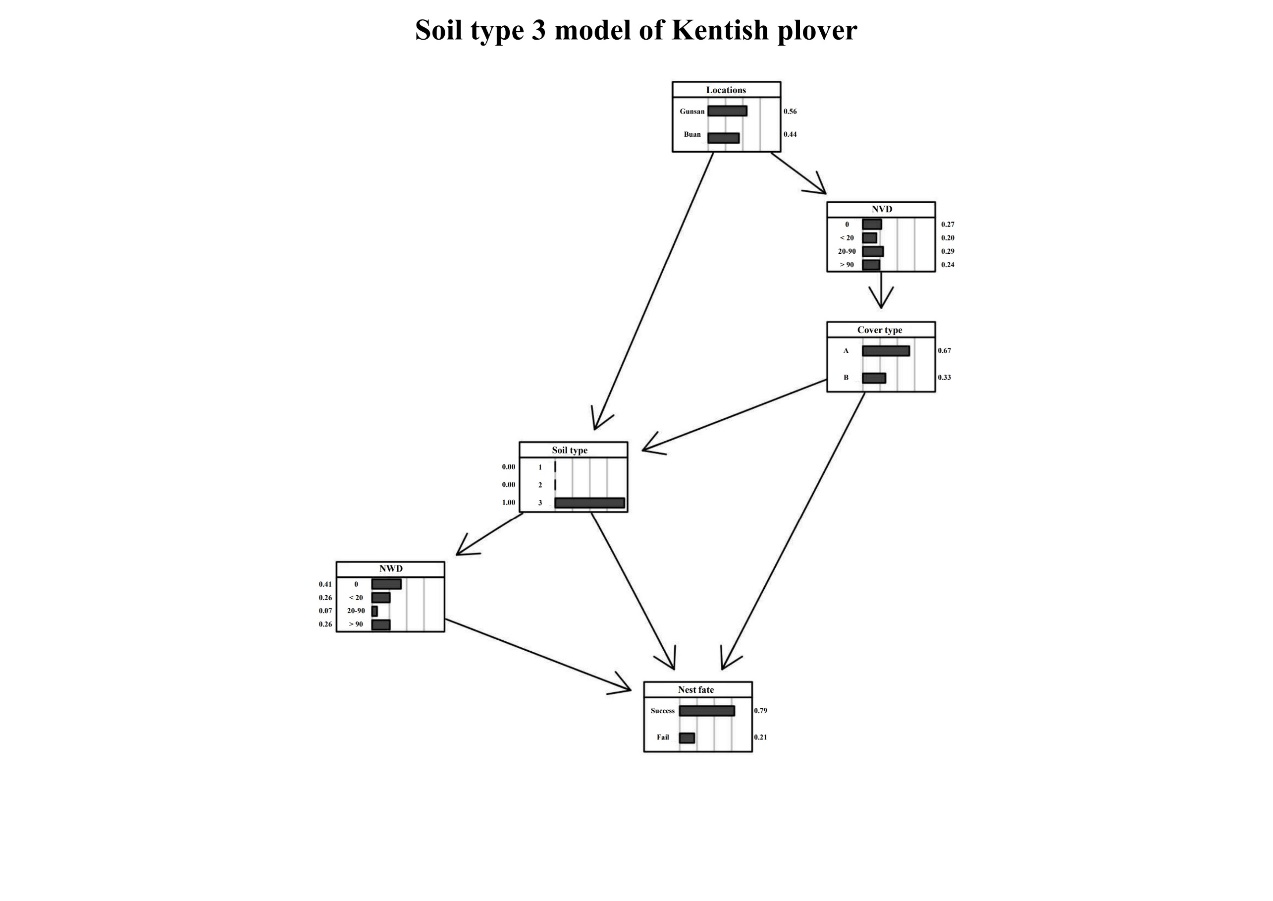** |  |

**Fig H.** Bayesian network model of soil type of Kentish plovers. We set the possibility of soil type at 100% of type 1, type 2, and type 3. The BN models showed that the possibility of a successful nest being established in the node of nest fate was greater than 70% for types 2 and 3, not type 1. NVD = distance between a nest and the nearest vegetation, NWD = distance between a nest and water source

| **A)** 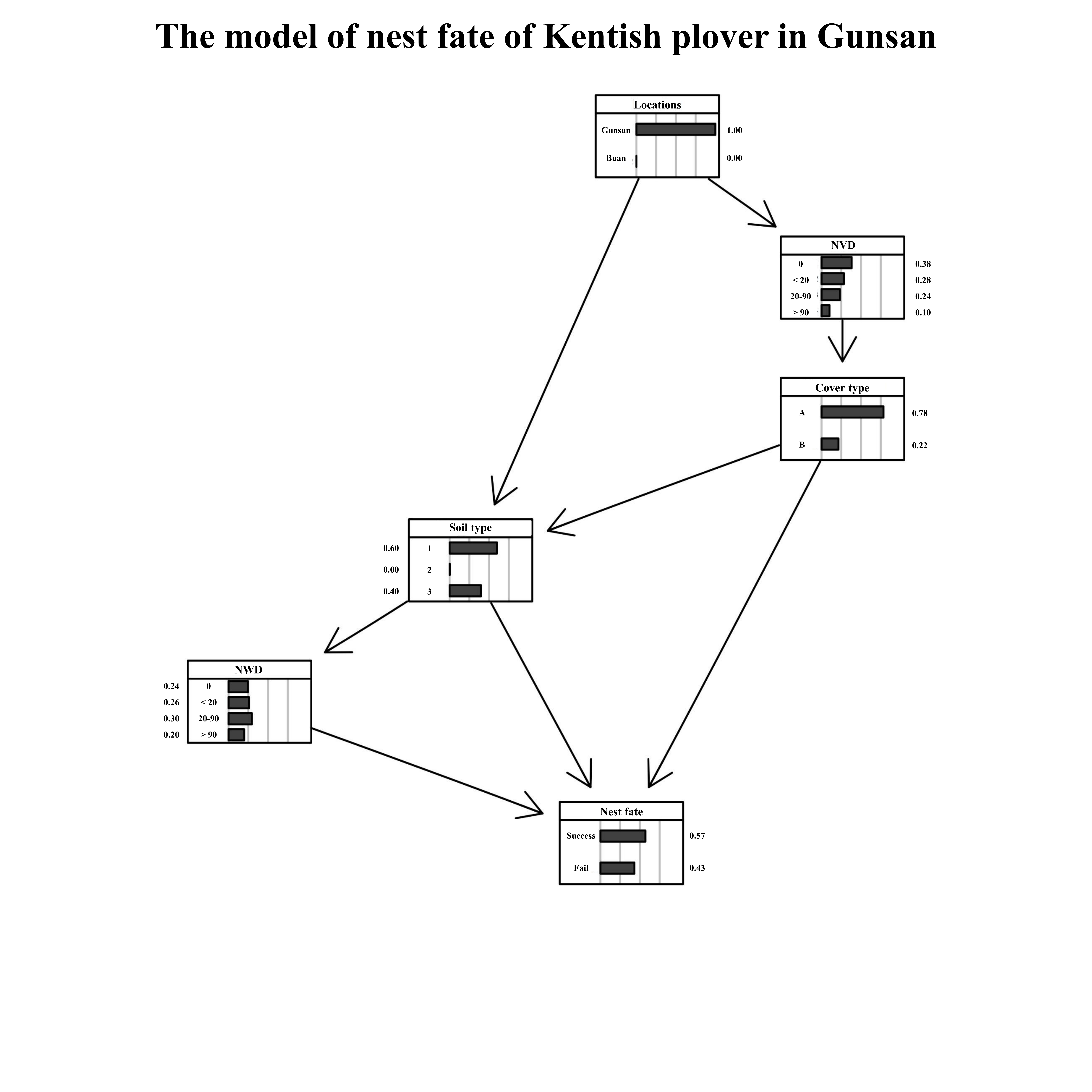 | **B)** 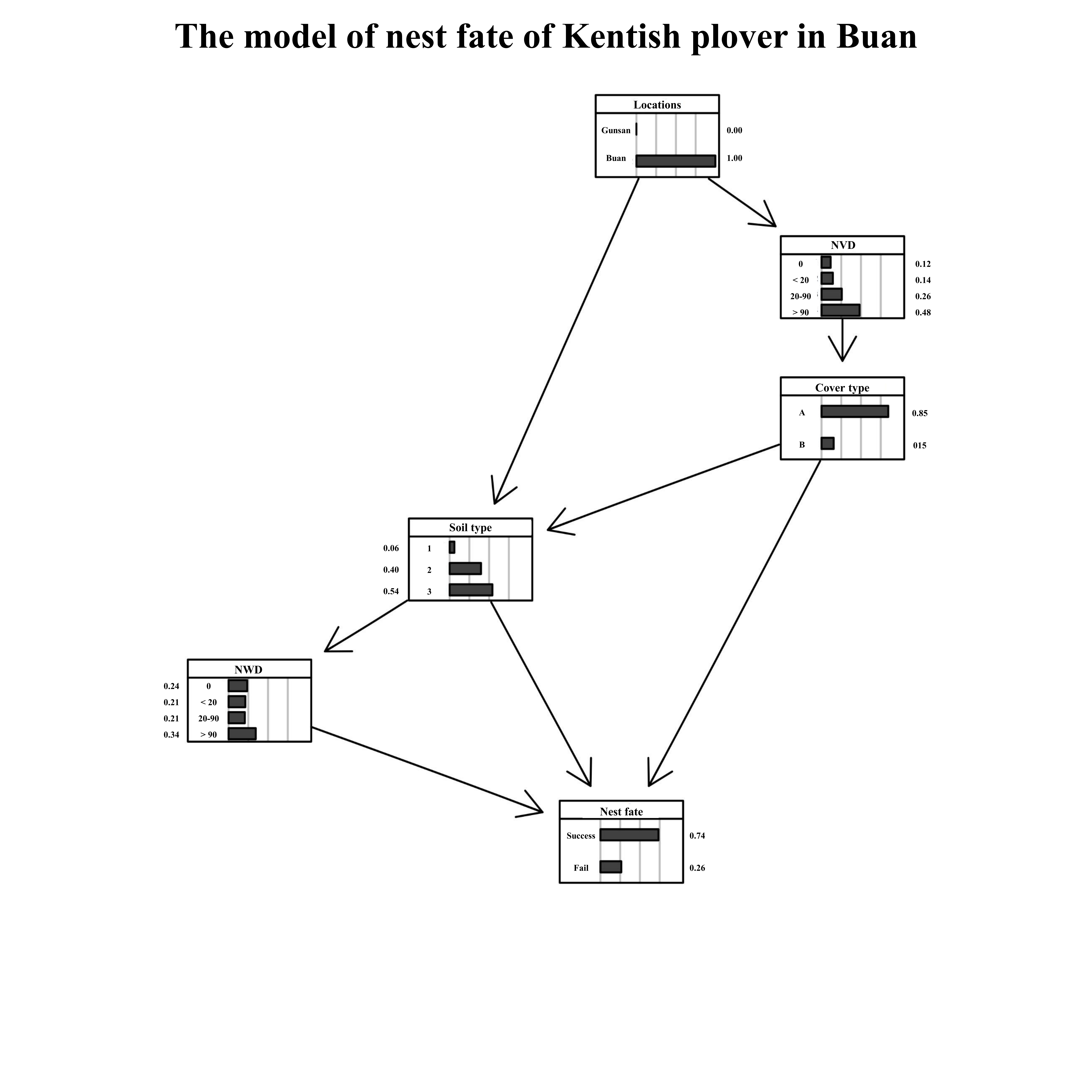 |
| --- | --- |

**Fig I.** Bayesian network model of nest fate of Kentish plovers in two locations. Depending on locations, NVD and soil type showed larger differences than other factors. NVD = distance between a nest and the nearest vegetation, NWD = distance between a nest and water source
