# Supplementary material for: Estimating nesting habitat characteristics for the Kentish plover (Anarhynchus alexandrinus) with the effect of substrate and vegetation using a Bayesian network approach
Source: PLoS One. 2025 Jun 11;20(6):e0325750. doi: 10.1371/journal.pone.0325750 (PMC12157252; doi:10.1371/journal.pone.0325750)
Supplement: S1 File — We set the possibility of nest site designation at 100% of the nest site. The < 20% category exhibited the highest possibility in the TV cover node and the 73–93% category, in the sand cover node. DV = dry vegetation, TV = total vegetation, LV = live vegetation, A = absence, P = presence. (B) Bayesian network model of non-nest site of Kentish plovers. We set the possibility of nest site designation at 100% of the non-nest site. The < 20% category exhibited the highest possibility in the TV cover node and the > 97% category, in the sand cover node. DV = dry vegetation, TV = total vegetation, LV = live vegetation, A = absence, P = presence. (C) Bayesian network model of sand cover of Kentish plovers. We set the possibility of sand cover at 100% of each category. The BN models demonstrated that the possibility of a nest site in the node of nest site designation decreased as the ambient sand cover increased. (D) Bayesian network model of total vegetation cover of Kentish plovers. We set the possibility of total vegetation cover at 100% of each category. The BN models exhibited that the possibility of a nest site in the node of nest site designation was higher when the ambient total vegetation cover was present to some extent. (E) Bayesian network model of nest site selection of Kentish plovers depending on locations. We set the possibility of each location at 100%. The BN models exhibited the local differences of vegetation and sand cover. (F) Bayesian network model of nest success and fail of Kentish plovers prepared. (A) We set the possibility of nest fate at 100% of nest success. The categories exhibiting the highest possibility of each node were type A (major type: sand) in the cover type node, type 3 (major type: very fine sand) in the sand type node, and the < 2 cm category in the NVD node. The 50 m and >230 m categories showed higher possibility than did the other categories in the NWD node. (B) We set the possibility of nest fate at 100% of nest failure. The cat [file pone.0325750.s001.zip › Supplementary_Kentish_plover_nest_modeling-2-final.docx]

Supplementary File S2: **Estimating nesting habitat characteristics for the Kentish Plover (*Anarhynchus alexandrinus*) with the effect of substrate and vegetation using a Bayesian network approach**

**Fig A.** Code of nest site selection model

# Load data

NRdata <- read.csv("File_1.csv", colClasses = "factor", header = TRUE)

# Load libraries

library(bnlearn)

library(deal)

library(Rgraphviz)

library(gRain)

library(ROCR)

library(Rmpfr)

library(RBGL)

# Hill-Climbing structure learning

NRhc = hc(NRdata)

plot(NRhc, main = "HC")

# Modify network: remove and add arcs

NRhc <- drop.arc(NRhc, from = "Location", to = "Live_vegetation")

NRhc <- drop.arc(NRhc, from = "Sand", to = "Live_vegetation")

NRhc <- set.arc(NRhc, from = "Total_vegetation", to = "Nest_designation")

NRhc <- set.arc(NRhc, from = "Live_vegetation", to = "Total_vegetation")

NRhc <- set.arc(NRhc, from = "Location", to = "Dry_vegetation")

NRhc <- set.arc(NRhc, from = "Location", to = "Live_vegetation")

NRhc <- set.arc(NRhc, from = "Cobble", to = "Nest_designation")

NRhc <- set.arc(NRhc, from = "Object", to = "Nest_designation")

NRhc <- set.arc(NRhc, from = "Shell", to = "Nest_designation")

# Plot and score

graphviz.plot(NRhc, main = "Modified HC")

score(NRhc, data = NRdata, type = "bic")

score(NRhc, data = NRdata, type = "bic", by.node = TRUE)

# Fit model

fitted = bn.fit(NRhc, data = NRdata, method = "bayes")

print(NRhc)

graphviz.plot(NRhc)

graphviz.chart(fitted, grid = TRUE, scale = c(0.7, 1.0), main = "Original model")

# Compile junction tree

NR.junction <- compile(as.grain(fitted))

# Set evidence for Nest site and Non-nest site

N100R0 <- setEvidence(NR.junction, nodes = "Nest_designation", states = "1")

graphviz.chart(as.bn.fit(N100R0, including.evidence = TRUE), grid = TRUE, scale = c(0.7, 1.0), main = "Nest site")

N0R100 <- setEvidence(NR.junction, nodes = "Nest_designation", states = "2")

graphviz.chart(as.bn.fit(N0R100, including.evidence = TRUE), grid = TRUE, scale = c(0.7, 1.0), main = "None-Nest site")

# Marginal queries

querygrain(NR.junction, nodes = c("Dry_vegetation","Live_vegetation","Total_vegetation","Sand", "Location",

"Granule.Pebble","Cobble","Object","Shell","Nest_designation"), type = "marginal")

# Evidence setting for Sand and Total Vegetation categories

for (i in 1:4) {

sand_evidence <- setEvidence(NR.junction, nodes = "Sand", states = as.character(i))

graphviz.chart(as.bn.fit(sand_evidence, including.evidence = TRUE), grid = TRUE, scale = c(0.7, 1.0), main = paste("Sand =", i))

querygrain(sand_evidence, nodes = c("Dry_vegetation","Live_vegetation","Total_vegetation","Sand", "Location",

"Granule.Pebble","Cobble","Object","Shell","Nest_designation"), type = "marginal")

}

for (i in 0:3) {

tv_evidence <- setEvidence(NR.junction, nodes = "Total_vegetation", states = as.character(i))

graphviz.chart(as.bn.fit(tv_evidence, including.evidence = TRUE), grid = TRUE, scale = c(0.7, 1.0), main = paste("Total Vegetation =", i))

querygrain(tv_evidence, nodes = c("Dry_vegetation","Live_vegetation","Total_vegetation","Sand", "Location",

"Granule.Pebble","Cobble","Object","Shell","Nest_designation"), type = "marginal")

}

# Location-specific evidence

Location1 <- setEvidence(NR.junction, nodes = "Location", states = "1")

graphviz.chart(as.bn.fit(Location1, including.evidence = TRUE), grid = TRUE, scale = c(0.7, 1.0), main = "Gunsan")

Location2 <- setEvidence(NR.junction, nodes = "Location", states = "2")

graphviz.chart(as.bn.fit(Location2, including.evidence = TRUE), grid = TRUE, scale = c(0.7, 1.0), main = "Buan")

# Arc strengths and model performance

arc.strength(NRhc, data = NRdata, criterion = "x2")

arc.strength(NRhc, data = NRdata, criterion = "bic")

NR.strength <- bf.strength(NRhc, NRdata, score = "bde", prior = "marginal")

# Prediction evaluation

NR.fitted <- bn.fit(NRhc, NRdata)

predictions <- predict(NR.fitted, node = "Nest_designation", data = NRdata)

actual <- NRdata$Nest_designation

table(Predicted = predictions, Actual = actual)

# ROC and AUC

predictions_numeric <- as.numeric(as.factor(predictions))

actual_numeric <- as.numeric(as.factor(actual))

roc <- prediction(predictions_numeric, actual_numeric)

perf <- performance(roc, "tpr", "fpr")

plot(perf, main = "ROC Curve")

auc <- performance(roc, measure = "auc")

auc_value <- auc@y.values[[1]]

print(paste("AUC Value:", auc_value))

# Cross-validation

bn.cv(NRdata, 'hc', loss = "logl", method = "hold-out", k = 10, m = 10)

bn.cv(NRdata, 'hc', loss = "pred", loss.args = list(target = "Nest_designation"))

**Fig B.** Code of cover type cluster analysis

# Load data

quad_data <- read.csv("File_2.csv")

# Load necessary libraries

library(factoextra)

library(dplyr)

# Set seed for reproducibility

set.seed(2020)

# Determine the optimal number of clusters using Elbow and Silhouette methods

fviz_nbclust(quad_data[,4:ncol(quad_data)], kmeans, method = "wss", k.max = 15) +

theme_minimal() + ggtitle("Elbow Method")

fviz_nbclust(quad_data[,4:ncol(quad_data)], kmeans, method = "silhouette", k.max = 15) +

theme_minimal() + ggtitle("Silhouette Method")

# Perform K-means clustering with 2 centers

df.quadrat <- kmeans(quad_data[,4:ncol(quad_data)], center = 2, iter.max = 1000)

df.quadrat

# Visualize the cluster centers

barplot(t(df.quadrat$centers), beside=TRUE, col = 1:7)

legend("topleft", colnames(quad_data[,4:10]), fill = 1:7, cex = 0.5)

# Append cluster assignment to data and export

quad_data$cluster <- df.quadrat$cluster

write.csv(quad_data, file = "Nest_fate_quadratdata1.csv")

**Fig C.** Code of soil type cluster analysis

# Load data

soildata <- read.csv("File_3.csv")

# Load required libraries

library(factoextra)

library(dplyr)

# Set seed for reproducibility

set.seed(2020)

# Determine the optimal number of clusters using Elbow and Silhouette methods

fviz_nbclust(soildata[,4:ncol(soildata)], kmeans, method = "wss", k.max = 15) +

theme_minimal() +

ggtitle("Elbow Method")

fviz_nbclust(soildata[,4:ncol(soildata)], kmeans, method = "silhouette", k.max = 15) +

theme_minimal() +

ggtitle("Silhouette Method")

# Apply K-means clustering with k = 3

df.soil <- kmeans(soildata[,4:ncol(soildata)], center = 3, iter.max = 1000)

df.soil

# Visualize cluster centers

barplot(t(df.soil$centers), beside=TRUE, col = 1:7)

legend("topleft", colnames(soildata[,4:10]), fill = 1:7, cex = 0.5)

# Add cluster assignments to original data and export

soildata$cluster <- df.soil$cluster

write.csv(soildata, file = "soildata_cluster.csv")

**Fig D.** Code of nest fate model

# Load data

NFdata <- read.csv("File_4.csv", colClasses = "factor", header = TRUE)

# Load required libraries

library(bnlearn)

library(deal)

library(Rgraphviz)

library(gRain)

library(ROCR)

library(Rmpfr)

library(RBGL)

# Structure learning using Hill-Climbing

NFhc = hc(NFdata)

plot(NFhc, main = "HC")

# Modify arcs

NFhc <- drop.arc(NFhc, from = "Location", to = "Soil_cluster")

NFhc <- set.arc(NFhc, from = "Cover_cluster", to = "nest_fate")

NFhc <- set.arc(NFhc, from = "Location", to = "Soil_cluster")

NFhc <- set.arc(NFhc, from = "Cover_cluster", to = "Soil_cluster")

NFhc <- set.arc(NFhc, from = "NWD", to = "nest_fate")

# Visualize modified structure

graphviz.plot(NFhc, main = "Modified HC")

# BIC score assessment

score(NFhc, data = NFdata, type = "bic")

score(NFhc, data = NFdata, type = "bic", by.node = TRUE)

# Fitting parameters

fitted_model <- bn.fit(NFhc, data = NFdata, method = "bayes")

graphviz.chart(fitted_model, grid = TRUE, scale = c(0.7, 1.0), main = "Original model")

# Compile into junction tree for inference

NF.junction <- compile(as.grain(fitted_model))

# Inference examples

graphviz.chart(as.bn.fit(setEvidence(NF.junction, nodes = "nest_fate", states = "1"), including.evidence = TRUE), grid = TRUE, scale = c(0.7, 1.0), main = "Nest success")

graphviz.chart(as.bn.fit(setEvidence(NF.junction, nodes = "nest_fate", states = "2"), including.evidence = TRUE), grid = TRUE, scale = c(0.7, 1.0), main = "Nest fail")

# Set evidence by soil cluster

for (i in 1:3) {

state <- paste(i)

evidence <- setEvidence(NF.junction, nodes = "Soil_cluster", states = state)

graphviz.chart(as.bn.fit(evidence, including.evidence = TRUE), grid = TRUE, scale = c(0.7, 1.0), main = paste("Soil", state))

}

# Set evidence by cover cluster

for (i in 1:2) {

state <- paste(i)

evidence <- setEvidence(NF.junction, nodes = "Cover_cluster", states = state)

graphviz.chart(as.bn.fit(evidence, including.evidence = TRUE), grid = TRUE, scale = c(0.7, 1.0), main = paste("Cover", state))

}

# Arc strength evaluations

arc.strength(NFhc, data = NFdata, criterion = "x2")

arc.strength(NFhc, data = NFdata, criterion = "bic")

NF.strength <- bf.strength(NFhc, NFdata, score = "bde", prior = "marginal")

# Predictive performance

NF.fitted <- bn.fit(NFhc, NFdata)

predictions <- predict(NF.fitted, node = "nest_fate", data = NFdata)

actual <- NFdata$nest_fate

# Performance assessment

predictions_numeric <- as.numeric(as.factor(predictions))

actual_numeric <- as.numeric(as.factor(actual))

roc <- prediction(predictions_numeric, actual_numeric)

perf <- performance(roc, "tpr", "fpr")

plot(perf, main = "ROC Curve")

auc <- performance(roc, measure = "auc")

print(paste("AUC Value:", auc@y.values[[1]]))

# Cross-validation

bn.cv(NFdata, 'hc', loss = "logl", method = "hold-out", k = 10, m = 10)

bn.cv(NFdata, 'hc', loss = "pred", loss.args = list(target = "nest_fate"))

# Query examples

querygrain(NF.junction, nodes = c("Soil_cluster", "NWD", "NVD", "nest_fate", "Location", "Cover_cluster", type = "marginal"))

**Fig E.** Code of nest failure PCA analysis

# Load data

Faildata <- read.csv("File_5.csv", header = TRUE)

# Select target variables for PCA

target_variables <- c("Total_vegetation_cover", "Sand_cover", "Granule_Pebble_cover",

"Cobble_cover", "Object_cover", "Shell_cover",

"Very_coarse_sand", "Coarse_sand", "Medium_sand",

"Fine_sand", "Very_fine_sand")

# Subset and clean data

pca_data <- na.omit(Faildata[target_variables])

# Perform PCA with scaling

pca_result <- prcomp(pca_data, scale. = TRUE)

# Summarize PCA

summary(pca_result)

pca_result$rotation

# Scree Plot

explained_var <- pca_result$sdev^2 / sum(pca_result$sdev^2)

barplot(explained_var,

main = "Scree Plot",

xlab = "Principal Component",

ylab = "Proportion of Variance Explained",

col = "steelblue")

# Visualize PC1 vs PC2

library(ggplot2)

library(ggrepel)

pca_scores <- as.data.frame(pca_result$x)

pca_scores$Fail_reason <- Faildata$Fail_reason

ggplot(pca_scores, aes(x = PC1, y = PC2, color = Fail_reason)) +

geom_point(size = 3, alpha = 0.8) +

stat_ellipse(aes(fill = Fail_reason), geom = "polygon", alpha = 0.2, color = NA) +

labs(title = "PCA Plot by Fail_reason", x = "PC1", y = "PC2") +

theme_minimal()

# Component Loadings

loading_matrix <- pca_result$rotation

loading_df <- as.data.frame(loading_matrix)

loading_df <- cbind(Variable = rownames(loading_df), loading_df)

# Top contributing variables to PC1

library(dplyr)

top_variables_pc1 <- loading_df %>% arrange(desc(abs(PC1))) %>% head(5)

top_variables_pc1

# Loadings for PC1 & PC2

arrow_data <- as.data.frame(loading_matrix[, 1:2])

arrow_data$Variable <- rownames(arrow_data)

# Final PC1 vs PC2 Plot with Loadings

arrow_scale <- 5

final_plot <- ggplot(pca_scores, aes(x = PC1, y = PC2, shape = Fail_reason)) +

geom_point(size = 2, color = "black") +

scale_shape_manual(values = c("Flooding" = 1, "Predation" = 19, "Unknown" = 4)) +

stat_ellipse(data = subset(pca_scores, Fail_reason %in% c("Flooding", "Unknown")),

aes(fill = Fail_reason), geom = "polygon", alpha = 0.2, color = "black") +

scale_fill_manual(values = c("Flooding" = "white", "Unknown" = "grey")) +

theme_minimal() +

annotate("segment", x = -7, xend = 7, y = 0, yend = 0,

arrow = arrow(length = unit(0.3, "cm"), ends = "both"), size = 0.8) +

annotate("segment", x = 0, xend = 0, y = -7, yend = 7,

arrow = arrow(length = unit(0.3, "cm"), ends = "both"), size = 0.8) +

geom_segment(data = arrow_data,

aes(x = 0, y = 0, xend = PC1 * arrow_scale, yend = PC2 * arrow_scale),

arrow = arrow(length = unit(0.3, "cm")), size = 1, color = "black") +

geom_text_repel(data = arrow_data,

aes(x = PC1 * arrow_scale, y = PC2 * arrow_scale, label = Variable),

size = 5, box.padding = 1, point.padding = 1, segment.color = 'grey50') +

scale_x_continuous(limits = c(-7, 7)) +

scale_y_continuous(limits = c(-7, 7)) +

coord_fixed(ratio = 1)

print(final_plot)

```

> 📌 The script also includes additional PC2 vs PC3 visualizations and interpretation code if needed for extended analysis.
