# Supplementary material for: Estimating nesting habitat characteristics for the Kentish plover (Anarhynchus alexandrinus) with the effect of substrate and vegetation using a Bayesian network approach
Source: PLoS One. 2025 Jun 11;20(6):e0325750. doi: 10.1371/journal.pone.0325750 (PMC12157252; doi:10.1371/journal.pone.0325750)
Supplement: Attachment — Submitted filename: Taslima Reviewer Plos One.pdf [file pone.0325750.s002.pdf]

Upon reviewing the manuscript, I found several areas where improvements are necessary. Below is a summary of my observations and suggested revisions:

Major Revision Suggestions

1. **Clarity and Focus**
    - The manuscript occasionally veers into overly detailed descriptions that can distract from the main points. Streamline the text to ensure the central findings remain the focus. For example:
        - **Introduction:** Clearly define the research gap in the opening paragraph instead of detailed background information.
        - **Discussion:** Focus more on the implications of findings rather than repeating results.
2. **Figures and Data**
    - Ensure all figures (e.g., Fig 1, 3B) are cited in the text and are adequately explained. Some figures lack detailed captions, making it difficult to understand them independently.
    - Consider reformatting the results section to integrate tables or figures more effectively to support key findings.
3. **Methodology**
    - Add clarity regarding statistical methods. While Bayesian Network (BN) modeling is well-presented, details about the assumptions or limitations of the approach should be expanded in the discussion.
    - Specify why the Mangyeong and Dongjin sites were chosen over other possible reclaimed lands. This strengthens the argument for the study's relevance.
4. **Grammar and Language**
    - Several grammatical errors and awkward phrases throughout the text need revision for fluency. Examples include:
        - "Because, the parent Kentish plovers..." should be "This is because parent Kentish plovers..."
        - Replace "ceaseless habitation loss" with "continuous habitat loss."
    - Consistent terminology: Ensure consistent use of terms like "Kentish plover" versus "Kentish Plovers."
5. **Ethics Statement**
    - Expand the Ethics section slightly to include how non-invasive methods minimized disturbances to Kentish plovers.
6. **Conclusion**
    - The conclusion should emphasize how the study's findings are applicable to global conservation strategies for shorebirds. Currently, it focuses too narrowly on Saemangeum.
