# Supplementary material for: Estimating nesting habitat characteristics for the Kentish plover (Anarhynchus alexandrinus) with the effect of substrate and vegetation using a Bayesian network approach
Source: PLoS One. 2025 Jun 11;20(6):e0325750. doi: 10.1371/journal.pone.0325750 (PMC12157252; doi:10.1371/journal.pone.0325750)
Supplement: Attachment — Submitted filename: Dear Editor.pdf [file pone.0325750.s004.pdf]

Dear Editor,

I have reviewed the manuscript titled " Estimating nest habitat of Kentish Plover in reclaimed lands using Bayesian network and geological criteria" and found it presents an interesting analysis of the reproductive success of Kentish Plover *(Anarhynchus alexandrinus*) in South Korea using a Bayesian method. While generalizing environmental influences on nesting across species is challenging, this study offers a valuable approximation of the specific conditions and resource utilization within this region.

I have some suggestions for improvement:

Title:

The current title is lengthy. Please revise it to a maximum of 20 words.
Remove "reclaimed" and "and geological criteria" from both the main title and any subtitles, because it falls outside the scope of this study

Keywords:

Ensure keywords are unique and do not duplicate terms already present in the title or abstract.

Methods:

- Include a figure or map illustrating the study area's composition (e.g., natural areas in green, anthropogenic areas in grey, aquatic areas in blue).
- Provide a detailed description of the materials used.
- Add photographs of the study area, materials, and some nests.
- Review the bibliographic citation for the R software usage.

Results:

I was unable to access the supplementary material.
To address this, consider incorporating a table or figure directly into the manuscript to illustrate key statistical data, enabling readers to understand the results without relying on supplementary files.

Discussion:

The study does not directly compare reclaimed versus non-reclaimed lands. Therefore, remove "reclaimed" from the title.

Consider merging the final two sections (Recommendation and importance on the construction of artificial coastal breeding habitats for Kentish plovers and Future studies regarding nesting sites of Kentish plovers) into a maximum of three paragraphs. This would streamline the presentation, minimize repetition of information from the introduction, and allow for focused recommendations.

Conclusion:

Rewrite the conclusion, focusing solely on the findings specific to the study area in Korea. Conclude with a statement emphasizing the importance of conserving the nesting areas.
