# Supplementary material for: Estimating nesting habitat characteristics for the Kentish plover (Anarhynchus alexandrinus) with the effect of substrate and vegetation using a Bayesian network approach
Source: PLoS One. 2025 Jun 11;20(6):e0325750. doi: 10.1371/journal.pone.0325750 (PMC12157252; doi:10.1371/journal.pone.0325750)
Supplement: Attachment — Submitted filename: Kentish_plover_nest_modelingreview BH.docx [file pone.0325750.s003.docx]

**Full title: Estimating the characteristics of nesting habitat for the Kentish Plover (*Anarhynchus alexandrinus*) in artificially reclaimed lands with the effect of substrate and vegetation based on a Bayesian network approach and geological criteria**

**Short title: Estimating nest habitat traits of Kentish Plover in reclaimed lands using Bayesian network and geological criteria**

Dong-Yun Lee^1^, Ju-Hyun Lee^1^, Jong-Ju Son^2^, Seung-Jun Oh^1^, Ha-Cheol Sung^1*^

^1^ Department of Biology, Chonnam National University, Gwangju, 61186 Republic of Korea

^2^ School of Biological Science and Biotechnology, Chonnam National University, Gwangju, 61186 Republic of Korea

**Corresponding author:** Ha-Cheol Sung; shcol2002@jnu.ac.kr; +82-62-530-3417

**Abstract**

Coastal habitats play an important role in the nesting of shorebirds but are increasingly threatened by human activity and ceaseless habitation loss. To conserve the shorebird population, it is necessary to understand how artificial coastal areas can be used for shorebirds. Substrate particle size and vegetation cover are key environmental factors that affect the nest site selection and nest success of ground-nesting shorebirds like plovers. We analyzed substrate particle size and vegetation cover in the Kentish Plover (*Anarhynchus alexandrinus*) nesting sites within an artificial coastal area, the Saemangeum reclaimed land in 2020 using geological criteria and 1-m^2^ quadrat photos developing Bayesian network (BN) models to analyze their impact on nest site selection and success. The BN models predicted how substrate particle size and vegetation cover influence the possibility of nest/non-nest and successful/unsuccessful nest sites of Kentish plovers. Our results indicated that Kentish plovers prefer sandy sites with moderate vegetation and achieve higher nest success in habitats with mixed soil types, including medium (0.25–0.5 mm), fine (0.125–0.25 mm), and very fine (0.063–0.125 mm) sand with small proportions of mud (<0.063mm). Kentish plovers exhibited a preference for nesting and showed higher nest success in areas with moderate vegetation compared to those with no vegetation or overly dense vegetation. Thus, the complex interactions between plover nests and substrate characteristics, including soil porosity and permeability, must be carefully evaluated. Vegetation cover also should be carefully managed with consideration for the trade-offs in predation risk, nest camouflage, nest crypsis, and thermoregulation associated with plover nesting preference and success. Our study provides essential quantitative insights and emphasizes the need to consider multifaceted ecological factors with inherent uncertainties in coastal environments to optimize artificial coastal habitats for the conservation of shorebirds based on an investigation of the nesting habitat traits of Kentish plovers.

*Keywords*: Bayesian network model, Kentish Plover, Nesting habitat, Reclaimed land, Shorebird conservation, Substrate particle size, Vegetation cover

**Introduction**

Coastal areas are vital breeding habitats for shorebirds, but ongoing coastal developments are ceaselessly diminishing shorebird populations [1]. To protect shorebirds, considering how to return artificial coastal areas, which have already been developed, to them is necessary for restoration and conservation projects. Indeed, such habitats are capable of serving as breeding sites for shorebirds [2]. In the Asia-Pacific region, many shorebird species have been observed frequently utilizing artificial coastal areas as habitats [3]. However, prior to conducting the creation of artificial coastal habitats, especially on the artificial breeding sites, investigating the association between environmental factors and nesting sites of ground-nesting shorebirds is important because it can provide appropriate information regarding restoration and conservation projects in coastal habitats, such as dunes and beaches [4]. Because, environmental factors, such as wind, humidity, soil, and vegetation, in coastal areas influence the nesting of shorebirds in diverse ways [5]. Ground environmental factors like substrate and vegetation, in particular, play a significant role in nest site selection and nest success of ground-nesting shorebirds [6].

Most plovers of family Charadriidae are ground-nesting shorebirds, and their selection of nesting sites is associated with environmental factors [6] as they prefer coasts with suitable habitat characteristics, such as specific particle size of the ambient substrate and appropriate range of vegetation cover for nesting. The preference of each plover species determines the patterns of nest site selection and influences nest success and failure [7]. First, plovers are known to select a site for nesting depending on specific substrate cover particle sizes [8]. Body and egg-size stones are useful for nest camouflage and crypsis [9]. On the other hand, small substrates differently affect the nesting of plovers. For example, the distribution of soil particles (sand and mud) correlates with soil permeability and porosity [10]. Decreased soil permeability and porosity slow underground water absorption [11] and can cause more flooding in an area [12]. Considering that flooding is a major reason for nest failure, the distribution of soil particles in habitats can influence the nesting sites of plovers [13–14]. Second, vegetation cover in habitats is associated with nest site selection and nest success of plovers. Most plovers prefer areas with reduced vegetation cover for nesting [15] because high vegetation cover hinders incubating the ability of plovers to detect approaching predators [16]. Nevertheless, the presence of vegetation in nesting sites occasionally improves the nest success of plovers. Because, incubating plovers may use vegetation to conceal their offspring from detection by predators, although they may also be vulnerable to attacks because the dense vegetation can create visual obstacles preventing the detection of approaching predators [17].

In this research, we studied on nesting habitats of the Kentish Plover (*Anarhynchus alexandrinus*). The nesting sites include coasts, such as beaches and estuaries [18–19]. Kentish plovers have been known to be influenced by environmental factors such as the landscape and climate of habitats [20]. The population of Kentish Plovers is predicted to diminish at a greater rate than its current level as a consequence of both anthropogenic and climate change effects, and the safeguarding of this species is vital for the conservation of other species, given the role of this species' habitats as umbrella habitats [21]. Indeed, the breeding sites of Kentish plovers have been reported to overlap with those of other shorebirds [22–23]. Furthermore, one of the ongoing endeavors is to utilize the Kentish plover, as an indicator species in tidal flats within the context of the coastal environment in South Korea [24].

In particular, in the coastal habitats, substrate and vegetation are common environmental factors that show complicated relationships with the nesting of Kentish plovers. First, coasts of various substrate types influence nest site selection and nest success of Kentish plovers. Most of them prefer sandy coasts to areas covered by rocks and stones for nesting [25]. However, Kentish plovers in some regions select nesting sites in rocky coasts [26]. On the fine-scale substrate of nesting sites, mud substrate was found to decrease the nest survival rate of Kentish plovers in Bohai Bay, China [27]. Second, the vegetation in habitats is complexly related to the nesting of Kentish plovers. These plovers have been known to avoid nesting in areas with vegetation cover [28], because it interferes with the ability of parent birds in detecting predators approaching their nests [29]. This can have a negative impact on their breeding [6]. However, high vegetation cover in a nest site can increase the probability of nest survival because an incubating bird can hide from predators in the vegetation, thus contributing to nest success [16]. Thus, vegetation cover rate and presence/absence have a more significant influence on their nesting than vegetation species composition [30].

Determination of the most suitable substrate type and vegetation cover rate for the nesting sites of Kentish plovers is not simple and demands a comprehensive analysis. Hence, we developed models to predict nest presence/absence and success/failure depending on environmental factors, such as substrate type (particle size) and vegetation cover. First, we examined the substrate characteristics of nest sites to determine the particle size that has the greatest influence on nest site selection and nest success of Kentish plovers. We described differences in the range of particle sizes observed between nest/non-nest sites and successful/unsuccessful nest sites. We then needed to examine the effect of particle size based on a finer scale on the nest success of Kentish plovers. We thus demonstrated differences in the rate of each particle size between successful and unsuccessful nest sites. Furthermore, we tried to follow geological criteria in the substrate analysis of plover habitats. The various studies of plover habitats have established particle size criteria in disparate ways, with the specific criteria varying depending on the research aims [7, 9]. However, in order to provide more precise information regarding particle size for the restoration and conservation of plover habitats, researchers must collaborate with geologists, including soil scientists. In the field of geology, the classification and categorization of grain particle size have been established for over a century [31–32]. These scales are utilized extensively within the geological community, including by the United States Geological Survey (USGS). Accordingly, we felt the necessity of geological criteria in this study and made use it in our analysis of the substrate, specifically the soil and stone components of the plover habitat. Second, we investigated the impact of vegetation cover on nest site selection and nest success of Kentish plovers. We have avoided providing a direct answer to how vegetation affects nest site selection and nest success of Kentish plovers because of the complicated associations. Rather, we described the differences in vegetation cover at nest and non-nest sites as well as successful and unsuccessful nest sites. In addition, we analysed environmental factors other than substrate and vegetation in the study areas to determine whether they have a greater effect on nest site selection and nest success of Kentish plovers.

In this research, we undertook a meticulous process to choose an appropriate field survey area. Firstly, in order to provide the breeding information in artificial habitats of Kentish plovers, it was necessary to conduct the field survey in artificial locations. In particular, we selected the artificial locations actively used as habitats by shorebirds including Kentish plovers for our study. Secondly, in order to gain insight into the diverse relationships between Kentish plovers and their surrounding substrates and vegetation, it requires obtaining a comprehensive range of data pertaining to the nesting patterns of this species within a specific area. Had we selected multiple regions, we would have been unable to ascertain whether the observed preferences of Kentish plovers were attributable to regional differences or environmental factors. Therefore, we selected a specific coastal and artificial location with diverse substrate and vegetative conditions as the subject of our investigation.

We employed a Bayesian network (BN) modelling approach. BN models offer several advantages, including direct graphical representation, structure learning, and integration of diverse data sources [33]. This method has been successfully applied in previous studies on the piping plover (*Charadrius melodus*) to identify key habitat factors and support conservation planning [34–36]. Likewise, we implemented BN models to assess the impact of substrate and vegetation on the nest site selection and nest success of Kentish plovers.

Hence, the following were our research purposes: (1) to examine the differences in environmental factors, including substrate type and vegetation cover between the nest or non-nest sites and successful or unsuccessful nest sites of Kentish plovers, (2) to estimate the characteristics of nest sites and successful nests of Kentish plovers depending on the rate of environmental cover and soil particles and (3) to provide information on the artificial coastal habitats of Kentish plovers using modelling works for restoration and conservation.

**Materials and Methods**

**Study sites**

This study was performed from March to July 2020 at the following estuaries in Saemangeum (Fig 1): (1) Mangyeong (1,011 ha), Gunsan-si, and Jeollabuk-do (35°52'N, 126°40'E) and (2) Dongjin (893 ha), Buan-gun, and Jeollabuk-do (35°48'N, 126°38'E). Over ten thousand shorebirds have utilized these areas as stopover sites along the East Asian–Australasian Flyway in every season, and Kentish plovers are regularly observed during both the migration and breeding seasons [37–38]. Developments in Saemangeum have demonstrated a negative correlation with avian populations. Because, the ongoing and progressive reclamation of these lands is exerting a detrimental impact on the habitats of shorebirds [1].

Our research areas in Saemangeum were designed for agricultural use and are currently inaccessible to the general public due to ongoing national development [39]. Accordingly, authorization from the Saemangeum Project Office in the Korea Rural Community Corporation was required for our research activities (Official Document Number: College of Natural Sciences-984, Jan 23^th^ 2020). We could observe a variety of shorebirds in there with minimal human interference due to the temporary cessation of development in our study sites.

Research sites were composed of land parcels separated by roads (paved in asphalt, concrete, gravel, and sand). The parcels exhibited not only disparate environmental characteristics but also a range of traits within each parcel like mixed dense vegetation areas on one side and sandy areas on the other side in a single parcel. Halophile plants were widely distributed in these areas. The dominant species of halophytes in tidal flats were *Suaeda japonica* and *Salicornia europaea*, with those in reclaimed land halophytes dominated by *Spergularia marina*, *Phacelurus latifolius*, *Phragmites australis*, and *Calamagrostis epigejos*. These areas will likely transform salt marsh and exposed sandy areas into grassland [40].

**Surveying methods**

To ascertain the nest fate of Kentish plover, we utilized two methods: floatation and nest success/failure evidence. On the target nests, we estimated the hatching date based on the flotation method using warm water (37–39 °C) and a bottle [41]. Next, we monitored the nests at least two times per week to check for nest fate. The evidence of nest success and failure in this study was decided based on indicators reported in previous studies [16, 29, 42].

**Data collecting methods**

To document substrate and vegetation cover within an area of 1 m², we took 1-m² quadrat photos at a height of 1.5 m in the following two ways for each nest: (1) centered on the nest within 1 m² (representing a nest site) and (2) two sites randomly selected at a distance of 10 m from a nest (representing two non-nest sites). If a randomly selected site was near other nests of the Kentish plovers (<1 m), we excluded it and chose another one. We estimated the cover rate of eight environment variables in the quadrat photos (Table 1). The particle size of quadrat photos was determined depending on geological criteria [31–32]. Detailed vegetation species composition data were not included in this study, because vegetation cover rate plays a much more essential role [30]. Only one person measured the cover rate of variables to eliminate errors that arise when multiple people take measurements. Photo analysis was performed using the Scalar imaging system, version 2.88.

To investigate nest characteristics other than those in the quadrat photos, we measured the distance from a nest to two environmental factors (vegetation and water source) and collected a soil sample from a nest (Table 2). First, we directly measured the distance from a nest to the nearest vegetation (m) in the field. We also recorded the GPS location of the target nests using a satellite map (Kakao map) to calculate the distance from each nest to the nearest water source (m). Second, we analyzed the soil particle size of each nest by granulometric analysis using a fine-scale than that used in quadrat photo analysis (Fig A in S1 File). We collected a soil sample 30 cm away from a nest based on the previous study investigating the relationship between soil particles and nest site selection in the Campo Miner (*Geositta poeciloptera*) [43]. We dug to a depth of 2–3 cm at a point considering the nest depth of 2.76 ± 0.57 cm [44]. Before analyzing particle size, we laid the soil samples in an acrylic cage for at least four hours to dry them in the sunlight. Sieving wet soil was difficult owing to soil cohesion. Next, we sieved the dry samples to classify the soil particles based on size. We compared the total weight of sieve residues with the weight of the original sample. If there was more than a 2% discrepancy, we had to measure a sample again to decrease the error rate [45]. The distribution of particle size in our study was based on previously reported geological criteria [31–32].

**Modelling process and statistical analysis**

We used R software, version 4.3.1 (R Foundation for Statistical Computing, Vienna, Austria) to perform cluster analysis and build models using a BN model. Before the BN modelling process, we discretized all variables to set “nodes”: (1) median and quartiles, (2) presence/absence, and (3) clustering. We preferred the median and quartiles method for each variable over other discretizing methods. If we could not use this method for a variable that showed too many zero rates, we discretized it by considering presence/absence. We also utilized cluster analysis for variables that did not show large variations and did not link with other variables through “arcs”. We performed K-means cluster analysis using the *factoextra* package v1.0.7 [46]. When we chose the number of clusters, we referred to the graphs made using the elbow and silhouette methods. We then compared the average of the variables among the clusters. We named each clustering group in a variable using an alphabet or number.

To construct BN models, we made a primitive model using a Hill-climbing (HC) algorithm based on the Bayesian information criterion (BIC) score using the *bnlearn* package v4.6.1 [47]. We then set a location node as the first node and nest designation or nest fate nodes as the last. Considering the original causal relationships of variables, we also adjusted it based on HC models (ex. dry and live vegetation rates always influenced total vegetation rates, not the opposite). We described the strength values of arcs and the BIC score of nodes. We examined the sensitivity and accuracy of each model algorithm. To calculate model sensitivity, we used “the receiver operating characteristic (ROC) curve” to estimate “the area under ROC curve (AUC) value” (at least over 0.5 AUC value) [48]. To estimate model accuracy, we calculated the expected loss of models using 10-fold cross-validation and used classification error for the loss function (0 = no error, 1 = complete error) [49].

We constructed and trained the following two models: (1) the nest site selection model (comparison between the nest and non-nest sites) and (2) the nest fate model (comparison between successful and unsuccessful nests). First, we constructed the nest site selection model of Kentish plovers using variables of the 1-m² quadrat photos (Table 1). We connected the following: (1) a node regarding nest site designation, (2) a node regarding research location, (3) three nodes representing the presence/absence of granule + pebble, cobble, and object, and (4) five nodes representing discretized continuous cover rate variables, namely sand, shell, dry vegetation, live vegetation, and total vegetation cover (dry vegetation + live vegetation) using median and quartiles. Then, we predicted the 100% possibility models of (1) nest/non-nest site and (2) the most related substrate/vegetation variable cover. Second, we constructed the nest fate model of Kentish plovers using five variables (Table 2), comprising the following: (1) a node regarding nest fate (nest success and fail), (2) a node regarding research location, (3) two nodes representing discretized continuous variables, described as the distances from the nest to the nearest vegetation and distance from the nest to water based on the median and quartiles of each variable, and (4) two nodes, indicating cover type and soil type, representing the group of nest environment characteristics. To group cover types, we used seven cover variables in the quadrat photos (dry vegetation, live vegetation, sand, granule + pebble, cobble, object, and shell). To group soil types, we used seven particle size variables (gravel, very coarse sand, coarse sand, medium sand, fine sand, very fine sand, and mud). We then predicted the 100% possibility models of (1) nest success/failure and (2) cover/soil type.

We conducted Principal Component Analysis (PCA) with the *stats* package [50] to find the relationships between nest failure reasons and environmental variables. Before PCA, we performed Spearman’s correlation tests to prevent multicollinearity among highly positive related variables using the *psych* package [51].

**Ethics statement**

The first author has completed a formal training program on animal welfare and ethical research practices (Certificate No. CNU IACUC-2020-107), issued by Chonnam National University (CNU) and CNU Laboratory Animal Research Centre, to ensure adherence to ethical standards during the research process. No approval from any research ethics committee was required for this study as we did not capture, handle, or manipulate any birds. Despite no required approval, all data were collected through non-invasive methods in strict compliance with the current laws and regulations of South Korea to minimize the disturbance of wildlife animals.

**Results**

Of the 101 nests of Kentish plovers that we found, we used the dataset of 95 nests for analysis, as six nests in Mangyeong were decided as outliers (three nests on the rice field ridge paved with gravels; three nests discovered at the hatching date). 60 were in the 21 land parcels of Mangyeong (33 successful and 27 unsuccessful nests), and 35 in the 12 land parcels and an exposed dry riverbed of Dongjin (28 successful and 7 unsuccessful nests). Out of 34 failed nests, failure reasons were flooding in 21 nests, unknown in 11 nests, and depredation in two nests (Fig B in S1 File).

We took 285 quadrat photos (95 on nest sites and 190 on non-nest sites), based on which, we prepared the nest site selection model of Kentish plovers. To construct the nest fate model, we used a dataset including quadrat photos, soil particle size, and distance data. In addition, we observed that many other shorebirds including the Little Tern (*Sternula albifrons*) and the Eurasian Oystercatcher (*Haematopus ostralegus*) bred in the study areas. We also were able to watch a variety of terrestrial predators like the dog (*Canis familiaris*) and the common raccoon dog (*Nyctereutes procyonoides*) as well as aerial predators such as raptors (*Falco tinnunculus*, *F. peregrinus*, *F. subbuteo*, *Circus cyaneus*, and others) and owls (*Bubo bubo*) with egg predators as herons (*Ardea cinerea*, *A. coromandus*, *A. abla*, and others).

**Nest site selection model**

The nest site selection model of Kentish plovers demonstrated the relationships among eight environmental factors with a locality variable and a nest site designation factor (Fig 2). The ROC curve showed 0.65 AUC value. The expected loss rate was 0.33. The strength values were the highest in the arc from total vegetation cover to nest site designation and the lowest from total vegetation cover to sand cover (Table A in S1 File). On the nodes, the lowest BIC score was the nest site designation node and the highest, was the cobble node (Table B in S1 File).

This BN model revealed the distinctions between the two models of nest site and non-nest site. First, in the nest site BN model of Kentish plovers (Fig A in S2 File), Kentish plovers prefer sandy areas (< 73% sand cover, 30% possibility; 73–89% sand cover, 33% possibility) with some vegetation cover (0–20% total vegetation cover, 66% possibility) rather than only sandy areas (> 97% sand cover: 10% possibility). Second, in the non-nest site BN model of Kentish plovers (Fig B in S2 File), the possibility of the <20 % category was the highest in the total vegetation cover node. The possibilities of the 89–97% and >97% categories were higher than those of the <73% and 73–89% categories in the sand cover node. Other variables did not show large differences between those two models.

Next, the BN model showed the differences among cover rates. First, in the sand cover BN models of Kentish plovers, the possibility of a nest site in a nest designation node was over 40% in low sand cover models and less than in high sand cover models (Fig C in S2 File). Second, in the total vegetation cover BN models of Kentish plovers, the possibility of a nest site in a nest designation node was the highest in the 20–90% total vegetation cover model and the lowest in the <20% total vegetation cover model (Fig D in S2 File).

Last, the model demonstrated the local variations in environmental cover rate (Fig F in S2 File). The vegetation cover rate in Gunsan was higher than in Buan, while the sand cover rate was lower.

**Nest fate (nest success and fail) model**

For cluster analysis, we discretized two variables cover and soil types. We set the optimal numbers of clusters based on the results of the silhouette and elbow methods (Fig C–D in S1 File). First, the cover rates of nests in the 1-m^2^ quadrat were grouped into two categories (types A and B, Fig 3A). In the type A group, sand cover was at the highest rate, followed by dry vegetation cover (sand, 85.52%; dry vegetation, 5.68%). In the type B group, dry vegetation cover was the main variable and the sand cover was the next major variable (dry vegetation, 48.64%; sand, 34.67%). Second, the soil particle size of the nests was grouped into three categories (types 1, 2, and 3, Fig 3B). In the type 1 group, fine sand was the main variable and very fine sand, was the second major variable (fine sand, 53.44%; very fine sand, 27.11%; medium sand, 10.59%). The type 2 group exhibited similarities to the type 1 group, although the differences between the rates of particle types in the type 2 group were lesser than those in the type 1 group (fine sand, 26.57%; very fine sand, 23.81%; medium sand, 20.93%). In the type 3 group, the main variable was very fine sand, and the second major variable was fine sand (very fine sand, 40.79%; fine sand, 35.58%). The mud rate was the lowest in type 1 (4.62%) and over 10% in type 2 (13.57%) and 3 (11.52%).

The nest fate BN model of Kentish plovers was composed of four variables with a locality variable and a nest fate factor (Fig 4). This model showed 0.70 AUC value. The expected loss rate was 0.33. The strength values were the highest in the arc from the distance between a nest to water source to nest fate and the lowest from locations to soil type (Table C in S1 File). On the nodes, the lowest BIC score was the distance between a nest and water source node and the highest, was the cover type node (Table D in S1 File).

The BN model demonstrated the distinctions between the two models of nest success and failure (Fig G in S2 File). First, in the nest success BN model of Kentish plovers, the successful nests of Kentish plovers are related to the areas including sand dominant (type A) with fine and very fine sand (type 3). Second, in the nest fail BN model of Kentish plovers, type A and type 1 showed a higher possibility than did type B, type 2, and type 3.

Cover and soil types differed in each BN model. Firstly, in the cover type BN models of Kentish plovers, the possibility of nest success in a nest fate node was lower in the cover type A model than in cover type B (Fig H in S2 File). Secondly, in the soil type BN models of Kentish plovers, the possibility of nest success in a nest fate node was lower in the soil type 1 model than in soil type 2 and 3 models (Fig I in S2 File). Finally, NVD and Soil type nodes exhibited larger differences depending on locations. In particular, soil type 2 was 0% in Gunsan (Fig J in S2 File).

**Relationships between nest fail reasons and environmental variables.**

We used 11 variables because dry and live vegetation highly positively correlated with total vegetation, gravel did with very coarse sand, and mud did with very fine sand (*r* > 0.70; *p* < 0.05). Given from PC1 to PC3 (% of total variance = 63.83%; Table E in S1 File), flooding correlated with most soil particle sizes, but unknown did with most of the cover variables (Fig 5).

**Discussion**

We conducted a comprehensive study to provide quantitative information and estimate the characteristics of nesting sites of Kentish plovers in artificial coastal areas, Saemangeum reclaimed lands, to contribute to the conservation and restoration of shorebird breeding habitats. In particular, focusing on substrate type and vegetation cover in coastal habitats, we also performed a multifaceted study to clarify the role and association of ground environmental factors with nesting sites of the Kentish Plover. We identified that substrate and vegetation are essential for nest site selection and nest success of this plover species. Kentish plovers were found to prefer sandy coasts with moderate vegetation cover for nesting, and their nest success was directly related to specific particle sizes.

**Effect of substrate types and particle size on nest site selection and nest success of Kentish plovers**

We found that substrate types have the most closed and direct relationships with the nest site selection and nest success of Kentish plovers. Our BN models showed sand cover has a greater influence on the selection of nesting sites by Kentish plovers than do other substrates, such as shell, gravel, pebbles, and cobbles. This result conforms with that of other studies [25, 28, 52]. In particular, our models revealed that soil particle sizes correlate with the nest success of Kentish plovers. The possibility of nest success was higher when habitats were composed of mixed soil from medium sand to mud, in addition to only fine and very fine sand. We also revealed that the flooding in nest failure had a relationship with soil particle sizes through PCA. Therefore, we speculated that soil particle sizes may affect the nest success of Kentish plovers and suggested that further research is required to clarify the mechanism by which soil particle sizes precisely influence the breeding of Kentish plovers. One possible reason is that soil permeability and porosity may affect the nest success of Kentish plovers, because we observed that flooding due to heavy rain was the predominant cause of nest failure. We did not consider tidal effects in this study because our study areas in Saemangeum are always higher than sea level, irrespective of the type of tide (high or low and neap or spring). Only rainfall and drainage influenced flooding events in these areas. Additionally, the presence of mud as tiny substrates could negatively affect the nest survival rate of Kentish plovers because of poor soil permeability with increasing flooding occurrences [27]. In contrast, our BN models indicated that an insufficient mud composition in habitats is negative to the success of Kentish plover nests. Thus, future studies focusing on determining the relationship of soil permeability and porosity with the coastal nesting sites of plovers are required based on data regarding soil sample volumes, which we did not acquire, with pore space rates.

Despite our specific analysis of substrate as soil properties, we could not exactly suggest that only soil including mud and sand is an important factor for the nesting sites of Kentish plovers. Because, the parent Kentish plovers of our target nests used various materials, including shells, dry vegetation, and dry soil particles to construct a nest (shells were used in 18 nests; dry vegetation, in 33 nests; dry soil particles, in 5 nests; >two major types of material mixed, in 22 nests; and sand main, in 17 nests; Fig F in S1 File). Furthermore, three pairs of Kentish plovers selected positions on the rice field ridge paved with gravel for nesting (Fig F in S1 File), although this data was excluded from our analysis. In other locations, Kentish plovers largely selected nesting sites in the shingle beaches of northern France [53], the rocky coasts of north-west Morocco [26], and the saltpans covered by abundant shells of southern Spain [23]. Thus, it needs to be investigated whether other substrates, in addition to sand, influence the nesting of Kentish plovers.

**Effect of vegetation cover on nest site selection and nest success of Kentish plovers**

Given our models, Kentish plovers are more likely to select sandy habitats with moderate vegetation cover rates for nesting sites than only sandy habitats, which was an unexpected result. We originally hypothesized that Kentish plovers prefer sandy areas with no vegetation to sandy areas covered by some vegetation for nesting. When only nest sites and success models were considered, Kentish plovers were found to prefer low vegetation cover habitats for nesting, and their nest success increased in these types of habitats. This may be attributed to the fact that reduced vegetation in the habitat helps incubating Kentish plovers better detect approaching predators [15]. However, in this study, when the possibility of vegetation was designated as "present," the possibilities of nest site and success were observed to be higher than when it was designated as "absent." Additionally, the possibility of a nest site was equal when that of vegetation was set as "absent" and "dense."

In consideration of our modelling results, we proposed that “survivorship bias [54–55]” may be associated with the negative impact of vegetation on the nesting of plovers. To be specific, finding exposed nests is much easier for terrestrial predators than finding other nests situated close to vegetation.For example, we discovered three nests in the hatching date in this study. The dense vegetation in the nesting areas made it impossible to find them earlier, even though we frequently visited and observed the same places every two days. Additionally, in the densely vegetated locations, we often discovered the nests of Kentish plovers directly beneath our feet, just before our footfalls. Therefore, these cases provided insight into the role of vegetation in safeguarding against predator detection, including humans.

Regarding the positive relationship between vegetation and the nests of plovers, several hypotheses have been suggested. First, vegetation cover protects nests and incubates plovers from predators [56]. Incubating adults cannot detect predators easily in areas covered by vegetation because of visual interference; conversely, vegetation is useful for incubating animals because it helps hide them from predators. This means that there exists a trade-off between nest crypsis and predation risks [16]. Indeed, we found two depredated nests and 11 nests, which was unknown on the reason for nest failure. If predators consumed the eggs of these 11 nests, their predation traces such as partial egg fragments would not remain because some predators eat eggs swallowing at ones [52, 57]. Furthermore, in our PCA results, the tendency of unknown failure reason was related to vegetation cover, which highly affects predation events and nest crypsis. This indicated that predation is the second major reason for nest failure, next to flooding in this study. Second, nests covered by vegetation can provide more stable thermal conditions to eggs than open nests [58]. In Kentish plovers, parent plovers demonstrate an ability to regulate nest temperature sensitively, exhibiting a trade-off between nest camouflage and the establishment of an appropriate nest temperature [59]. Therefore, vegetation cover in the breeding sites of Kentish plovers should be considered a complex factor that contributes to predation risks, nest crypsis, and thermoregulation.

**Recommendation and importance on the construction of artificial coastal breeding habitats for Kentish plovers**

We observed that the land parcels in reclaimed lands could play a role in breeding habitats for Kentish plovers. We therefore examined the optimal characteristics of their nesting site based on our model predictions. Prior to the initiation of conservation and restoration projects, we would like to raise the need to consider that Kentish plovers may select moderate vegetation-covered areas with less than 90% sand cover and can successfully breed in sandy habitats comprising a mix of various soil particle sizes, rather than the fine sand dominant in artificial coastal habitats. Furthermore, given that flooding caused major nest failure, the construction of drainage channels in artificial habitats is also necessary. Therefore, when artificial habitats are constructed for Kentish plovers, it is of significant importance to consider vegetation cover and fine-scale substrate with drainage systems, in addition to simply providing exposed sandy habitats.

Given our findings, it can be reasonably deduced that the safeguarding of nesting habitats in artificial coastal areas for Kentish plovers may have a beneficial impact on the breeding habits of other shorebirds. As previous studies have demonstrated [23, 30], the nesting sites of Kentish plovers have been observed to be located in proximity to those of other shorebirds as Little terns and Eurasian oystercatchers, and we observed it in the Saemangeum. This indicated that if conservation and restoration efforts were conducted on artificial nesting sites for Kentish plovers, they would also provide breeding habitats other shorebirds, despite the anthropogenic origin of the locations. Thus, we suggest that the conservation of breeding habitats for Kentish plovers can provide benefits for the protection of other shorebird species as the previous study elucidated [21].

Even though the Saemangeum reclaimed lands are constructed for human use, our findings suggest that these lands can play a vital role as breeding habitats for a variety of shorebirds, in addition to Kentish plovers. Consequently, this insight acquired from the preceding international projects can inform future research, conservation, and restoration projects to apply the findings from Saemangeum to other regions. For instance, the importance of sand composition and vegetation management was emphasized by global conservation and restoration projects. Together with sand and vegetation management, the Netherlands conducted the Zand motor [60] and Hondsbossche dunes [61] projects, and the USA performed the Tijuana estuary restoration [62] and Delaware Bay [63] projects to conserve and restore the coastal line. In this study, we demonstrated that this effort could apply in Saemangeum reclaimed lands, considering the observed habitat characteristics. This approach ensures that restoration efforts will benefit the worldwide coastal habitats of various shorebird species, in addition to Kentish plovers. Furthermore, our recommendation for fine-scale substrate management and vegetation cover has the potential to be adapted to other reclaimed coastal areas worldwide, where similar environmental challenges are encountered.

In the meticulous modelling of the artificial habitats of Kentish plovers, our prediction and recommendation are based on flexibility rather than precise estimation, as would be the case in a generalized linear model (GLM). We provided their optimal habitat characteristics based on the primary preferences of individuals in Saemangeum, with likelihood and possibility in BN models. In particular, "uncertainty" is one of the most significant concepts in BN models [64]. This concept is distinguished from other models based on Neyman-Pearson frameworks in that it does not set strict assumptions and does not exclude variability as errors or outliers. However, BN can overcome this issue and can be a useful tool for interpreting uncertain ecological characteristics through conditional probabilities. Due to this flexibility, BN models can account for ecosystems in dynamic and stochastic perspectives without prematurely missing factors. For example, BN models can be used to evaluate the effect of substrate variability or unmeasured relationships and can provide well-founded explanations to flexibly understand on the habitats of animals in fine scales. This can provide BN with a valuable tool to make a plan for habitat conservation and restoration within dynamic ecological systems. Thus, considering that concept in addition to the wide variation as various substrate preferences and trade-offs with vegetation of nesting sites of Kentish plovers, the construction of suitable and adequate artificial habitats for Kentish plovers necessitates a flexible approach that considers the prevailing environmental and ecological conditions.

**Future studies regarding nesting sites of Kentish plovers**

Our study describes the association of substrate and vegetation with the nesting habits of Kentish plovers; however, more studies are necessary to elucidate the influence of other factors, which we observed, can affect the nesting habits of plovers. Firstly, the effect of footprints of other animals and humans on the nesting of Kentish plovers should be studied in the habitat. In the previous study, the successful nests of Kentish plovers were positioned at a farther distance from the footprints of mammals than unsuccessful ones [29]. Because the one of primary causes of nest failure in plover breeding is egg depredation by terrestrial animals [42]. On the effect of human footprints, our observations yielded intriguing insights into relationships with the nest site selection of Kentish plovers. During our field surveys, we frequently walked on arid and exposed riverbeds to observe the populations of this species and then unintentionally made the subsequent series of footprints on the riverbeds. After several days, a pair of Kentish plovers selected one of our footprints as the site for their nesting (Fig F in S1 File). Thus, we propose that human footprints may exert an anthropogenic impact on the nesting habits of Kentish plovers with the necessity of investigating this potential effect before the implementation of conservation strategies for their habitats. Secondly, regional differences in habitats must be studied to determine whether they affect nest site selection and nest success of Kentish plovers. Because we revealed that local differences in the Saemangeum can affect soil properties and can link with the nest success of this species. Kentish plovers are distributed widely from North Africa and Europe to East Asia [65]. They also inhabit various coastal environments, including sandy beaches [66], sandy barrier islands [30], rocky coasts [26, 53], saltpans [67], estuaries [52], and reclaimed coastal lands [69]. Like piping plovers [36], the relationship of their nesting sites with regional differences can have a significant impact. In addition, when we first conducted BN models excluding location variables, the results demonstrated higher AUC values in the nest site selection (0.97) and nest fate (1.00) models. However, present models incorporating location variables exhibited diminished AUC values. This suggests that investigating the localities of nesting sites is important for this species. Hence, future studies could benefit from cross-regional collaborations that compare habitat preferences across different populations of Kentish plovers or related species. The implementation of such collaborative endeavours would facilitate the development of more generalized conservation strategies for shorebird habitats on a global scale. Lastly, based on a sufficient dataset to conduct models and statistics, our study already offers valuable insights into the nesting site preference and nest success of Kentish plovers in artificial coastal habitats. However, to gain a comprehensive understanding of the effect of gradual coastal environmental changes on their breeding behaviours, further investigations based on a long-term dataset are necessary. Over time, the characteristics of coastal environmental factors, including vegetation cover and substrate composition, should change. Such changes may result in alterations to the habitats of shorebirds. Thus, long-term monitoring and the implementation of suitable management plans be undertaken to ensure the consistent conservation of their habitats and breeding sites. Such endeavours will provide solutions for the conservation of shorebirds and contribute to the understanding of the habitat dynamics influenced by anthropogenic impacts.

**Conclusion**

Our findings suggest that coastal artificial habitats, such as Saemangeum, may offer a potentially suitable environment for the breeding of shorebirds. However, despite that, this result does not support that it is okay to have consistent development in the area since the activity is gradually reducing the foraging and refuelling sites of shorebirds. Because of the loss of extensive tidal flats to reclamation projects in this region, human activities must be halted and managed in a manner that prioritizes ecological sustainability to support breeding populations of shorebirds. Now is the time to return the areas to shorebirds at least they can use there for breeding. Furthermore, our results provide quantitative information regarding the substrate and vegetation of the nesting sites of Kentish plovers. Understanding the association of substrate and vegetation with nest site selection and nest success of Kentish plovers requires comprehensive observations, approaches, and perspectives. Thus, this study suggests that all viewpoints must be fully considered based on comprehensive and multifaceted approaches to deeply understand the nesting habitats of shorebirds.

**References**

1. Lee JK, Chung OS, Park JY, Kim HJ, Hur WH, Kim SH, et al. Effects of the Saemangeum Reclamation Project on migratory shorebird staging in the Saemangeum and Geum Estuaries, South Korea. Bird Conserv Int. 2018;28: 238–250. doi: 10.1017/S0959270916000605.
2. Pakanen VM, Hongell H, Aikio S, Koivula K. Little Tern breeding success in artificial and natural habitats: Modelling population growth under uncertain vital rates. Popul Ecol. 2014;56: 581–591. doi: 10.1007/s10144-014-0446-1.
3. Jackson MV, Choi CY, Amano T, Estrella SM, Lei W, Moores N, et al. Navigating coasts of concrete: Pervasive use of artificial habitats by shorebirds in the Asia-Pacific. Biol Conserv. 2020;247: 108591. doi: 10.1016/j.biocon.2020.108591.
4. Gómez-Serrano MÁ. Dune restoration must consider species that need open and early successional dune habitats. Nat Ecol Evol. 2024;8: 1–2. doi: 10.1038/s41559-024-02441-5.
5. Sherfy MH, Stucker JH, Buhl DA. Selection of nest-site habitat by interior Least Terns in relation to sandbar construction. J Wildl Manag. 2012;76: 363–371. doi: 10.1002/jwmg.301.
6. Colwell MA, Haig SM. The population ecology and conservation of Charadrius plovers. Boca Raton, FL: CRC Press; 2019.
7. Manning AEE, White CM. Nest site selection by Mountain Plovers (*Charadrius montanus*) in a shrub-steppe habitat. West N Am Nat. 2001;61: 229–235. Available from: https://www.jstor.org/stable/41717126.
8. Nguyen LP, Nol E, Abraham KF. Nest success and habitat selection of the Semipalmated Plover on Akimiski Island, Nunavut. Wilson J Ornithol. 2003;115: 285–291. doi: 10.1676/03-044.
9. Vozabulová E, Sládeček M, Šálek M. Old habits in a new habitat: Breeding requirements of the Little Ringed Plover fit into intensively managed arable land. J Ornithol. 2020;161: 399–408. doi: 10.1007/s10336-019-01738-8.
10. Al-Moadhen MM, Clarke BG, Chen X. The permeability of composite soils. Environ Geotech. 2018;7: 478–490. doi: 10.1680/jenge.18.00030.
11. Eremin D, Eremina D. Influence of transport infrastructure on water permeability of soils of Western Siberia. In: IOP Conf Ser Earth Environ Sci. 2017;90: 012111. doi: 10.1088/1755-1315/90/1/012111.
12. Adnan MSG, Talchabhadel R, Nakagawa H, Hall JW. The potential of tidal river management for flood alleviation in south western Bangladesh. Sci Total Environ. 2020;731: 138747. doi: 10.1016/j.scitotenv.2020.138747.
13. Roche EA, Gratto-Trevor CL, Goossen JP, White CL. Flooding affects dispersal decisions in Piping Plovers (*Charadrius melodus*) in prairie Canada. Auk. 2012;129: 296–306. doi: 10.1525/auk.2012.11196.
14. Plaschke S, Bulla M, Cruz-López M, Gómez del Ángel S, Küpper C. Nest initiation and flooding in response to season and semi-lunar spring tides in a ground-nesting shorebird. Front Zool. 2019;16: 1–11. doi: 10.1186/s12983-019-0313-1.
15. Hood SL, Dinsmore SJ. The influence of habitat on nest survival of snowy and Wilson's plovers in the lower Laguna Madre region of Texas. Stud Avian Biol. 2007;34: 124–135.
16. Gómez-Serrano MÁ, López-López P. Nest site selection by Kentish Plover suggests a trade-off between nest-crypsis and predator detection strategies. PLoS ONE. 2014;9: e107121. doi: 10.1371/journal.pone.0107121.
17. Davis A. Breeding biology of the New Zealand shore plover (*Thinornis novaeseelandiae*). Notornis. 1994;41: 195–208.
18. Fraga RM, Amat JA. Breeding biology of a Kentish Plover (*Charadrius alexandrinus*) population in an inland saline lake. Ardeola. 1996;43: 69–85.
19. Cuccurullo G, Zwirner E. Notes on a new breeding site for the endangered Kentish Plover (*Charadrius alexandrinus*) in a touristic beach of Friuli-Venezia Giulia, Italy. Riv Ital Ornithol. 2023;93: 25–28. doi: 10.4081/rio.2023.648.
20. McDonald GC, Bede-Fazekas Á, Ivanov A, Crecco L, Székely T, Kosztolányi A. Landscape and climatic predictors of Kentish Plover (*Charadrius alexandrinus*) distributions throughout Kazakhstan. Ibis. 2022;164: 949–967. doi: 10.1111/ibi.13070.
21. Villalobos Perna P, Finizio M, Carranza ML, Innangi M, Bongiovanni SB, Andino N, et al. Anthropogenic and climate change-driven coastline dynamics will erode future nesting habitats of the Kentish Plover on the central Adriatic coast. Sci Rep. 2024;14: 23306. doi: 10.1038/s41598-024-74726-5.
22. Valle R, Scarton F. Nest-site selection and interspecific relationships of Oystercatcher (*Haematopus ostralegus*) on the Adriatic coast of Italy. Vogelwelt. 1996;117: 75–82.
23. Castro M, De la Cruz A, Martin-Sanjuan N, Pérez-Hurtado A. Kentish Plover (*Charadrius alexandrinus*) and Little Tern (*Sternula albifrons*) prefer shells for nesting: A field experiment. Avian Res. 2024;15: 100158. doi: 10.1016/j.avrs.2024.100158.
24. Kim WY, Bae SY, Oh SJ, Yoon HN, Lee JH, Paek WK, et al. Distribution of the Kentish Plover (*Charadrius alexandrinus*) based on the 3rd National Ecosystem Survey and its adequacy as a bioindicator. Korean J Environ Ecol. 2016;30: 155–164. (In Korean). doi: 10.13047/KJEE.2016.30.2.155.
25. Pietrelli L, Biondi M. Long-term reproduction data of Kentish Plover (*Charadrius alexandrinus*) along a Mediterranean coast. Bull Wader Study Gr. 2012;119: 114–119. doi: 10.1017/S0959270912000342.
26. Hanane S. Breeding ecology of Kentish Plovers (*Charadrius alexandrinus*) in rocky and sandy habitats of north-west Morocco (North Africa). Ostrich. 2011;82: 217–223. doi: 10.2989/00306525.2011.616681.
27. Que P, Chang Y, Eberhart-Phillips L, Liu Y, Székely T, Zhang Z. Low nest survival of a breeding shorebird in Bohai Bay, China. J Ornithol. 2015;156: 297–307. doi: 10.1007/s10336-014-1126-9.
28. El Malki S, Joulami L, El Mdari M, El Hamoumi R. Nest site characteristics and breeding biology of Kentish Plover in the saltpans of Sidi Moussa, Morocco. Wader Study. 2018;125: 107–114. doi: 10.18194/ws.00115.
29. Norte AC, Ramos JA. Nest-site selection and breeding biology of Kentish Plover (*Charadrius alexandrinus*) in sandy beaches of the Portuguese west coast. Ardeola. 2004;51: 255–268.
30. Scarton F, Cecconi G, Valle R. Use of dredge islands by a declining European shorebird, the Kentish Plover (*Charadrius alexandrinus*). Wetl Ecol Manag. 2013;21: 15–27. doi: 10.1007/s11273-012-9276-0.
31. Wentworth CK. A scale of grade and class terms for clastic sediments. J Geol. 1922;30: 377–392. doi: 10.1086/622910.
32. Blair TC, McPherson JG. Grain-size and textural classification of coarse sedimentary particles. J Sediment Res. 1999;69: 6–19. doi: 10.2110/jsr.69.6.
33. Uusitalo L. Advantages and challenges of Bayesian networks in environmental modelling. Ecol Model. 2007;203(3–4): 312–318. doi: 10.1016/j.ecolmodel.2006.11.033.
34. Gieder KD, Karpanty SM, Fraser JD, Catlin DH, Gutierrez BT, Plant NG, et al. A Bayesian network approach to predicting nest presence of the federally-threatened piping plover (*Charadrius melodus*) using barrier island features. Ecol Model. 2014;276: 38–50. doi: 10.1016/j.ecolmodel.2014.01.005.
35. Zeigler SL, Gutierrez BT, Sturdivant EJ, Catlin DH, Fraser JD, Hecht A, et al. Using a Bayesian network to understand the importance of coastal storms and undeveloped landscapes for the creation and maintenance of early successional habitat. PLoS ONE. 2019;14(7): e0209986. doi: 10.1371/journal.pone.0209986.
36. Zeigler SL, Gutierrez BT, Hecht A, Plant NG, Sturdivant EJ. Piping Plovers demonstrate regional differences in nesting habitat selection patterns along the US Atlantic coast. Ecosphere. 2021;12: e03418. doi: 10.1002/ecs2.3418.
37. Hur WH, Kim HJ, Nam HG, Hwang JW, Lee JY, Lee JY, et al. A study of bird population monitoring in different habitats. National Inst Biol Resour., Incheon, South Korea; 2019.
38. Lee JH, Kim IC, Lee SW, Son JJ, Jang JU, Sung HC. International importance of tidal flats in the Republic of Korea as shorebird stopover sites in the East Asian–Australasian flyway. Avian Conserv Ecol. 2023;18: 23. doi: 10.5751/ACE-02449-180123.
39. Koh CH, Ryu JS, Khim JS. The Saemangeum: History and controversy. J Korean Soc Mar Environ Energy. 2010;13: 327–334. Available from: https://koreascience.or.kr/article/JAKO201003949970740.page.
40. Lee SJ, Oh CH. Characteristics of vascular plants in Saemangeum reclaimed land. Proc Korean Soc Environ Ecol Conf. 2021;31: 4.
41. Mabee TJ, Wildman AM, Johnson CB. Using egg flotation and eggshell evidence to determine age and fate of Arctic shorebird nests. J Field Ornithol. 2006;77: 163–172. doi: 10.1111/j.1557-9263.2006.00037.x.
42. Colwell MA, Meyer JJ, Hardy MA, McAllister SE, Transou AN, Levalley RR, et al. Western Snowy Plovers (*Charadrius alexandrinus nivosus*) select nesting substrates that enhance egg crypsis and improve nest survival. Ibis. 2011;153: 303–311. doi: 10.1111/j.1474-919X.2011.01100.x.
43. Meireles RCD, Teixeira JPG, Solar R, Vasconcelos BNF, Fernandes RB, Lopes LE. Soil attributes drive nest-site selection by the campo miner (*Geositta poeciloptera*). PLoS ONE. 2018;13: e0192185. doi: 10.1371/journal.pone.0192185.
44. Kim WY, Kim IC, Choi GH, Sung HC. Comparison of nest building patterns in the family Charadriidae: Three species (*Charadrius alexandrinus, C. placidus, C. dubius*). Proc Korean Soc Environ Ecol. 2012;2: 226–229. (In Korean). Available from: https://db.koreascholar.com/Article/Detail/311032.
45. Kroetsch D, Wang C. Particle size distribution. In: Soil Sampling and Methods of Analysis, 2nd ed. Boca Raton, FL, USA: CRC Press; 2008. pp. 713–725.
46. Kassambara A, Mundt F. Factoextra: Extract and visualize the results of multivariate data analyses (R package version 1.0.7). 2020. Available from: https://CRAN.R-project.org/package=factoextra.
47. Scutari M, Denis JB. Bayesian Networks with Examples in R. Boca Raton, FL, USA: Chapman and Hall; 2014.
48. Langarizadeh M, Moghbeli F. Applying naive Bayesian networks to disease prediction: A systematic review. Acta Inform Med. 2016;24: 364–369. doi: 10.5455/aim.2016.24.364-369.
49. Marcot BG. Metrics for evaluating performance and uncertainty of Bayesian network models. Ecol Model. 2012;230: 50–62. doi: 10.1016/j.ecolmodel.2012.01.013.
50. R Core Team. R: A language and environment for statistical computing. Vienna, Austria: R Foundation for Statistical Computing; 2024. Available from: https://www.R-project.org.
51. Revelle WR. psych: Procedures for personality and psychological research. Northwestern University, Evanston, Illinois, USA; 2017. Available from: https://CRAN.R-project.org/package=psych.
52. Hong SB, Higashi S. Nesting site preference and hatching success of the Kentish Plover (*Charadrius alexandrinus*) in the Nakdong Estuary, Busan, Republic of Korea. J Ecol Environ. 2008;31: 201–206. doi: 10.5141/JEFB.2008.31.3.201.
53. Fojt E, Triplet P, Robert JC, Stillman RA. Comparison of the breeding habitats of Little Ringed Plover (*Charadrius dubius*) and Kentish Plover (*Charadrius alexandrinus*) on a shingle bed. Bird Study. 2000;47: 8–12. doi: 10.1080/00063650009461155.
54. Wald A. A method of estimating plane vulnerability based on damage of survivors. Stat Res Gr Columbia Univ. 1943;CRC 432.
55. Mangel M, Samaniego FJ. Abraham Wald's work on aircraft survivability. J Am Stat Assoc. 1984;79: 259–267. doi: 10.1080/01621459.1984.10478038.
56. Engel N, Végvári Z, Rice R, Kubelka V, Székely T. Incubating parents serve as visual cues to predators in Kentish Plover (*Charadrius alexandrinus*). PLoS ONE. 2020;15: e0236489. doi: 10.1371/journal.pone.0236489.
57. Kim JS, Kim JH, Chae HY, Hwang BY. Breeding ecology of the Kentish Plover (*Charadrius alexandrinus*) at Baramarae Beach, a special protected area in the Taeanhaean National Park. Korean J Ornithol. 2021;28: 49–58. (In Korean). doi: 10.30980/kjo.2021.12.28.2.49.
58. Amat JA, Masero JA. Predation risk on incubating adults constrains the choice of thermally favourable nest sites in a plover. Anim Behav. 2004;67: 293–300. doi: 10.1016/j.anbehav.2003.06.014.
59. Gómez J, Liñán-Cembrano G, Ramo C, Castro M, Pérez-Hurtado A, Amat JA. Does the use of nest materials in a ground-nesting bird result from a compromise between the risk of egg overheating and camouflage? Biol Open. 2019;8: bio042648. doi: 10.1242/bio.042648.
60. Huisman BJA, Wijsman JWM, Arens SM, Vertegaal CTM, Van der Valk L, Van Donk SC, et al. 10-years evaluation of the Sand Motor: Results of the Monitoring- and Evaluation Program (MEP) for the period 2011 to 2021 (Report No. 11201431-003-ZKS-0014). Rijkswaterstaat, The Hague, Netherlands.
61. Bechauf R, Cutler E, Gouett M, Guzzetti M. Sustainable Asset Valuation (SAVi) of nature-based coastal protection in the Netherlands: An economic valuation of the contribution of the Hondsbossche Dunes sand nourishment to climate adaptation and local development. Int Inst Sustain Dev. 2021.
62. Powell AN. Up, down, or stable: Populations of endangered birds in beach and estuarine areas in southern California. Plan Biodivers Bring Res Manag Together. 2005;17: 1–17.
63. Smith JA, Niles LJ, Hafner S, Modjeski A, Dillingham T. Beach restoration improves habitat quality for American horseshoe crabs and shorebirds in the Delaware Bay, USA. Mar Ecol Prog Ser. 2020;645: 91–107. doi: 10.3354/meps13371.
64. Marcot BG, Hanea AM. What is an optimal value of k in k-fold cross-validation in discrete Bayesian network analysis? Comput Stat. 2021;36: 2009–2031. doi: 10.1007/s00180-020-00999-9.
65. IUCN. The IUCN Red List of Threatened Species. 2024. Available from: https://www.iucnredlist.org.
66. McDonald GC, Engel N, Ratão SS, Székely T, Kosztolányi A. The impact of social structure on breeding strategies in an island bird. Sci Rep. 2020;10: 13872. doi: 10.1038/s41598-020-70595-w.
67. Rocha AD, Fonseca D, Masero JA, Ramos JA. Coastal saltpans are a good alternative breeding habitat for Kentish Plover (*Charadrius alexandrinus*) when umbrella species are present. J Avian Biol. 2016;47: 824–833. doi: 10.1111/jav.00883.
68. Toral GM, Figuerola J. Nest success of Black-winged Stilt (*Himantopus himantopus*) and Kentish Plover (*Charadrius alexandrinus*) in rice fields, southwest Spain. Ardea. 2012;100: 29–36. doi: 10.5253/078.100.0106.

**Acknowledgments**

We thank the Korea Rural Community Corporation and Saemangeum Project Office for permission to collect field data. We appreciate the assistance provided by the lab members of the Eco-Behav lab at Chonnam National University. We are thankful to Prof. Yul Roh and Dr. Sara L. Zeigler for providing valuable advice regarding the geological analysis and Bayesian network modelling process. We also thank Yu-Jun Kim for their assistance in conducting the field surveys.

**Data availability statement**

The datasets generated and/or analyzed during the current study are available from the corresponding author on reasonable request.

**Competing interests**

The authors declare that they have no conflicts of interest.

**Consent to participate**

Not applicable

**Author contributions**

**Conceptualization:** Dong-Yun Lee

**Data curation:** Dong-Yun Lee, Jong-Ju Son

**Formal analysis:** Dong-Yun Lee, Jong-Ju Son

**Investigation:** Dong-Yun Lee, Seung-Jun Oh

**Methodology:** Dong-Yun Lee

**Software:** Dong-Yun Lee

**Validation:** Dong-Yun Lee, Ju-Hyun Lee

**Visualization:** Dong-Yun Lee

**Writing – Original Draft Preparation:** Dong-Yun Lee

**Writing – Review & Editing:** Ju-Hyun Lee, Ha-Cheol Sung

**Project administration:** Ha-Cheol Sung

**Funding acquisition:** Ha-Cheol Sung

**Supervision:** Ha-Cheol Sung

**Figures and their captions**

**Fig 1.** Study areas in Samanguem, South Korea. (A) Mangyeong estuary, Gunsan City, Jellabuk-do Province (35°52'N, 126°40'E). (B) Dongjin estuary, Buan County, Jellabuk-do Province (35°48'N, 126°38'E). We found 60 nests in Mangyeong estuary and 35 nests in Donjin estuary during the 2020 breeding.

**Fig 2.** Bayesian network model of nest site selection of Kentish plovers (95 nest site and 190 non-nest site data). We constructed this model using eight environmental factors in 1-m^2^ quadrat photos. Arrows denote the direction of conditional dependencies (arc). Boxes indicate nodes (black bars in each node represent the probability of each category in a variable). DV = dry vegetation, TV = total vegetation, LV = live vegetation, A = absence, P = presence

**Fig 3.** Cluster analysis of cover rate in 1-m2 quadrat and soil particle size. (A) In type A, sand cover was at the highest rate, followed by dry vegetation. In type B, dry vegetation was at the highest cover rate, followed by sand. (B) In types 1 and 2, fine sand was the main component, followed by very fine sand. In type 3, very fine sand was the main component, followed by fine sand.

**Fig 4.** Bayesian network model of nest fate of Kentish plovers prepared using the data of 61 successful nests and 34 unsuccessful nests. We used four environmental factors. NVD = distance between a nest and the nearest vegetation, NWD = distance between a nest and water source

**Fig 5.** Principal Component Analysis (PCA) of nest fail reasons and environmental factors. In these graphs, flooding events were largely related to soil particle sizes. However, unknown reason showed the relationships with shell and vegetation covers.

**Tables**

Table 1. Variables used in the nest site selection model. All variables were measured in the 1-m^2^ quadrat of nest sites and non-nest sites.

| **Variable** | **Discretizing method** | **Definition** |
| --- | --- | --- |
| Dry vegetation | Median and quartiles | Dead vegetation, such as dry twigs and grass |
| Live vegetation | Median and quartiles | Live vegetation when researchers took the picture of the nest |
| Total vegetation | Median and quartiles | All vegetation, whether dry or live |
| Shell | Median and quartiles | Covered with seashells of bivalves or gastropods |
| Object | Presence or absence | Other objects, such as vinyl and plastic |
| Sand | Median and quartiles | Sand particle size < 2 mm |
| Granule + pebble | Presence or absence | 2 mm < Granule particle size < 4 mm 4 mm < Pebble particle size < 64 mm (Granules or pebbles of these sizes were less in our study. Thus, we counted the grains in these two size categories together.) |
| Cobble | Presence or absence | 64 mm < Cobble particle size < 256 mm |

Table 2. Variables used in the nest fate model. These variables were measured in each nest
site.

| **Variable** | **Discretizing method** | **Definition** |
| --- | --- | --- |
| Distance between a nest and the nearest vegetation | Median and quartiles | Distance between a nest and the nearest vegetation (directly measured in the field) |
| Distance between a nest and water source | Median and quartiles | Distance between a nest and the nearest water source  (measured using GPS and satellite map [Kakao map]) |
| Nest group based on cover rate on nests in 1-m² quadrat | Clustering | Cover type within 1 m^2^ of the nest site (dry vegetation, live vegetation, shell, sand, granule + pebble, cobble, and object) |
| Nest group based on soil particle size | Clustering | Type of soil particle in nest site (gravel: > 2 mm, very coarse sand: 1–2 mm, coarse sand: 0.5–1 mm, medium sand: 0.25–0.5 mm, fine sand: 0.125–0.25 mm, very fine sand: 0.063–0.125 mm, and mud: 0.063 mm) |
