# Supplementary material for: Estimating nesting habitat characteristics for the Kentish plover (Anarhynchus alexandrinus) with the effect of substrate and vegetation using a Bayesian network approach
Source: PLoS One. 2025 Jun 11;20(6):e0325750. doi: 10.1371/journal.pone.0325750 (PMC12157252; doi:10.1371/journal.pone.0325750)
Supplement: Attachment — Submitted filename: Resonses to the Reviewers - Plos One.docx [file pone.0325750.s007.docx]

**Responses to the Reviewers’ comments**

We would like to express our gratitude for your review and the detailed comments you provided. We have modified the manuscript according to your instructions and attempted to resolve all issues you mentioned. In this response, we will address each of your concerns point by point.

**1. Regarding English writing editing,**

**To improve clarity and readability in our manuscript, the revised manuscript were professionally edited for English language by EssayReview (www.essayreview.co.kr).**

**2. Regarding the title and keywords**

We revised the titles by deleting "reclaimed land" and "geological criteria." We also wrote new keywords that are not repeated in the title or abstract.

**Changed lines 1–3:** from “**Full title: Estimating the characteristics of nesting habitat for the Kentish Plover (*Anarhynchus alexandrinus*) in artificially reclaimed lands with the effect of substrate and vegetation based on a Bayesian network approach and geological criteria**” to “**Full title: Estimating nesting habitat characteristics for the Kentish Plover (*Anarhynchus alexandrinus*) with the effect of substrate and vegetation using a Bayesian network approach**”

**Changed lines 5–6:** from “**Short title: Estimating nest habitat traits of Kentish Plover in reclaimed lands using Bayesian network and geological criteria**” to “**Short title: Estimating Kentish Plover nesting habitat characteristics using a Bayesian network model**”

**Changed lines 40–41:** From “Bayesian network model, Kentish Plover, Nesting habitat, Reclaimed land, Shorebird conservation, Substrate particle size, Vegetation cover” **To** “Bayesian modelling, Nest-site selection, Nest success, Shorebird nesting, Shorebird conservation, Coastal management”

**3. Regarding Introduction section**

We sought to reduce the repetition of explanations on the nesting patterns of plovers. In addition, we moved the detailed explanation of nest presence/absence and success/failure from the Introduction to Materials and Methods section. In addition, the explanation of geological criteria is placed after the objectives.

**Change lines 45–47:** **From** “To protect shorebirds, considering how to return artificial coastal areas, which have already been developed, to them is necessary for restoration and conservation projects.” **To** “To ensure the conservation of shorebird populations, it is essential to consider strategies restoring developed artificial coastal areas and returning them to their natural state.”

**Deleted** “Ground environmental factors like substrate and vegetation, in particular, play a significant role in nest site selection and nest success of ground-nesting shorebirds [6].” around line 55

**Added lines 71–77:** “Therefore, this study aimed to investigate the microhabitat characteristics of Kentish plovers in coastal areas of South Korea, examining their relationships with substrate composition [18–22] and vegetation cover [23]. Additionally, we examined other environmental factors, including shell cover, the presence of artificial objects, and distance from water sources to determine whether these variables have a greater effect on nest site selection and nest success than substrate and vegetation.”

**Deleted** “First, plovers are known to select a site for nesting depending on specific substrate cover particle sizes [8]. Body and egg size stones are useful for nest camouflage and crypsis [9]. On the other hand, small substrates differently affect the nesting of plovers. For example, the distribution of soil particles (sand and mud) correlates with soil permeability and porosity [10]. Decreased soil permeability and porosity slow underground water absorption [11] and can cause more flooding in an area [12]. Considering that flooding is a major reason for nest failure, the distribution of soil particles in habitats can influence the nesting sites of plovers [13–14]. Second, vegetation cover in habitats is associated with nest site selection and nest success of plovers. Most plovers prefer areas with reduced vegetation cover for nesting [15] because high vegetation cover hinders incubating the ability of plovers to detect approaching predators [16]. Nevertheless, the presence of vegetation in nesting sites occasionally improves the nest success of plovers. Because, incubating plovers may use vegetation to conceal their offspring from detection by predators, although they may also be vulnerable to attacks because the dense vegetation can create visual obstacles preventing the detection of approaching predators [17].” around line 60

**Deleted** “In particular, in the coastal habitats, substrate and vegetation are common environmental factors that show complicated relationships with the nesting of Kentish plovers. First, coasts of various substrate types influence nest site selection and nest success of Kentish plovers. Most of them prefer sandy coasts to areas covered by rocks and stones for nesting [25]. However, Kentish plovers in some regions select nesting sites in rocky coasts [26]. On the fine-scale substrate of nesting sites, mud substrate was found to decrease the nest survival rate of Kentish plovers in Bohai Bay, China [27]. Second, the vegetation in habitats is complexly related to the nesting of Kentish plovers. These plovers have been known to avoid nesting in areas with vegetation cover [28], because it interferes with the ability of parent birds in detecting predators approaching their nests [29]. This can have a negative impact on their breeding [6]. However, high vegetation cover in a nest site can increase the probability of nest survival because an incubating bird can hide from predators in the vegetation, thus contributing to nest success [16]. Thus, vegetation cover rate and presence/absence have more significant influence on their nesting than vegetation species composition [30].” around line 76

**Changed lines 83–84:** **From** “In this study, we tried to follow geological criteria in the substrate analysis of plover habitats.” **To** “Standardized classification systems for substrate particles, based on geological criteria, are instrumental in elucidating the substrate characteristics of plover nesting habitats.”

**4. Regarding the reason of research site selection**

We wrote about why we chose Saemangeum as a research site. Saemangeum is almost in the middle of the Korean tidal flats, between the Seocheon and Gochang tidal flats, which are UNESCO World Natural Heritage Sites.

**Moved and changed lines 100–108 from Introduction to Materials and Methods**: **From** “In this research, we chose an appropriate field survey area carefully. To provide information the breeding of Kentish plovers in artificial habitats, we had to conduct field surveys in artificial locations. We selected the locations that shorebirds, including Kentish plovers, use as habitats. Next, we needed to gain insight into the diverse relationships between Kentish plovers and their surrounding substrates and vegetation for a comprehensive range of data pertaining to the nesting patterns. Had we selected multiple regions, we would have been unable to ascertain whether the observed preferences of Kentish plovers were attributable to regional differences or environmental factors. Therefore, we selected a specific coastal and artificial location with diverse substrate and vegetative conditions.” **To** “For the study, an appropriate field survey area was carefully selected. To provide insight into the breeding ecology of Kentish plovers in artificial habitats, field surveys were conducted in artificial coastal habitats. We selected locations known to be used by shorebirds, including Kentish plovers, as habitats. Furthermore, to gain insights into the diverse relationship between Kentish plovers and their surrounding substrate and vegetation, a single coastal and artificial location with diverse substrate and vegetative conditions was selected. If multiple regions had been included in the study, it would have been difficult to ascertain whether observed nesting preferences were due to regional differences or environmental factors. Hence, a single location with diverse conditions was selected.”

**Added lines 162–170:** “Saemangeum is located between the Seocheon and Gochang tidal flats, which are significant habitats for shorebirds and have been designated as UNESCO World Natural Heritage Sites. This further underscores the ecological significance of this region.”

**Deleted** “Developments in Saemangeum have demonstrated a negative correlation with avian populations” around line 114

**5. Regarding the descriptions of used materials**

We included the information about the camera taking a 1-meter square photograph. We also wrote the information about mesh sizes using a standard sieve set.

**Added line 142:** “using the rear camera of a Samsung Galaxy S9 camera (12 MP, OIS, f/1.5–2.4 dual aperture)”

**Added line 165–167:** “using a standard sieve set with ASTM mesh sizes: #10 (2.00 mm), #18 (1.00 mm), #35 (500 µm), #65 (250 µm), #120 (125 µm), and #230 (63 µm).”

**6. Regarding the explanation of Bayesian network model**

The explanation of the benefits of the BN model has been moved from the Introduction to the Materials and Methods section. Additionally, we included the assumptions related to this model in the Materials and Methods section. We also added explanations of the limitations of BN models in the Discussion section.

**Moved and changed lines 177–194 from Introduction to Materials and Methods: From** “We developed models to predict nest presence/absence and success/failure depending on environmental factors, such as substrate type (particle size) and vegetation cover. First, we examined the substrate characteristics of nest sites to determine the particle size that has the greatest influence on nest site selection and nest success of Kentish plovers. We described differences in the range of particle sizes observed between nest/non-nest sites and successful/unsuccessful nest sites. We then needed to examine the effect of particle size based on a finer scale on the nest success of Kentish plovers. We thus demonstrated differences in the rate of each particle size between successful and unsuccessful nest sites. Second, we investigated the impact of vegetation cover on nest site selection and nest success of Kentish plovers. We have avoided providing a direct answer to how vegetation affects nest site selection and nest success of Kentish plovers because of the complicated associations. Rather, we described the differences in vegetation cover at nest and non-nest sites as well as successful and unsuccessful nest sites using modelling process.

We employed a Bayesian network (BN) modeling approach. This is because BN models offer several advantages, including direct graphical representation, structure learning, and integration of diverse data sources [36]. This method has been successfully applied in previous studies on the piping plover (*Charadrius melodus*) to identify key habitat factors and support conservation planning [37–39].

The present study assumed the BN model. First, the prediction of the BN model was based on conditional possibility with the relationships between pre- and post-events. Next, the BN model structures of ecological research were constructed by algorithm (e.g., Grow-Shrink, Hill-Climbing, Tabu, etc.) and ecological information with enough sample sizes.”

**To**

“We developed models to predict nest presence or absence and success or failure based on environmental factors, such as substrate type (particle size) and vegetation cover. First, the substrate characteristics of nest sites were examined to identify particle sizes with the most significant influence on nest site selection and nest success in Kentish plovers. Furthermore, differences in the distribution of particle sizes between nest and non-nest sites, as well as successful and unsuccessful nest sites were assessed. Onto evaluate the influence of finer-scale variation, the proportion of each particle size category was analyzed in relation to nest success. Second, we investigated the impact of vegetation cover on nest site selection and nest success of Kentish plovers. Given the complex relationships involved, we refrained from drawing direct conclusions regarding the effects of vegetation. Instead, we described the differences in vegetation cover between nest and non-nest sites, as well as between successful and unsuccessful nest sites, using a modelling approach.

We employed a Bayesian network (BN) modelling approach, which offers several advantages, including direct graphical representation, structural learning, and the integration of diverse data sources [36]. This method has been successfully applied in previous studies on the piping plover (Charadrius melodus) to identify key habitat factors and inform conservation planning [37–39].

The present study used the BN model. First, the predictions of the BN model were based on conditional possibilities, reflecting the relationships between pre- and post-events. Next, the BN model structures used in ecological research were constructed using various algorithms (e.g., Grow-Shrink, Hill-Climbing, and Tabu.) and ecological information with sufficiently large sample sizes.”

**Deleted** “This can provide BN with a valuable tool to make a plan for habitat conservation and restoration within dynamic ecological systems.” around line 453

**Deleted** “We provided their optimal habitat characteristics based on the primary preferences of individuals in Saemangeum, with likelihood and possibility in BN models.” around line 445

**Added line: 462–476:** “Unlike GLMs, BN models allowed us to identify the trade-off strategies and the complex effects of soil particle size in the nesting patterns of Kentish plovers by analyzing conditional possibility and causality among variables. Our models also provide insights into the optimal habitat characteristics of this species based on the primary preferences of individuals in Saemangeum. Rather than promoting a one-size-fits-all approach, our findings suggest habitat management strategies that balance vegetation cover and mixed soil types to establish conservation and restoration plans. However, the construction of BN models is inherently dependent on the sample size and a priori knowledge. These models can produce skewed results when working with an inadequate dataset or when the basic information is incorrect. Given the limited sample size of unsuccessful nests, we used PCA to examine the relationships between nest failure causes and environmental factors. Furthermore, in addition to constructing BN models, it is necessary to discretize continuous variables into categorical factors. Consequently, to better assess variable tendencies, we recommend employing additional modelling techniques based on the Neyman-Pearson framework, such as GLMs, in conjunction with BN models.”

**Deleted** “considering that concept in addition to the wide variation as various substrate preferences and trade-offs with vegetation of nesting sites of Kentish plovers, the construction of suitable and adequate artificial habitats for Kentish plovers necessitates a flexible approach that considers the prevailing environmental and ecological conditions.” around line 468

**7. Regarding the Ethic statement**

We included a more detailed explanation of minimizing our disturbance for breeding individuals in our field surveys with the model number of the binoculars and field scope

**Changed and added lines 250–261:** “The first author completed a formal training program on animal welfare and ethical research practices (Certificate No. CNU IACUC-2020-107), issued by Chonnam National University (CNU) and the CNU Laboratory Animal Research Centre, to ensure adherence to ethical standards during the research process. No approval from a research ethics committee was required for this study, as we no birds were captured, handled, or manipulated. Despite not requiring formal approval, all data were collected using non-invasive methods in strict compliance with the current laws and regulations of South Korea to minimize disturbance to wildlife. A safe distance was maintained during observations of incubating individuals using binoculars (Nikon Prostaff 8 × 42) and a scope (Nikon Prostaff 5 field scope 82-A & SEP-20–60) to prevent interference. Nesting data collection was initiated only in the absence of incubating birds on the target nest, and all data was collected within a five-minute period to minimize disturbances.”

**8. Regarding Discussion section**

We tried to reduce the repetition. Especially, we merged “Recommendation and importance on the construction of artificial coastal breeding habitats for Kentish plovers” and “Future studies regarding nesting sites of Kentish plovers” into “Recommendation with the role of Bayesian Network models on the construction of artificial coastal breeding habitats for Kentish plovers and future studies” with three paragraphs. Furthermore, we linked the BN modelling characteristics with our results and explained the reduction of AUC values in more detail.

**Deleted** “We conducted a comprehensive study to provide quantitative information and estimate the characteristics of nesting sites of Kentish plovers in Saemangeum reclaimed lands, with the objective to contribute to the conservation and restoration of shorebird breeding habitats. In particular, focusing on substrate type and vegetation cover in coastal habitats, we also performed a multifaceted study to clarify the role and association of ground environmental factors with nesting sites of the Kentish Plover.” around line 343

**Added lines 351–349:** “Our BN models, in particular, the importance of maintaining a balance among environmental factors at breeding sites, as this affects both nest site selection and nest success. In addition, flooding, the main cause of nest failure, was associated with specific soil particle sizes. These findings suggest the need for coastal habitat management strategies that consider the balance between substrate types and vegetation cover to ensure the breeding success of Kentish plovers.”

**Changed lines 436: from** “**Recommendation and importance on the construction of artificial coastal breeding habitats for Kentish plovers**” **to** “**Recommendations for artificial coastal breeding habitats and future studies**”

**Deleted** “We therefore examined the optimal characteristics of their nesting site based on our model predictions. Prior to the initiation of conservation and restoration projects, we would like to raise the need to consider” around line 429

**Added line 429:** “The BN modelling results suggest”

**Deleted** “less than 90% sand cover and can successfully breed in sandy habitats comprising” around line 430

**Deleted** “Therefore, when artificial habitats are constructed for Kentish plovers, it is of significant importance to consider vegetation cover and fine-scale substrate with drainage systems, in addition to simply providing exposed sandy habitats.” around line 432

**Added lines 432–433:** “Furthermore, as reported in previous studies and observed in this study, the nesting sites of Kentish plovers are located near those of other shorebirds [16, 23],”

**Deleted** “Given our findings, it can be reasonably deduced that” around line 437

**Deleted** “As previous studies have demonstrated [23, 30], the nesting sites of Kentish plovers have been observed to locate in proximity to those of other shorebirds as little terns and Eurasian oystercatchers, and we observed it in the Saemangeum. This indicated that if conservation and restoration efforts were conducted on artificial nesting sites for Kentish plovers, they would also provide breeding habitats other shorebirds, despite the anthropogenic origin of the locations. Thus, we suggest that the conservation of breeding habitats for Kentish plovers can provide benefits for the protection of other shorebird species as the previous study elucidated [21].

Even though the Saemangeum reclaimed lands are constructed for human use, our findings suggest that these lands can play a vital role as breeding habitats for a variety of shorebirds, in addition to Kentish plovers.” around line 438

**Deleted** “In this study, we demonstrated that this effort could apply in Saemangeum reclaimed lands, considering the observed habitat characteristics. This approach ensures that restoration efforts will benefit the worldwide coastal habitats of various shorebird species, in addition to Kentish plovers. Furthermore, our recommendation for fine-scale substrate management and vegetation cover has the potential to be adapted to other reclaimed coastal areas worldwide, where similar environmental challenges are encountered.”
around line 442

**Deleted** “Because, the one of primary cause of nest failure in plover breeding is” around line 474

**Deleted** “On the effect of human footprints, our observations yielded intriguing insights into relationships with the nest site selection of Kentish plovers. During our field surveys, we frequently walked on arid and exposed riverbeds to observe the populations of this species and then unintentionally made the subsequent series of our footprints on the riverbeds. After several days,” around line 475

**Deleted** “Like piping plovers [36], the relationship of their nesting sites with regional differences can have a significant impact. In addition, when we firstly conducted” around line 485

**Added lines 495–498:** “This indicated that, except for location variables, our previous models had likely been overfitted, resulting in shown high AUC values [67]. The reduction in AUC values after including location variables suggests that our models became more generalized and less prone to overfitting.”

**Added line 494:** “in a global scale.”

**Deleted** “The implementation of such collaborative endeavors would facilitate the development of more generalized conservation strategies for shorebird habitats on a global scale. Lastly, based on a sufficient dataset to conduct models and statistics, our study already offers valuable insights into the nesting site preference and nest success of Kentish plovers in artificial coastal habitats. However,” around line 494

**9. Regarding the conclusion**

We tried to reflect both reviewers' opposing opinions. We rewrote the conclusion to encompass both global perspectives and the importance of Saemangeum conservation.

**Deleted** “despite that, this result does not support that it is okay to consistent development in the area since the activity is” around line 507

**Added lines 514–515:** “ongoing shorebird habitat modifications due to land reclamation projects have”

**Deleted** “Furthermore, our results provide quantitative information regarding substrate and vegetation of the nesting sites of Kentish plovers. Understanding the association of substrate and vegetation with nest site selection and nest success of Kentish plovers requires comprehensive observations, approaches, and perspectives. Thus, this study suggests that all viewpoints must be fully considered based on comprehensive and multifaceted approaches to deeply understand the nesting habitats of shorebirds.” around line 512

**Added lines 519–528:** “The insights gained from this study have significant conservation implications for Saemangeum, where shorebird habitats remain under persistent threat. A scientific approach is essential for mitigating habitat loss and enhancing the capacity of these artificial landscapes to support breeding populations. Our findings underscore the necessity of targeted habitat management that balances substrate composition, vegetation cover, and nest success in reclaimed areas. Moreover, the treat to coastal area extends beyond Saemangeum, as developments worldwide threaten shorebird habitats. Understanding the relationship between environmental factors such as substrate composition and vegetation is the primary step in obtaining quantitative to inform for conservation and restoration efforts in coastal environments.”

**10. Regarding the map of study area in Figure 1,**

Considering CC BY 4.0 on the map data, we have replaced the base maps using national boundary shapefiles provided by the National Geographic Information Institute (NGII), Republic of Korea. All data used are available under KOGL Type 1 license which is equivalent with the Creative Commons Attribution License (CC BY 4.0). Both sources allow commercial reuse and meet the license requirements of PLOS ONE. In particular, NGII map data is open free use public source. We contacted the official directly to confirm that the shapefiles were not copyrighted and that we could freely and without restriction utilize them for academic purposes. However, due to Korean civil service regulations, they were unable to provide us with the files that included the official's name and signature, which was required by PLOS ONE. Hence, we required them to know the related webpages about their opinion, but NGII only has a simple webpage about this information in Korean, which we included it below. Furthermore, we manually delineated each area's colors on the maps.

🡪 National Geographic Information Institute (NGII). (2020). Korea Administrative Boundary Shapefiles. Retrieved from https://www.ngii.go.kr/ (Open data under KOGL Type 1 license).

Information about KOGL Type 1 license (in Korean): https://www.kogl.or.kr/info/license.do

**Changed line 764: from** “Study areas in Samanguem, South Korea. (A) Mangyeong estuary, Gunsan City, Jellabuk-do Province (35°52'N, 126°40'E). (B) Dongjin estuary, Buan County, Jellabuk-do Province (35°48'N, 126°38'E). We found 60 nests in Mangyeong estuary and 35 nests in Donjin estuary during the 2020 breeding.” **To**

“Study areas in Saemangeum, South Korea. Four colours indicated the characteristics of each area (Grey: anthropogenic areas like residential areas, agriculture areas, dykes, and bridges; Green: natural areas; Light brown: reclaimed lands by Saemangeum projects; Dark brown: the research area of this study in reclaimed lands by Saemangeum projects). We conducted field surveys on (a) Mangyeong estuary, Gunsan City, Jellabuk-do Province (35°52'N, 126°40'E). (b) Dongjin estuary, Buan County, Jellabuk-do Province (35°48'N, 126°38'E) in the Saemangeum area (c). A total of 60 nests were detected in the Mangyeong estuary and 35 nests in the Dongjin estuary during the 2020 breeding season. The base map of South Korea indicates the location of our study areas with the red star (c). We obtained the open source base map data was obtained from the National Geographic Information Institute, South Korea (https://www.ngii.go.kr/kor/main.do). Administrative boundary shapefiles were derived from the National Geographic Information Institute (NGII 2020), Republic of Korea, open under KOGL Type 1 license (which permits commercial reuse and redistribution with attribution).

”

**11. Regarding the citations and captions of figures**

We cited all of the figures in our text. We wrote more detailed explanations for Figure 8 (previous Figure 5) in the text and caption.

**Added line 336–339:** “in the graph of PC1 and PC2 (Fig 8A), but among the soil particle sizes, coarse, medium, and fine sand only showed relationships with flooding in the graph of PC1 and PC2 (Fig 8B).Unknown did with most of the cover variables including vegetation and shell covers in both graphs (Fig 8).”

**Added line 814–815:** “In particular, coarse, medium, and fine sand showed the relationships with flooding in both graphs.”

**12. Regarding the tables and figures in supplementary**

We moved five tables from File S1 to the manuscript. Their number from Table 3 to 7. On the figures, if you include all the supplementary figures from our BN modeling training and the inverse estimation, the manuscript would become overly lengthy and confusing, so we aimed to condense it and organize those figures. However, we encountered issues with small text, leading us to place some of those figures in the appendix. Except for those figures, everything else, like the photos of nests, has been relocated to the main text.

**Added lines 786–789:** “**Fig 2.** Examples of substrate particle size analysis. (a) A soil sample was collected 30 cm from a nest. (b) Soil samples were placed in an acrylic cage to dry in the sunlight for at least four hours. (c) The dried samples were sieved to classify soil particles by size. (d) The total weight of the sieve residues was compared with the weight of the original sample.”

**Added lines 791–795:** “**Fig 3.** Examples of unsuccessful Kentish plover nests in Saemangeum, 2020. (a) Flooding events were the main cause of nest failure in this study. (b) Kentish plover nests were often depredated by predators.”

**Added lines 802–805:** “**Fig 5**. Results of the elbow and silhouette methods for cluster analysis. The optimal number of clusters could not be determined using the elbow method. Therefore, we referred to the silhouette method and set two clusters for cover rate factors (A) and three clusters for soil particle size factors (B).”

**Added lines 823–839:** “**Fig 9.** Examples of nest types of Kentish plovers. We categorized the nests into five types: (a) primarily composed of shells, (b) primarily composed of dry vegetation main, (c) composed of dry soil particles, (d) composed of mixed materials, and (e) primarily composed of sand. (f) We also identified two nests located on a rice field ridge paved with gravel. (g–h) Additionally, we observed a nest built within human footprints. A pair of Kentish plovers selected a human footprint for nesting on an exposed, arid riverbed, primarily using soil particles as nest materials.”

**Added lines 839–842:** “**Table 3.** Strength values of arcs in the nest site selection model. Higher values indicate greater data fitness and importance within the model.”

**Added lines 843–845:** “**Table 4.** The Bayesian information criterion (BIC) values of nodes in nest site selection model. Lower values indicated the higher necessity in a model.”

**Added lines 847–850:** “**Table 5.** Strength values of arcs in the nest fate model. Higher values indicate better data fitness and greater importance in the model.”

**Added lines 852–856:** “**Table 6.** Bayesian information criterion (BIC) values of nodes in the nest fate model. Lower values indicate higher necessity in the model.”

**Added lines 58–860:** “**Table 7.** Eigenvalues and variance in the PCA results of nest failure causes and environmental variables.”

**13. Regarding the permission to obtain data for the work**

We wrote the official document codes of entrance permission on field work sites from the institution of government.

**Changed lines 120–124: from** “authorization from the Saemangeum Project Office in the Korea Rural Community Corporation was required for our research activities (Official Document Number: College of Natural Sciences-984, Jan 23^th^ 2020).”

**To** “Accordingly, research permission was granted by the Saemangeum Project Office in the Korea Rural Community Corporation, which is the authorization to manage land access in the Saemangeum. The permission for research area access was officially granted (Official Document Number: College of Natural Sciences-984, January 23, 2020).”

**14. Regarding data availability and code sharing**

In supplementary files, we include both the full raw data and processed datasets used for our modelling and PCA. We also upload our code in other supporting files.

**Changed lines 764–747: from** “The datasets generated and/or analyzed during the current study are available from the corresponding author on reasonable request.” **To** “All data required to replicate the findings are provided as Supporting Information files, including both the full raw data and processed datasets used for statistical modelling.”

**15. Regarding supplementary files**

We moved all figures and tables of original “S1 file” into main manuscript. So, we changed the remained supplementary file name from “S2 file” to “S1 file”. We also include those captions into our revised manuscript. They located end of the file.

**Added lines 862–925: Supplementary captions**

**16. Deleted** “Kentish plovers exhibited a preference for nesting and showed higher nest success in areas with moderate vegetation compared to those with no vegetation or overly dense vegetation.” around line 31

**17. Regarding funding,**

The funding information of this study is same as:

“This study was supported by the National Research Foundation of Korea (NRF) grant funded by the Korean government [NRF - 2019R1F1A1061024]. The funders had no role in study design, data collection and analysis, decision to publish, or preparation of the manuscript.”
