# Supplementary material for: Estimating nesting habitat characteristics for the Kentish plover (Anarhynchus alexandrinus) with the effect of substrate and vegetation using a Bayesian network approach
Source: PLoS One. 2025 Jun 11;20(6):e0325750. doi: 10.1371/journal.pone.0325750 (PMC12157252; doi:10.1371/journal.pone.0325750)
Supplement: Attachment — Submitted filename: Responses to the Reviewers 2nd - Plos One.docx [file pone.0325750.s008.docx]

**Responses to the Reviewers’ comments**

We appreciate the suggestion of Reviewer #2 to reduce repetition and improve scientific clarity. In this revision, we carefully reviewed the manuscript structure and reduced overlapping content, especially within the Introduction and Discussion. Furthermore, all direct comments and tracked edits provided in the manuscript were fully addressed.

We also sincerely thank Reviewer #1 for their positive evaluation and kind comments. We are grateful that our revisions were well received and that the manuscript is now considered suitable for publication.

**Responding to the comments of Reviewer #2**

1. Changed line 19 from “habitation” to “habitat”

2. Needs clarity on this

We removed pronoun to clarify this sentence and used clear terms to reconstruct this sententce.

🡪 Changed lines 20–21 from “To conserve shorebird populations, it is necessary to understand how artificial coastal areas can be used these species.” to “The conservation of shorebird populations thus necessitates understanding the utilization pattern of artificial coastal habitats by these birds.”

3. Multi layered would be a better option

🡪 Changed line 38 from “multifaceted” to “multi-layered”

4. What does optimization means, its relative

🡪 Changed line 39 from “to optimize” to “to restore appropriate”

5. Added line 47: for

6. Is it possible to return to the natural state?

We agree that this word would be felt as overexpression.

🡪 Deleted around line 47: “and returning them to their natural state”

7. Same as objective 1

We edited objective 1 instead of highlighting lines because we thought detailed explanations should be located in research necessity sections, not objective sections.

🡪 Changed lines 89–91 from “to examine the differences in environmental factors, including substrate type and vegetation cover, between nest and non-nest sites and successful and unsuccessful nest sites of Kentish plovers” To “to examine the differences in the environmental factors of microhabitat between nest and non-nest sites and successful and unsuccessful nest sites of Kentish plovers”

8. This paragraph can go above the objectives part

🡪 Changed lines from 77–81 to 89–93: “The main study objectives were: (1) to examine the differences in the environmental factors of microhabitat between nest and non-nest sites and successful and unsuccessful nest sites of Kentish plovers; (2) to estimate the characteristics in vegetation cover and substrate particle sizes; and (3) to provide insights into optimal artificial coastal habitats for Kentish plovers using modelling approaches, thus informing habitat restoration and conservation strategies.”

9. Already mentioned above

🡪 Changed lines 115–116 from “which are significant habitats for shorebirds and have been designated as UNESCO World Natural Heritage Sites. This further underscores the ecological significance of this region.” To “is a UNESCO World Natural Heritage Site.”

10. Deleted around line 122: “. The permission for research area access was officially granted”

11. Changed line 128 from “as well as” To “with”

12. Changed line 129–130 from “. The dominant halophyte species in tidal flats were” To “dominated by species like”

13. Added line 144: “a”

14. Deleted around line 148: “nest”

15. Changed line 153 from “for measuring” To “to measure”

16. Added line 180: “between”

17. Changed line 180 from “Onto” to “To”

18. Changed line 198 from “difine” to “define”

19. Added line 210: “the”

20. Added line 212: “to”

21. Deleted around line 251: “we”

22. Changed line 257 from “was” to “were”

23. Changed line 264 from “gravels” to “gravel”

24. Changed line 336 from “In particular, soil” to “Soil”

25. Deleted around line 343: “did”

26. Added line 350: “highlight”

27. Changed line 359 from “most closed” to “closest”

28. Changed line 366 from “speculate” to “assume”

29. Changed line 387 from “sites” to “site”

30. Changed line 392 from “plovers” to “plover’s”

31. Deleted around line 395: “rather”

32. Changed line 400 from “allow incubating Kentish plovers better” to “allows incubating Kentish plovers”

33. Changed line 404 from “was” to “were”

34. Can we call with dense as the size of the nest and vegetation is so small. Better define in the methods what is sparse, dense vegetation etc

We demonstrated the ‘absent’ and ‘dense’ of vegetation in a prior paragraph in the discussion. However, to improve the understanding of readers, we included the categorization of the vegetation node in the sentence.

🡪 Changed line 404–405 from “either ‘absent’ of ‘dense’” To “either ‘absent (0%)’ of ‘dense (>90%)’”.

35. Changed line 450 from “focus” to “focused”

36. Changed lines 456–457 from “for ecological characteristics in dynamic and stochastic ecosystems, without prematurely excluding ciritical factors.” To “for characteristics in dynamic and stochastic ecosystems, without prematurely excluding critical factors.”

37. Changed line 466 from “a priori” to “a prior”

38. Changed line 479 from “increase” to “increased”

39. Changed line 481 from “sites” to “site’

40. Changed line 486 from “lined” to “linked”

41. Added line 493: “the”

42. Changed line 498 from “on a global scale” to “globally”

43. A similar sort of sentence is there throughout the discussion part. It would be better to discuss the results, followed by limitations and then suggestions. The authors can reduce a lot of text by simplifying the repeated contents.

We confirmed that this sentence is similar to the sentences below. Therefore, we deleted this sentence. However, the necessity of long-term monitoring was first mentioned in the last section of our discussion. Thus, we had to include this text in this location.

🡪 Deleted around line 498: “to gain a more comprehensive understanding of the effect of gradual coastal environmental changes on their breeding behaviours, further investigations based on long-term datasets are necessary.”

44. Changed line 851 from “results” to “result”
