# Supplementary material for: Estimating nesting habitat characteristics for the Kentish plover (Anarhynchus alexandrinus) with the effect of substrate and vegetation using a Bayesian network approach
Source: PLoS One. 2025 Jun 11;20(6):e0325750. doi: 10.1371/journal.pone.0325750 (PMC12157252; doi:10.1371/journal.pone.0325750)
Supplement: Peer review history

# Author comment: Author response to Decision Letter 0

**DOI:** 10.1371/journal.pone.0325750.r001

21 Jan 2025

# Peer review: Decision Letter 0

**DOI:** 10.1371/journal.pone.0325750.r002

**Authors:** Aarif Muhammed

Dear Dr. Sung,

Thank you for submitting your manuscript to PLOS ONE. After careful consideration, we feel that it has merit but does not fully meet PLOS ONE’s publication criteria as it currently stands. Therefore, we invite you to submit a revised version of the manuscript that addresses the points raised during the review process.

Please submit your revised manuscript by Apr 26 2025 11:59PM. If you will need more time than this to complete your revisions, please reply to this message or contact the journal office at plosone@plos.org . When you're ready to submit your revision, log on to https://www.editorialmanager.com/pone/ and select the 'Submissions Needing Revision' folder to locate your manuscript file.

- A rebuttal letter that responds to each point raised by the academic editor and reviewer(s). You should upload this letter as a separate file labeled 'Response to Reviewers'.
- A marked-up copy of your manuscript that highlights changes made to the original version. You should upload this as a separate file labeled 'Revised Manuscript with Track Changes'.
- An unmarked version of your revised paper without tracked changes. You should upload this as a separate file labeled 'Manuscript'.

If you would like to make changes to your financial disclosure, please include your updated statement in your cover letter. Guidelines for resubmitting your figure files are available below the reviewer comments at the end of this letter.

If applicable, we recommend that you deposit your laboratory protocols in protocols.io to enhance the reproducibility of your results. Protocols.io assigns your protocol its own identifier (DOI) so that it can be cited independently in the future. For instructions see: https://journals.plos.org/plosone/s/submission-guidelines#loc-laboratory-protocols . Additionally, PLOS ONE offers an option for publishing peer-reviewed Lab Protocol articles, which describe protocols hosted on protocols.io. Read more information on sharing protocols at https://plos.org/protocols?utm_medium=editorial-email&utm_source=authorletters&utm_campaign=protocols .

We look forward to receiving your revised manuscript.

Kind regards,

Aarif Muhammed, Ph.D.

Academic Editor

PLOS ONE

Journal Requirements:

When submitting your revision, we need you to address these additional requirements.

1. Please ensure that your manuscript meets PLOS ONE's style requirements, including those for file naming. The PLOS ONE style templates can be found at https://journals.plos.org/plosone/s/file?id=wjVg/PLOSOne_formatting_sample_main_body.pdf and https://journals.plos.org/plosone/s/file?id=ba62/PLOSOne_formatting_sample_title_authors_affiliations.pdf

2. In your Methods section, please provide additional information regarding the permits you obtained for the work. Please ensure you have included the full name of the authority that approved the field site access and, if no permits were required, a brief statement explaining why.

3. Please note that PLOS ONE has specific guidelines on code sharing for submissions in which author-generated code underpins the findings in the manuscript. In these cases, we expect all author-generated code to be made available without restrictions upon publication of the work. Please review our guidelines at https://journals.plos.org/plosone/s/materials-and-software-sharing#loc-sharing-code and ensure that your code is shared in a way that follows best practice and facilitates reproducibility and reuse.

4. Thank you for stating the following financial disclosure:

This study was supported by the National Research Foundation of Korea (NRF) grant funded by the Korean government [NRF - 2019R1F1A1061024]

Please state what role the funders took in the study. If the funders had no role, please state: "The funders had no role in study design, data collection and analysis, decision to publish, or preparation of the manuscript."

If this statement is not correct you must amend it as needed.

Please include this amended Role of Funder statement in your cover letter; we will change the online submission form on your behalf.

5. We note that you have indicated that there are restrictions to data sharing for this study. PLOS only allows data to be available upon request if there are legal or ethical restrictions on sharing data publicly. For more information on unacceptable data access restrictions, please see http://journals.plos.org/plosone/s/data-availability#loc-unacceptable-data-access-restrictions.

Before we proceed with your manuscript, please address the following prompts:

a) If there are ethical or legal restrictions on sharing a de-identified data set, please explain them in detail (e.g., data contain potentially identifying or sensitive patient information, data are owned by a third-party organization, etc.) and who has imposed them (e.g., a Research Ethics Committee or Institutional Review Board, etc.). Please also provide contact information for a data access committee, ethics committee, or other institutional body to which data requests may be sent.

b) If there are no restrictions, please upload the minimal anonymized data set necessary to replicate your study findings to a stable, public repository and provide us with the relevant URLs, DOIs, or accession numbers. For a list of recommended repositories, please see

https://journals.plos.org/plosone/s/recommended-repositories. You also have the option of uploading the data as Supporting Information files, but we would recommend depositing data directly to a data repository if possible.

We will update your Data Availability statement on your behalf to reflect the information you provide.

6. In the online submission form, you indicated that data available on request from the author.

All PLOS journals now require all data underlying the findings described in their manuscript to be freely available to other researchers, either 1. In a public repository, 2. Within the manuscript itself, or 3. Uploaded as supplementary information.

This policy applies to all data except where public deposition would breach compliance with the protocol approved by your research ethics board. If your data cannot be made publicly available for ethical or legal reasons (e.g., public availability would compromise patient privacy), please explain your reasons on resubmission and your exemption request will be escalated for approval.

7. We note that Figure 1 in your submission contain [map/satellite] images which may be copyrighted. All PLOS content is published under the Creative Commons Attribution License (CC BY 4.0), which means that the manuscript, images, and Supporting Information files will be freely available online, and any third party is permitted to access, download, copy, distribute, and use these materials in any way, even commercially, with proper attribution. For these reasons, we cannot publish previously copyrighted maps or satellite images created using proprietary data, such as Google software (Google Maps, Street View, and Earth). For more information, see our copyright guidelines: http://journals.plos.org/plosone/s/licenses-and-copyright.

We require you to either (1) present written permission from the copyright holder to publish these figures specifically under the CC BY 4.0 license, or (2) remove the figures from your submission:

a. You may seek permission from the original copyright holder of Figure 1 to publish the content specifically under the CC BY 4.0 license.

We recommend that you contact the original copyright holder with the Content Permission Form (http://journals.plos.org/plosone/s/file?id=7c09/content-permission-form.pdf) and the following text:

“I request permission for the open-access journal PLOS ONE to publish XXX under the Creative Commons Attribution License (CCAL) CC BY 4.0 (http://creativecommons.org/licenses/by/4.0/). Please be aware that this license allows unrestricted use and distribution, even commercially, by third parties. Please reply and provide explicit written permission to publish XXX under a CC BY license and complete the attached form.”

Please upload the completed Content Permission Form or other proof of granted permissions as an "Other" file with your submission.

In the figure caption of the copyrighted figure, please include the following text: “Reprinted from [ref] under a CC BY license, with permission from [name of publisher], original copyright [original copyright year].”

b. If you are unable to obtain permission from the original copyright holder to publish these figures under the CC BY 4.0 license or if the copyright holder’s requirements are incompatible with the CC BY 4.0 license, please either i) remove the figure or ii) supply a replacement figure that complies with the CC BY 4.0 license. Please check copyright information on all replacement figures and update the figure caption with source information. If applicable, please specify in the figure caption text when a figure is similar but not identical to the original image and is therefore for illustrative purposes only.

The following resources for replacing copyrighted map figures may be helpful:

USGS National Map Viewer (public domain): http://viewer.nationalmap.gov/viewer/

The Gateway to Astronaut Photography of Earth (public domain): http://eol.jsc.nasa.gov/sseop/clickmap/

Maps at the CIA (public domain): https://www.cia.gov/library/publications/the-world-factbook/index.html and https://www.cia.gov/library/publications/cia-maps-publications/index.html

NASA Earth Observatory (public domain): http://earthobservatory.nasa.gov/

Landsat: http://landsat.visibleearth.nasa.gov/

USGS EROS (Earth Resources Observatory and Science (EROS) Center) (public domain): http://eros.usgs.gov/#

Natural Earth (public domain): http://www.naturalearthdata.com/

8. Please include captions for your Supporting Information files at the end of your manuscript, and update any in-text citations to match accordingly. Please see our Supporting Information guidelines for more information: http://journals.plos.org/plosone/s/supporting-information.

[Note: HTML markup is below. Please do not edit.]

Reviewers' comments:

Reviewer's Responses to Questions

**Comments to the Author**

1. Is the manuscript technically sound, and do the data support the conclusions?

Reviewer #1: Yes

Reviewer #2: Partly

Reviewer #3: Yes

**********

2. Has the statistical analysis been performed appropriately and rigorously?

Reviewer #1: Yes

Reviewer #2: Yes

Reviewer #3: Yes

**********

3. Have the authors made all data underlying the findings in their manuscript fully available?

The PLOS Data policy

Reviewer #1: Yes

Reviewer #2: Yes

Reviewer #3: Yes

**********

4. Is the manuscript presented in an intelligible fashion and written in standard English?

Reviewer #1: Yes

Reviewer #2: No

Reviewer #3: Yes

**********

Reviewer #1: Upon reviewing the manuscript, I found several areas where improvements are necessary. Below

is a summary of my observations and suggested revisions:

Major Revision Suggestions

1. Clarity and Focus

o The manuscript occasionally veers into overly detailed descriptions that can

distract from the main points. Streamline the text to ensure the central

findings remain the focus. For example:

▪ Introduction:Clearly define the research gap in the opening paragraph

instead of detailed background information.

▪ Discussion: Focus more on the implications of findings rather than

repeating results.

2. Figures and Data

o Ensure all figures (e.g., Fig 1, 3B) are cited in the text and are adequately

explained. Some figures lack detailed captions, making it difficult to

understand them independently.

o Consider reformatting the results section to integrate tables or figures more

effectively to support key findings.

3. Methodology

o Add clarity regarding statistical methods. While Bayesian Network (BN)

modeling is well-presented, details about the assumptions or limitations of

the approach should be expanded in the discussion.

o Specify why the Mangyeong and Dongjin sites were chosen over other possible

reclaimed lands. This strengthens the argument for the study’s relevance.

4. Grammar and Language

o Several grammatical errors and awkward phrases throughout the text need

revision for fluency. Examples include:

▪ "Because, the parent Kentish plovers…" should be "This is because

parent Kentish plovers…"

▪ Replace "ceaseless habitation loss" with "continuous habitat loss."

o Consistent terminology: Ensure consistent use of terms like "Kentish plover"

versus "Kentish Plovers."

5. Ethics Statement

o Expand the Ethics section slightly to include how non-invasive methods

minimized disturbances to Kentish plovers.

6. Conclusion

o The conclusion should emphasize how the study's findings are applicable to

global conservation strategies for shorebirds. Currently, it focuses too

narrowly on Saemangeum.

Reviewer #2: The introduction part has to be improved drastically. As of now, it requires the removal of repetitions throughout the text in those areas. Again, no flow of contents and objectives.

Many of the methods, introductions, and some results should be properly placed.

Discussion could be based on your findings.

Overall, the article could be reduced a lot, as most are unwanted repetitions.

English usage could be improved vastly.

Reviewer #3: Title:

The current title is lengthy. Please revise it to a maximum of 20 words.

Remove "reclaimed" and "and geological criteria" from both the main title and any subtitles, because it falls outside the scope of this study

Keywords:

Ensure keywords are unique and do not duplicate terms already present in the title or abstract.

Methods:

• Include a figure or map illustrating the study area's composition (e.g., natural areas in green, anthropogenic areas in grey, aquatic areas in blue).

• Provide a detailed description of the materials used.

• Add photographs of the study area, materials, and some nests.

• Review the bibliographic citation for the R software usage.

Results:

I was unable to access the supplementary material.

To address this, consider incorporating a table or figure directly into the manuscript to illustrate key statistical data, enabling readers to understand the results without relying on supplementary files.

Discussion:

The study does not directly compare reclaimed versus non-reclaimed lands. Therefore, remove "reclaimed" from the title.

Consider merging the final two sections (Recommendation and importance on the construction of artificial coastal breeding habitats for Kentish plovers and Future studies regarding nesting sites of Kentish plovers) into a maximum of three paragraphs. This would streamline the presentation, minimize repetition of information from the introduction, and allow for focused recommendations.

Conclusion:

Rewrite the conclusion, focusing solely on the findings specific to the study area in Korea.

Conclude with a statement emphasizing the importance of conserving the nesting areas.

**********

what does this mean? ). If published, this will include your full peer review and any attached files.

If you choose “no”, your identity will remain anonymous but your review may still be made public.

**Do you want your identity to be public for this peer review?** For information about this choice, including consent withdrawal, please see our Privacy Policy

Reviewer #1: **Yes: ** Taslima Sheikh

Reviewer #2: **Yes: ** Byju H

Reviewer #3: No

**********

[NOTE: If reviewer comments were submitted as an attachment file, they will be attached to this email and accessible via the submission site. Please log into your account, locate the manuscript record, and check for the action link "View Attachments". If this link does not appear, there are no attachment files.]

While revising your submission, please upload your figure files to the Preflight Analysis and Conversion Engine (PACE) digital diagnostic tool, https://pacev2.apexcovantage.com/ . PACE helps ensure that figures meet PLOS requirements. To use PACE, you must first register as a user. Registration is free. Then, login and navigate to the UPLOAD tab, where you will find detailed instructions on how to use the tool. If you encounter any issues or have any questions when using PACE, please email PLOS at figures@plos.org . Please note that Supporting Information files do not need this step.

Attachment Submitted filename: Taslima Reviewer Plos One.pdf

Attachment Submitted filename: Kentish_plover_nest_modelingreview BH.docx

Attachment Submitted filename: Dear Editor.pdf

# Author comment: Author response to Decision Letter 1

**DOI:** 10.1371/journal.pone.0325750.r003

17 Apr 2025

We would like to express our gratitude for your review and the detailed comments you provided. We have modified the manuscript according to your instructions and attempted to resolve all issues you mentioned. In this response, we will address each of your concerns point by point.

Attachment Submitted filename: Resonses to the Reviewers - Plos One.docx

# Peer review: Decision Letter 1

**DOI:** 10.1371/journal.pone.0325750.r004

**Authors:** Aarif Muhammed

Dear Dr. Sung,

Thank you for submitting your manuscript to PLOS ONE. After careful consideration, we feel that it has merit but does not fully meet PLOS ONE’s publication criteria as it currently stands. Therefore, we invite you to submit a revised version of the manuscript that addresses the points raised during the review process.

plosone@plos.org . When you're ready to submit your revision, log on to https://www.editorialmanager.com/pone/ and select the 'Submissions Needing Revision' folder to locate your manuscript file.

- A rebuttal letter that responds to each point raised by the academic editor and reviewer(s). You should upload this letter as a separate file labeled 'Response to Reviewers'.
- A marked-up copy of your manuscript that highlights changes made to the original version. You should upload this as a separate file labeled 'Revised Manuscript with Track Changes'.
- An unmarked version of your revised paper without tracked changes. You should upload this as a separate file labeled 'Manuscript'.

If you would like to make changes to your financial disclosure, please include your updated statement in your cover letter. Guidelines for resubmitting your figure files are available below the reviewer comments at the end of this letter.

If applicable, we recommend that you deposit your laboratory protocols in protocols.io to enhance the reproducibility of your results. Protocols.io assigns your protocol its own identifier (DOI) so that it can be cited independently in the future. For instructions see: https://journals.plos.org/plosone/s/submission-guidelines#loc-laboratory-protocols . Additionally, PLOS ONE offers an option for publishing peer-reviewed Lab Protocol articles, which describe protocols hosted on protocols.io. Read more information on sharing protocols at https://plos.org/protocols?utm_medium=editorial-email&utm_source=authorletters&utm_campaign=protocols .

We look forward to receiving your revised manuscript.

Kind regards,

Aarif K Muhammed, Ph.D.

Academic Editor

PLOS ONE

Journal Requirements:

Please review your reference list to ensure that it is complete and correct. If you have cited papers that have been retracted, please include the rationale for doing so in the manuscript text, or remove these references and replace them with relevant current references. Any changes to the reference list should be mentioned in the rebuttal letter that accompanies your revised manuscript. If you need to cite a retracted article, indicate the article’s retracted status in the References list and also include a citation and full reference for the retraction notice.

[Note: HTML markup is below. Please do not edit.]

Reviewers' comments:

Reviewer's Responses to Questions

**Comments to the Author**

Reviewer #1: All comments have been addressed

Reviewer #2: (No Response)

**********

2. Is the manuscript technically sound, and do the data support the conclusions?

Reviewer #1: Yes

Reviewer #2: Partly

**********

3. Has the statistical analysis been performed appropriately and rigorously?

Reviewer #1: N/A

Reviewer #2: Yes

**********

4. Have the authors made all data underlying the findings in their manuscript fully available?

The PLOS Data policy

Reviewer #1: Yes

Reviewer #2: Yes

**********

5. Is the manuscript presented in an intelligible fashion and written in standard English?

Reviewer #1: No

Reviewer #2: No

**********

Reviewer #1: Well structured manuscript and I am happy that authors have followed all my suggestions in revised paper, I recommend this for publication in PLOS

Reviewer #2: I have attached the comments in the article itself.

General comments: a lot of repetition is still there. It's good to discuss the results, followed by limitations of the work and then suggestions. Also, English usage can be improved a lot as I can see lot of AI language usage could be changed to Scientific language.

**********

what does this mean? ). If published, this will include your full peer review and any attached files.

If you choose “no”, your identity will remain anonymous but your review may still be made public.

**Do you want your identity to be public for this peer review?** For information about this choice, including consent withdrawal, please see our Privacy Policy

Reviewer #1: **Yes: ** Taslima Sheikh

Reviewer #2: **Yes: ** Byju H

**********

[NOTE: If reviewer comments were submitted as an attachment file, they will be attached to this email and accessible via the submission site. Please log into your account, locate the manuscript record, and check for the action link "View Attachments". If this link does not appear, there are no attachment files.]

While revising your submission, please upload your figure files to the Preflight Analysis and Conversion Engine (PACE) digital diagnostic tool, https://pacev2.apexcovantage.com/ . PACE helps ensure that figures meet PLOS requirements. To use PACE, you must first register as a user. Registration is free. Then, login and navigate to the UPLOAD tab, where you will find detailed instructions on how to use the tool. If you encounter any issues or have any questions when using PACE, please email PLOS at figures@plos.org

Attachment Submitted filename: MS_Kentish_plover_nest_modeling - second review BH.docx

# Author comment: Author response to Decision Letter 2

**DOI:** 10.1371/journal.pone.0325750.r005

11 May 2025

We deeply appreciate the editors and three reviewers for their insightful comments on the manuscript entitled “Estimating nesting habitat characteristics for the Kentish Plover (Anarhynchus alexandrinus) with the effect of substrate and vegetation using a Bayesian network approach”. Their comprehensive comments were greatly helpful to improve this manuscript, and most of the comments were reasonable.

During the revision, we tried to actively reflect on every comment from the editors and the reviewers. Not only we changed contexts following their suggestions, but also we added further detail information in the manuscript. When we did not change contexts according to the reviewer’s suggestions, we provided my rational response to the reviewer’s comments in detail.

We appreciate the suggestion of Reviewer #2 to reduce repetition and improve scientific clarity. In this revision, we carefully reviewed the manuscript structure and reduced overlapping content, especially within the Introduction and Discussion. Furthermore, all direct comments and tracked edits provided in the manuscript were fully addressed.

We also sincerely thank Reviewer #1 for their positive evaluation and kind comments. We are grateful that our revisions were well received and that the manuscript is now considered suitable for publication.

Attachment Submitted filename: Responses to the Reviewers 2nd - Plos One.docx

# Peer review: Decision Letter 2

**DOI:** 10.1371/journal.pone.0325750.r006

**Authors:** Aarif Muhammed

Estimating nesting habitat characteristics for the Kentish Plover (Anarhynchus alexandrinus) with the effect of substrate and vegetation using a Bayesian network approach

PONE-D-25-03524R2

Dear Dr. Sung,

We are pleased to inform you that your manuscript has been judged scientifically suitable for publication and will be formally accepted for publication once it meets all outstanding technical requirements.

Within one week, you will receive an e-mail detailing the required amendments. When these have been addressed, you will receive a formal acceptance letter and your manuscript will be scheduled for publication.

An invoice will be generated when your article is formally accepted. Please note, if your institution has a publishing partnership with PLOS and your article meets the relevant criteria, all or part of your publication costs will be covered. Please make sure your user information is up-to-date by logging into Editorial Manager at Editorial Manager®  and clicking the ‘Update My Information' link at the top of the page. If you have any questions relating to publication charges, please contact our Author Billing department directly at authorbilling@plos.org.

If your institution or institutions have a press office, please notify them about your upcoming paper to help maximize its impact. If they’ll be preparing press materials, please inform our press team as soon as possible -- no later than 48 hours after receiving the formal acceptance. Your manuscript will remain under strict press embargo until 2 pm Eastern Time on the date of publication. For more information, please contact onepress@plos.org.

Kind regards,

Aarif K Muhammed, Ph.D (Zoology); Ph.D (Ecology)

Academic Editor

PLOS ONE

Additional Editor Comments (optional):

The authors have incorporated all the major and minor comments provided by the three reviewers during the two phases of review. The reviewers have thoroughly evaluated the manuscript and have given their final approval for acceptance. I have also verified that all the suggested revisions were appropriately addressed. Therefore, I am pleased to accept the manuscript. Congratulations on your efforts!

Reviewers' comments:

Reviewer's Responses to Questions

**Comments to the Author**

Reviewer #2: All comments have been addressed

**********

2. Is the manuscript technically sound, and do the data support the conclusions?

Reviewer #2: Yes

**********

3. Has the statistical analysis been performed appropriately and rigorously?

Reviewer #2: Yes

**********

4. Have the authors made all data underlying the findings in their manuscript fully available?

The PLOS Data policy

Reviewer #2: Yes

**********

5. Is the manuscript presented in an intelligible fashion and written in standard English?

Reviewer #2: Yes

**********

Reviewer #2: The article has been improved to a great extent, especially in the introduction and discussion, avoiding the repetitions. This is a good study that needs to be published, as Kentish Plovers can be considered an indicator species in the Korean Peninsula.

**********

what does this mean? ). If published, this will include your full peer review and any attached files.

If you choose “no”, your identity will remain anonymous but your review may still be made public.

**Do you want your identity to be public for this peer review?** For information about this choice, including consent withdrawal, please see our Privacy Policy

Reviewer #2: **Yes: ** Byju H

**********

# Editor report: Acceptance letter

**DOI:** 10.1371/journal.pone.0325750.r007

**Authors:** Aarif Muhammed

PONE-D-25-03524R2

PLOS ONE

Dear Dr. Sung,

I'm pleased to inform you that your manuscript has been deemed suitable for publication in PLOS ONE. Congratulations! Your manuscript is now being handed over to our production team.

At this stage, our production department will prepare your paper for publication. This includes ensuring the following:

* All references, tables, and figures are properly cited

* All relevant supporting information is included in the manuscript submission,

* There are no issues that prevent the paper from being properly typeset

You will receive further instructions from the production team, including instructions on how to review your proof when it is ready. Please keep in mind that we are working through a large volume of accepted articles, so please give us a few days to review your paper and let you know the next and final steps.

Lastly, if your institution or institutions have a press office, please let them know about your upcoming paper now to help maximize its impact. If they'll be preparing press materials, please inform our press team within the next 48 hours. Your manuscript will remain under strict press embargo until 2 pm Eastern Time on the date of publication. For more information, please contact onepress@plos.org.

If we can help with anything else, please email us at customercare@plos.org.

Thank you for submitting your work to PLOS ONE and supporting open access.

Kind regards,

PLOS ONE Editorial Office Staff

on behalf of

Dr. Aarif K Muhammed

Academic Editor

PLOS ONE
